# Diverse targets of *SMN2*-directed splicing-modulating small molecule therapeutics for spinal muscular atrophy

**DOI:** 10.1093/nar/gkad259

**Published:** 2023-04-07

**Authors:** Eric W Ottesen, Natalia N Singh, Diou Luo, Bailey Kaas, Benjamin J Gillette, Joonbae Seo, Hannah J Jorgensen, Ravindra N Singh

**Affiliations:** Department of Biomedical Sciences, Iowa State University, Ames, IA 50011, USA; Department of Biomedical Sciences, Iowa State University, Ames, IA 50011, USA; Department of Biomedical Sciences, Iowa State University, Ames, IA 50011, USA; Department of Biomedical Sciences, Iowa State University, Ames, IA 50011, USA; Department of Biomedical Sciences, Iowa State University, Ames, IA 50011, USA; Department of Biomedical Sciences, Iowa State University, Ames, IA 50011, USA; Department of Biomedical Sciences, Iowa State University, Ames, IA 50011, USA; Department of Biomedical Sciences, Iowa State University, Ames, IA 50011, USA

## Abstract

Designing an RNA-interacting molecule that displays high therapeutic efficacy while retaining specificity within a broad concentration range remains a challenging task. Risdiplam is an FDA-approved small molecule for the treatment of spinal muscular atrophy (SMA), the leading genetic cause of infant mortality. Branaplam is another small molecule which has undergone clinical trials. The therapeutic merit of both compounds is based on their ability to restore body-wide inclusion of *Survival Motor Neuron 2* (*SMN2*) exon 7 upon oral administration. Here we compare the transcriptome-wide off-target effects of these compounds in SMA patient cells. We captured concentration-dependent compound-specific changes, including aberrant expression of genes associated with DNA replication, cell cycle, RNA metabolism, cell signaling and metabolic pathways. Both compounds triggered massive perturbations of splicing events, inducing off-target exon inclusion, exon skipping, intron retention, intron removal and alternative splice site usage. Our results of minigenes expressed in HeLa cells provide mechanistic insights into how these molecules targeted towards a single gene produce different off-target effects. We show the advantages of combined treatments with low doses of risdiplam and branaplam. Our findings are instructive for devising better dosing regimens as well as for developing the next generation of small molecule therapeutics aimed at splicing modulation.

## Graphical Abstract

**Graphical Abstract:**
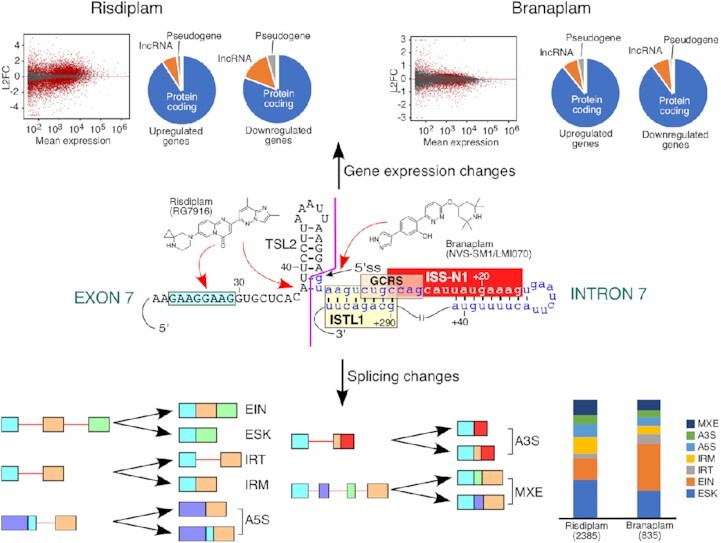
Diverse targets of *SMN2*-directed splicing-modulating small molecule therapeutics for spinal muscular atrophy.

## INTRODUCTION

**Figure 1.**
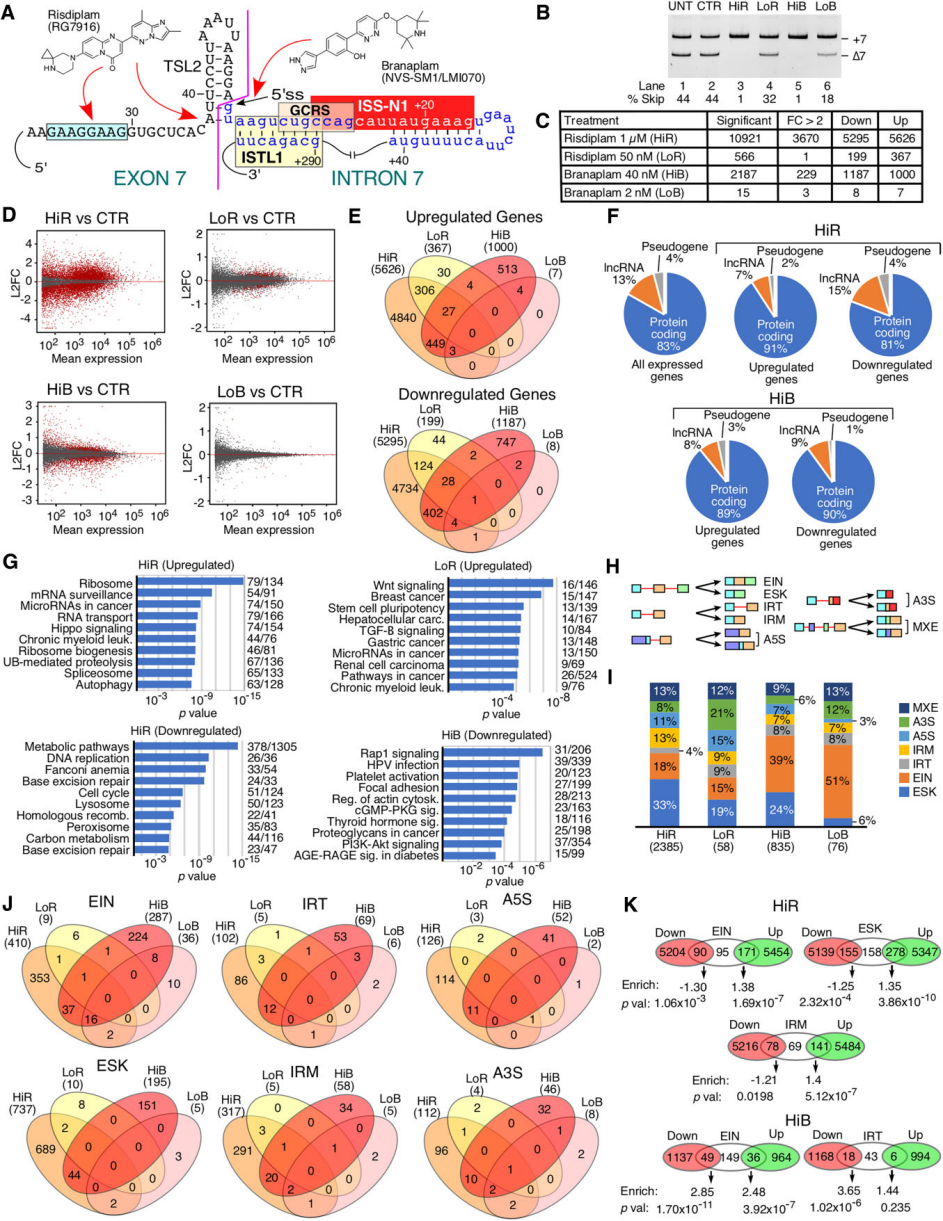
High doses of risdiplam and branaplam cause massive changes in the transcriptome. (**A**) Structures and proposed interaction sites of risdiplam and branaplam. Sequences and selected structures surrounding the 5′ss of *SMN* are shown. *Cis*-elements are labeled and indicated with colored boxes. Exonic sequences are shown in uppercase, intronic sequences in lowercase. Neutral numbering indicates position within exon 7, and positive numbering within intron 7. (**B**) Representative gel image showing the splicing pattern of *SMN2* exon 7 in GM03813 fibroblasts treated with different concentrations of small molecules. Splice isoform identities are indicated at the right side of the gel. Densitometric quantification of exon skipping relative to total RNA expression is indicated at the bottom. Abbreviations: UNT, untreated; CTR, 0.1% DMSO control; HiR, high concentration of risdiplam; LoR, low concentration of risdiplam; HiB, high concentration of branaplam; LoB, low concentration of branaplam. (**C**) Table summarizing transcriptomic changes detected by RNA-Seq after treatment with the indicated concentrations of the compounds. Significant genes indicate genes with Benjamini and Hochberg-adjusted *P*-value (adj. *P*) <0.05. FC >2 indicates genes with more than 2-fold up- or down-regulation. (**D**) MA plots depicting gene expression changes upon treatment with HiR, LoR, HiB or LoB compared with DMSO control. The *y*-axis represents the log_2_ fold change (L2FC) of treated cells compared with DMSO-treated control. The *x*-axis represents the mean expression in normalized read counts per gene. Red dots indicate genes with significantly altered expression levels (adj. *P* <0.05). Gray dots indicate unchanged or insignificant genes. (**E**) Four-way Venn diagrams examining the overlap between upregulated (top) and downregulated (bottom) genes after treatment with high and low doses of the compounds. Each treatment type along with the total number of affected genes are indicated at the top. (**F**) Proportion of protein-coding genes, pseudogenes and lncRNAs among differentially affected genes after HiR and HiB treatment. (**G**) The top 10 enriched KEGG pathways among upregulated genes after HiR treatment (top left) and LoR treatment (top right), and downregulated genes after HiR treatment (bottom left) and HiB treatment (bottom right). There were few enriched pathways among upregulated genes after HiB treatment or among downregulated genes after LoR treatment, and not enough affected genes after LoB treatment to perform pathway analysis. The category of expression change and treatment type are indicated at the top of each panel. Pathway names are given on the left side, and numbers of affected genes/total genes in each pathway are indicated on the right. The *x*-axis represents the *P*-value of enrichment. (**H**) Diagrammatic representation of each type of alternative splicing event under investigation. Exons are shown as colored boxes, introns as red lines. Abbreviations: EIN, exon inclusion; ESK, exon skipping; IRT, increased intron retention; IRM, improved intron removal; A5S, alternative 5′ splice site; A3S, alternative 3′ splice site; MXE, mutually exclusive/mixed exons. (**I**) Stacked bar chart representing the distribution of different types of alternative splicing events affected by high and low doses of the compounds. Color coding for each event type is indicated at the right side of the graph. The treatment and total number of affected events are shown at the bottom. The percentage of total events in each treatment is indicated for each category. (**J**) Four-way Venn diagrams comparing the alternative splicing events affected by each treatment. Each treatment along with the total number of affected alternative splicing events is indicated at the top. (**K**) Venn diagrams depicting the most significant overlaps between upregulated and downregulated genes and alternative splicing events after HiR and HiB treatment. For each overlap, enrichment was calculated and assigned a *P*-value relative to a random distribution using a hypergeometric test.

Spinal muscular atrophy (SMA), the leading genetic cause of infant mortality, results from low levels of the Survival Motor Neuron (SMN) protein due to deletions or mutations of the *SMN1* gene (1–3). SMN is a multifunctional protein involved in most aspects of RNA metabolism, including transcription, pre-mRNA splicing, mRNA trafficking, translation and stress granule formation (4). Consistently, most organs and tissues, such as the brain, gastrointestinal tract, heart, liver, lung, kidney, muscles, pancreas, testis and ovary are intrinsically affected in SMA (5). *SMN2*, a nearly identical copy of *SMN1* present in humans, cannot compensate for the loss of *SMN1* due to predominant skipping of exon 7 (6,7). Manipulation of splicing to restore *SMN2* exon 7 inclusion provides a promising therapeutic avenue for SMA (8). Skipping of *SMN2* exon 7 is driven primarily by a weak 3′ splice site (3′ss) created due to a critical C-to-T mutation at the sixth position (C6U substitution in RNA) of the exon coupled with an inherently weak 5′ss (7,9–14). The 5′ss of *SMN* exon 7 is surrounded by multiple negative *cis-*elements, including the terminal stem–loop structure 2 (TSL2), intronic splicing silencer N1 (ISS-N1), a GC-rich sequence (GCRS) and an internal-stem formed by a long-distance interaction (ISTL1) (Figure 1A) (14–20). Enhancing the accessibility of the 5′ss of exon 7 through either sequestration of ISS-N1/GCRS by an antisense oligonucleotide (ASO) or disruption of TSL2/ISTL1 has been shown to restore *SMN2* exon 7 inclusion (21). Also, forced recruitment of U1 small nuclear ribonucleoprotein (snRNP) at or downstream of the 5′ss of exon 7 has been found to promote exon 7 inclusion (22–24). Nusinersen (Spinraza™), an ISS-N1-targeting ASO delivered through intrathecal administration, became the first Food and Drug Administration (FDA)-approved therapy for SMA in 2016 (25,26). However, an ASO-based therapy has limitations linked to poor body-wide delivery/distribution and potential off-target effects (27,28). Novel chemical modifications and careful size selection are needed to further advance ASO-based therapies (27,29,30). Zolgensma (also named onasemnogene abeparvovec-xioi), a gene replacement therapy with adeno-associated virus (AAV)-mediated *SMN* gene delivery, became the second approved treatment for SMA (31). Gene therapy has its own disadvantages; for example, uncontrolled expression of SMN may sequester and/or mislocalize cellular transcripts and proteins that interact with SMN (32–34). Consistently, a recent study found toxicity associated with the long-term AAV9-mediated SMN overexpression (35,36).

Small molecules offer advantages of oral administration and excellent body-wide distribution (8,37). Risdiplam, an orally deliverable small molecule that restores *SMN2* exon 7 inclusion, was recently approved for the treatment of SMA (38,39). Branaplam, another small molecule capable of restoring *SMN2* exon 7 inclusion, has been considered for SMA therapy (40). However, clinical trials of branaplam were recently suspended due to adverse effects. Additional small molecules that promote *SMN2* exon 7 inclusion have been identified, although they have yet to enter clinical trials (41–43). Mechanisms by which small molecules stimulate *SMN2* exon 7 inclusion appear to be complex and not yet fully understood. For instance, several analogs of risdiplam were found to interact with GA-rich motifs of the single-stranded regions of both RNA and DNA (44–46). It has been proposed that binding of risdiplam to single-stranded regions of RNA helps recruit Far Upstream Element Binding Protein 1 (FUBP1) and its homolog KH-type Splicing Regulatory Protein (KHSRP) (45). Such recruitment may lead to sequestration of these proteins, affecting their targets. At least one analog of risdiplam has been shown to interact with a bulged adenosine residue present in the RNA:RNA duplex formed between the 5′ss of *SMN2* exon 7 and U1 snRNA (47). Risdiplam-mediated stabilization of bulged adenosine has been proposed to restore the accessibility of the U1-C zinc finger for interactions with the minor groove of the U1:5′ss duplex (47). Branaplam (synonym: NVS-SM1) has also been shown to bind to the interface formed between the 5′ss of *SMN2* exon 7 and U1 snRNP (48). It remains to be seen if such an interaction offers a high degree of specificity due to involvement of structural elements of *SMN2* pre-mRNA. Employing high-throughput RNA sequencing (RNA-Seq), an early study analyzed off-target effects of 500 nM C3, a risdiplam derivative, in SMA patient fibroblasts (37). While the authors confirmed significant change in expression of six genes, no validation was reported for any of the aberrantly spliced events (37). Another RNA-Seq study analyzed the off-target effects of 100 nM branaplam in SMA patient fibroblasts and used quantitative polymerase chain reaction (qPCR) to validate 11 aberrant splicing events (48). However, in most cases, qPCR results showing enhanced exon inclusion were not complemented with information about the corresponding decrease in exon skipping, making the findings somewhat difficult to interpret (48). A recent study compared the effects of 121 nM risdiplam (synonym used: compound 1), 605 nM risdiplam and 24 nM branaplam (38). However, the study was limited to examining only five off-target splicing events for *STRN3*, *FOXM1*, *APLP2*, *MADD* and *SLC25A17* by mapping of RNA reads, without independent validation of the aberrantly spliced products by qPCR or gel-based assays (38). Thus far, a systematic RNA-Seq analysis supported by complementary validations of different types of off-target effects associated with the small molecule therapeutics for SMA has not been performed.

Here we examine the transcriptome-wide effects of risdiplam and branaplam in SMA patient cells employing RNA-Seq. The study was aimed at capturing and comparing the broad spectrum of off-target effects caused by these compounds. Employing qPCR and semi-quantitative PCR, we validated the findings of RNA-Seq at three different concentrations of each compound. The high concentrations of both compounds caused massive perturbations of the transcriptome within 24 h of treatment. The extent of alterations was significantly higher in the case of risdiplam than branaplam. Genes with aberrant expression were associated with important cellular processes including DNA replication, cell cycle, RNA metabolism, cell signaling and metabolic pathways. Among adversely affected splicing events, we captured multiple incidences of aberrant exon inclusion, exon skipping, intron retention, intron removal and alternative splice site usage. While low concentrations of the compounds partially restored inclusion of *SMN2* exon 7, the off-target effects were reduced in the case of risdiplam and were almost non-existent in the case of branaplam. The results of complementary experiments using minigenes expressed in HeLa cells suggested that the motifs within exons and their immediate downstream intronic sequences modulate the action of risdiplam and branaplam. Our findings also reveal previously unreported novel exonic motifs associated with the stimulatory effect of risdiplam and branaplam on splicing of *SMN2* exon 7. We show the advantages of combined treatments with low doses of the compounds in modulating splicing of *SMN2* exon 7 while minimizing the potential off-target effects. Our findings are significant for devising the effective dosing regimens as well as for developing the next generation of small molecule therapeutics for the treatment of SMA.

## MATERIALS AND METHODS

### Cell culture

Tissue culture media and supplies were purchased from Life Technologies (Gibco brand) unless indicated otherwise. GM03813 Type I SMA patient fibroblasts were obtained from Coriell Cell Repositories and were grown in minimal essential medium (MEM; Gibco, catalog #10370) supplemented with 2 mM GlutaMAX-1 and 15% fetal bovine serum (FBS) at 37°C under 5% CO_2_. Human cervical adenocarcinoma (HeLa) cells were obtained from the American Type Culture Collection and were grown in Dulbecco's modified Eagle's medium (DMEM) supplemented with 10% FBS.

### Treatment with splicing-modulating compounds

Risdiplam and branaplam were purchased from MedChemExpress. To prepare stock solutions of risdiplam and branaplam, the compounds were resuspended in dimethylsulfoxide (DMSO) (Sigma) to a concentration of 5 mM. To fully dissolve the compounds, 1% hydrochloric acid was added dropwise to adjust the pH value. GM03813 cells were pre-plated 16 h before treatment at a density of 1.1 × 10^6^ cells per 100 mm cell culture dish or 4 × 10^5^ cells per 60 mm cell culture dish. The compounds were diluted in DMSO and then added to fresh cell culture medium so that the final concentration of DMSO was 0.1%. For treatments, cell culture medium was replaced with fresh medium containing the indicated concentration of DMSO alone, risdiplam or branaplam. Twenty-four hours later, cells were collected for RNA isolation followed by reverse transcription–PCR (RT–PCR) or RNA-Seq library preparation.

### Generation of minigenes and transfections

Hybrid minigenes were constructed using p*SMN2*ΔI6 (12). Briefly, the middle portion of the p*SMN2*ΔI6 minigene encompassing the last 121 nt of intron 6, the entire exon 7 and the first 165 nt of intron 7 of p*SMN2*ΔI6 was replaced with the exon of interest and its 100–200 nt long flanking intronic sequences (upstream and downstream). DNA fragments for cloning were generated by PCR using Phusion high fidelity DNA polymerase (Thermo Scientific) following the manufacturer's recommendations. Genomic DNA template was isolated from SH-SY5Y neuroblastoma cells using the Qiagen DNeasy blood and tissue kit. First we generated the *POMT2* hybrid minigene. In particular, the DNA fragment corresponding to *POMT2* exon 11B and its flanking intron sequences (amplified from genomic DNA) was ligated by PCR with the fragment containing the last 279 nt of *SMN2* intron 7 and the entire exon 8 (amplified from the pSMN2ΔI6 plasmid). As a result, a SalI restriction site was also introduced at the junction of *POMT2* intron 11B and *SMN2* intron 7. The hybrid fragment was then treated with KpnI and NotI, gel purified and inserted into pSMN2ΔI6 digested with the same restriction enzymes. The remaining hybrid minigenes were generated by replacing *POMT2* sequences with other sequences of interest using KpnI and SalI sites. Site-specific mutagenesis was achieved by two-step PCR using primers carrying specific base substitutions. The *MBNL1* minigene was constructed by amplifying an ∼2.5 kb genomic region from the beginning of exon 4 to the end of exon 6 of *MBNL1*. The resulting PCR amplification product was inserted into the pCI vector (Promega) using SalI and NotI sites. In order to facilitate the process of *MBNL1* exon 5 mutagenesis, AgeI and SacII sites were introduced 228 nt upstream and 217 nt downstream of exon 5, respectively, using a PCR-based approach. *SMN2* minigenes with site-specific mutations in exon 7 and partially randomized exon 7 sequences were generated similarly to as previously described (13). The identities of all minigene constructs were confirmed by Sanger sequencing.

Plasmids for transfections were purified with the QIAprep spin miniprep kit (Qiagen). Transfections were done using either HeLa cells in suspension (reverse transfection) or pre-plated HeLa cells/GM03813 cells. Briefly, HeLa cells were pre-plated at a density of 1.1 × 10^5^ cells per well of 24-well plates. GM03813 cells were pre-plated at a density of 1.8 × 10^5^ per well of 6-well plates. Sixteen hours later, cells were transfected with 0.05 μg of the minigene of interest and 0.45 μg of empty pCI neo vector in the case of HeLa cells, and 0.1 μg of the minigene of interest and 0.9 μg of empty pCI neo vector in the case of GM03813 cells, using Lipofectamine 2000 (Invitrogen) as per the manufacturer's recommendations. Aliquots of 0.5 and 1.0 μg of pCI neo alone were used as a transfection control for HeLa and GM03813 cells, respectively. For reverse transfection, HeLa cell suspensions containing 2.9 × 10^5^ cells per transfection were combined with DNA–Lipofectamine 2000 complexes. These cells were then plated in 24-well plates. Six hours after transfection, cell culture medium was replaced with fresh medium containing DMSO alone, risdiplam or branaplam, as described above. After a further 24 h (30 h after minigene transfection), cells were collected for RNA isolation.

### Randomization and *in vivo* selection of *FOXM1* exon 9

We introduced a 10 bp randomized region spanning from positions 70 to 79 of *FOXM1* exon 9 in the context of the hybrid minigene. We employed a three-fragment PCR-based approach in which a 59mer oligonucleotide encompassing the 10 nt randomized region in the middle and flanking portions of *FOXM1* exon 9 was used as template along with overlapping 5′ and 3′ fragments generated by PCR from the *FOXM1* exon 9 hybrid minigene. The complete fragment was then digested with SalI and KpnI and ligated into ∼500 ng of minigene digested with the same enzymes. Individual clones were isolated and sequenced, and then used for transfection as described above. At 16 h after transfection, cells were treated with 0.1% DMSO, 250 nM risdiplam or 10 nM branaplam for 6 h and then RNA was collected for RNA isolation followed by RT–PCR.

For *in vivo* selection, 20 μl of ligation reaction corresponding to ∼100 ng of plasmid was used directly for transfection in 6-well plates along with empty pCI vector to bring the final DNA concentration to 2 μg per well, as performed previously (13). Sixteen hours after transfection, cells were treated with 0.1% DMSO, 250 nM risdiplam or 10 nM branaplam for 6 h and then RNA was collected for RNA isolation followed by RT–PCR. First, minigene-derived transcripts were amplified by primers P1 and P2. Then, the band corresponding to inclusion of exon 9L was excised and purified by the ‘crush and soak’ method. The randomized region was then re-amplified using primers RR-F and RR-R to regenerate a double-stranded sequence corresponding to the original FOXM1-Random oligonucleotide (Supplementary Table S1). Amplification with flanking regions and ligation into the hybrid minigene were performed as described above. The entire process was repeated for a total of four cycles of selection.

### RT–PCR and qPCR

Total RNA was isolated using TRIzol reagent (Life Technologies) following the manufacturer's instructions. Unless noted otherwise, RNA samples were treated with RQ1 RNase-free DNase (Promega) following the manufacturer's instructions and then re-purified using phenol:chloroform extraction and ethanol precipitation. cDNA was generated using SuperScript III reverse transcriptase (Life Technologies) with 0.5–1.0 μg of total RNA per 5 μl reaction. The multi-exon skipping detection assay (MESDA) was performed similarly to as described previously (23,49–51). Of note, MESDA is a powerful technique to determine the relative abundance of multiple isoforms of *SMN* transcripts in a single experiment. Also, results of MESDA cannot be influenced by the amount of the template taken for PCR and/or the amount of sample loaded on the gel. cDNA templates for MESDA were generated using a gene-specific primer located within *SMN* exon 8. PCR for MESDA was carried out using primers located in exon 1 and the beginning of exon 8 (Supplementary Table S1). For semi-quantitative RT–PCR, cDNA was generated using oligo(dT)_12–18_ (Life Technologies). The PCR step was carried out using Taq DNA polymerase (New England Biolabs) and cDNA template following the manufacturer's recommendations. PCR products were separated on native polyacrylamide gels and visualized by ethidium bromide staining. Analysis and quantification of bands were performed using ImageJ software. Unless noted otherwise, PCR product identities were confirmed by sequencing as described previously (23). For qPCR, cDNA was prepared using random primers (Promega) and 0.5 μg of RNA per 5 μl of reverse transcriptase reaction. qPCR was performed using PowerUp SYBR green master mix (Life Technologies) on a QuantStudio 3 (Thermo Fisher) thermocycler according to the manufacturer's instructions. Relative expression was determined using the ΔΔCt method utilizing *OAZ1* as the normalizing gene for gene expression assays or primers targeting constitutively spliced regions of the gene of interest for intron retention assays. All primers were obtained from Integrated DNA Technologies. Their sequences are listed in Supplementary Table S1.

### Library generation and RNA-Seq

To confirm RNA integrity, TRIzol-isolated total RNA was characterized using an Agilent Bioanalyzer on an RNA nano chip (RIN ≥8). A 1 μg aliquot of total RNA was then subjected to rRNA depletion using the NEBNext rRNA depletion kit v2 (Human/Mouse/Rat). Libraries were generated from rRNA-depleted RNA using the NEBNext Ultra II directional RNA library prep kit for Illumina. Libraries were barcoded for multiplexing using NEBNext Dual Index oligos for Illumina. The size distribution of libraries was determined using an Agilent Bioanalyzer DNA 1000 chip and quantified using a Qubit fluorimeter. Libraries were pooled together and sequenced on an Illumina Novaseq 60000 using an S23 flow cell following a 100 cycle, paired-end protocol.

### RNA-Seq read mapping and bioinformatic analysis

Reads from RNA-Seq were mapped to the human reference genome build GRCh38 using HISAT2 (52). For differential expression, mapped reads were assigned to genes according to the Gencode v33 human transcriptome annotation (53) using the featureCounts script from the Subread software package (54). Differential expression was estimated using the DESeq2 R package (55). To identify differentially affected alternative splicing events, mapped reads were analyzed by rMATS (56). After initial identification, significant events were subjected to the following filtering criteria: false discovery rate (FDR) <0.05, ≥10 average junction reads supporting each isoform in at least one sample group, and a change in percentage spliced in (PSI) values of at least 0.1 for alternatively spliced exons or 0.05 for differential intron retention/removal.

Significantly enriched Kyoto Encyclopedia of Genes and Genomes (KEGG) pathways (57) were identified using WEBGestalt (webgestalt.org). Differentially expressed genes were categorized by gene type, chromosomal location and number of transcripts per gene using Ensembl BioMart to filter gene lists. As a reference list, we used all the genes with measurable expression (at least 10 reads per sample) in GM03813 cells. Transcription factors with enriched targets in differentially expressed genes were identified using ChEA3 (58). Splice site scores were calculated using the tools hosted at http://rulai.cshl.edu/new_alt_exon_db2/HTML/score.html. Enriched sequence motifs were identified using MEME (59).

## RESULTS

### Transcriptome-wide perturbations triggered by high concentrations of risdiplam and branaplam

In order to capture the transcriptome-wide perturbations caused by risdiplam and branaplam, we performed RNA-Seq on transcripts isolated from GM03813 Type I SMA patient fibroblasts treated with either compound for 24 h. Of note, these cells carry only the *SMN2* gene. To select concentrations of interest, we first treated cells with four different doses of each compound (from 10 nM to 1 μM for risdiplam and from 0.5 nM to 40 nM for branaplam) followed by examining *SMN2* exon 7 splicing by semi-quantitative PCR (Supplementary Figure S1A). We chose 1 μM risdiplam (hereafter referred to as HiR) and 40 nM branaplam (hereafter referred to as HiB) as high concentrations based on the near total inclusion of *SMN2* exon 7 (Figure 1B). For low concentrations, we chose 50 nM risdiplam (hereafter referred to as LoR) and 2 nM branaplam (hereafter referred to as LoB). The selected high and low concentrations were in the range used in previously reported studies (42,44,48). Recently, a high concentration of an ISS-N1-targeting ASO has been shown to trigger off-target inclusion of *SMN* exon 6B that is derived from an Alu element (28,60). Also, *SMN* exons 3, 5 and 7 undergo enhanced skipping under oxidative stress conditions (49,51). The results of our RNA-Seq and MESDA showed that neither compound had an appreciable effect on individual splicing of *SMN* exons 3, 5 and 6B (Supplementary Figure S1B, C). As indicated in Figure 1C, HiR altered expression of 10921 genes including 3670 genes with >2-fold change. Of these, roughly half were upregulated and half were downregulated (Figure 1C). Among highly expressed genes, there was an apparent bias towards up-regulation compared with down-regulation (Figure 1D). LoR altered (mostly upregulated) the expression of 566 genes, with only one of them showing >2-fold change (Figure 1C, D). HiB altered expression of 2187 genes, including 229 genes showing >2-fold change (Figure 1C). The altered expression was equally distributed between upregulated (1000) and downregulated (1187) genes (Figure 1C). However, highly expressed genes were more strongly downregulated than upregulated (Figure 1D). LoB produced changes in the expression of only a handful of genes (Figure 1C, D). For upregulated genes, the greatest overlap was between treatments with different doses of the same drug (Figure 1E). Of the 367 genes affected by LoR, >90% were also affected by HiR. All seven genes upregulated by LoB were also upregulated by HiB. In contrast, only 479 genes out of 5626 and 1000 genes upregulated by HiR and HiB, respectively, were common. Similarly, among downregulated genes, a greater overlap was found between LoR and HiR (153 genes, compared with 199 total LoR downregulated and 5295 HiR downregulated genes) and between LoB and HiB (7 genes, compared with 8 total LoB downregulated and 1187 total HiB downregulated genes) than between treatments with different drugs (Figure 1E).

Genes upregulated and downregulated by HiR and HiB were located on all chromosomes (Supplementary Figure S2). Of all the genes that are expressed in GM03813 cells, ∼83% are protein coding, ∼13% are long non-coding RNA (lncRNA) genes and ∼4% are pseudogenes (Figure 1F). In the category of genes upregulated by HiR, protein-coding genes became enriched (∼91%), while in the downregulated category, the relative proportion of affected genes showed only a slight change (Figure 1F). In the case of HiB, protein-coding genes were over-represented in both up- and downregulated categories (Figure 1F). Both HiR and HiB appeared to preferentially affect expression of genes that generate multiple alternative transcripts rather than genes with fewer alternative transcripts (Supplementary Figure S2B, E). We did not observe any significant overlap between enriched transcription factor target sites in HiR- and HiB-affected genes (Supplementary Figure S2C, F). We performed pathway enrichment analysis on up- and downregulated genes. Among genes upregulated by HiR, we observed a significant enrichment of genes involved in RNA maturation- and translation-related processes such as ribosome, mRNA surveillance, microRNAs (miRNAs) in cancer, RNA transport, ribosome biogenesis and spliceosome (Figure 1G). In addition, we noted enrichment of genes involved in ubiquitin-mediated proteolysis and autophagy, suggesting an effect on protein homeostasis (Figure 1G). Given the low number of genes upregulated by LoR, the nature of affected pathways was distinct from those observed with HiR. Among pathways that involved genes downregulated by HiR were cell cycle, DNA replication, base excision repair, homologous recombination, general metabolism (metabolic pathways, carbon metabolism) and organelle function (lysosome, peroxisome) (Figure 1G). No pathways were significantly enriched among genes upregulated by HiB. The most affected pathways in HiB-downregulated genes were cellular signaling (Rap1 signaling, cGMP–PKG signaling, thyroid hormone signaling, PI3K–Akt signaling and AGE–RAGE signaling in diabetes) and cytoskeletal dynamics (focal adhesion, regulation of actin cytoskeleton, Rap1 signaling) (Figure 1G).

We performed motif enrichment analysis using 200 nt long promoter sequences extracted from the 200 most upregulated and downregulated transcripts by HiR and HiB (Supplementary Figure S3A–D). We observed enrichment of a G-rich motif with interspersed A residues in genes upregulated by HiR, which bears a strong resemblance to the target sequence of risdiplam identified in the body of *SMN* exon 7 (46) as well as the binding site of several zinc finger-type transcription factors (Supplementary Figure S3E). In genes downregulated by HiR as well as in genes up- and downregulated by HiB, we observed enrichment of a GC-rich motif (Supplementary Figure S3B–D). Since the GC-rich motif was present in all of three categories of genes, we assume this to be a common promoter element in genes expressed in fibroblasts. In addition, genes downregulated by HiR had enrichment of an A-rich motif with interspersed G residues, although it showed less consistent conservation than the motif found in HiR-upregulated genes (Supplementary Figure S3C).

We analyzed seven types of alternative splicing events, namely exon skipping (ESK), exon inclusion (EIN), increased intron retention (IRT), improved intron removal (IRM), alternative 5′ss usage (A5S), alternative 3′ss usage (A3S) and mutually exclusive/mixed usage of neighboring exons (MXE) (Figure 1H). HiR altered 2385 splicing events, with the most common being ESK (33%) followed by EIN (18%), IRM (13%), MXE (13%), A5S (11%), A3S (8%) and IRT (4%) (Figure 1I). LoR caused modest changes in splicing events, with the most common being A3S and ESK (21% and 19%, respectively), followed by EIN (15%), A5S (15%), MXE (12%), IRM (9%) and IRT (9%). Of the 835 alternative splicing events triggered by HiB, 39% were EIN, followed by ESK at 24%. All other types of events were <10% each. LoB impacted 76 splicing events that were mostly skewed towards EIN, which constituted 51% of all events. MXE (13%) and A3S (12%) were the next most common events, and only a handful of events were associated with other types of alternative splicing. The majority of affected splicing events were distinct between HiR and HiB (Figure 1J). The most significant overlaps were found in EIN, with 54 events shared between HiR and HiB (13% of HiR- and 19% of HiB-triggered events), 24 shared between HiB and LoB (8% of HiB- and 67% of LoB-riggered events) and 16 were shared among HiR, HiB and LoB. We also observed a higher degree of overlap of IRM events triggered by HiR and HiB (7% of HiR-triggered events and 40% of all HiB-triggered events). The remainder of the events showed similar levels of overlap, ranging from 5% to 12% for HiR and from 15% to 25% for HiB. Aside from EIN, no events were prevalent enough in LoR or LoB to draw any definitive conclusions.

Of the 356 genes associated with EIN affected by HiR, the expression of 90 genes was downregulated, 95 genes remained unchanged and 171 genes were upregulated (Figure 1K). Using a hypergeometric test, we determined whether this correlation represented statistically significant under- or overenrichment (Figure 1K). Downregulated genes were under-represented 1.3-fold compared with random chance, while upregulated genes were over-represented 1.38-fold. This suggests that EIN events caused by HiR were strongly associated with increased transcript levels. Similarly, downregulated genes were under-represented in ESK-associated genes 1.25-fold, while upregulated genes were over-represented 1.35-fold (Figure 1K). For IRT and IRM events, only IRM showed a significant association with transcription up-regulation (1.4-fold enrichment, Figure 1K). A3S, A5S and MXE were not significantly enriched among downregulated or upregulated genes. In the case of branaplam, a large number of genes with altered expression levels were also coupled with aberrant splicing (Figure 1K). In particular, transcripts undergoing EIN were enriched among both downregulated (2.85-fold) and upregulated (2.48-fold) genes, and IRT events were strongly enriched (3.65-fold) among downregulated genes.

### Validation of expression of representative genes dysregulated by risdiplam and branaplam

**Figure 2.**
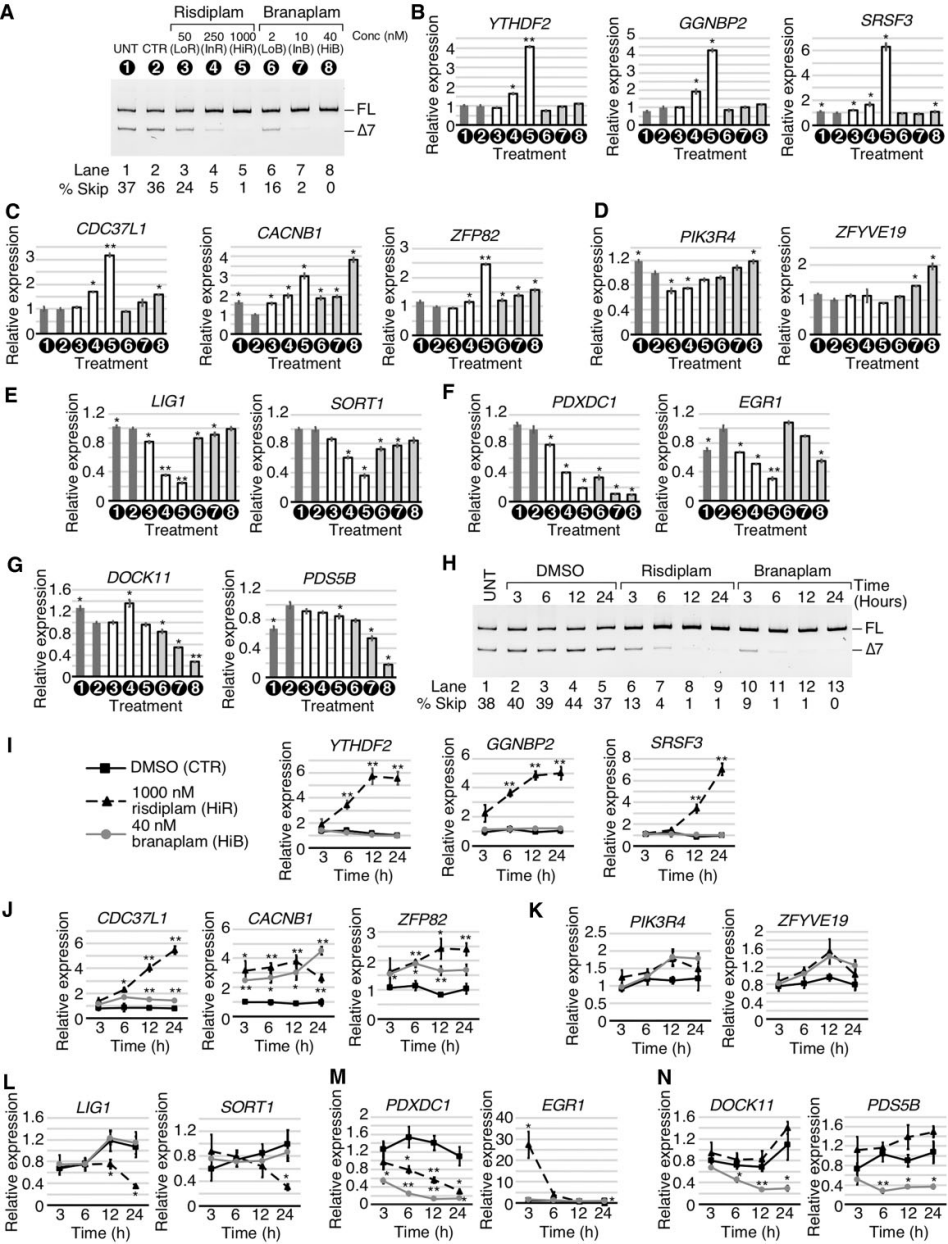
Risdiplam and branaplam affect gene expression in a concentration-dependent manner. (**A**) Representative gel image showing the splicing pattern of *SMN2* exon 7 in GM03813 fibroblasts treated with different concentrations of risdiplam and branaplam. Numbers in black circles represent treatment type and are used throughout the figure. Other labeling is the same as in Figure 1B. (**B**–**D)** qPCR results measuring the expression of specific genes that were upregulated by risdiplam treatment only (**B**), by both risdiplam and branaplam treatments (**C**) or by branaplam treatment only (**D**). The gene name is indicated above each graph. Treatment types are indicated under the *x*-axis. The *y*-axis represents the relative expression as compared with control (CTR) DMSO treatment. Error bars indicate the standard error of the mean (SEM); *n*= 3; *P <0.05; **P <0.01. (**E**–**G**) qPCR results measuring the expression of specific genes that were downregulated by risdiplam treatment only (**E**), by both risdiplam and branaplam treatments (**F**) or by branaplam treatment only (**G**). Labeling is the same as in (B–D). (**H**) Representative gel image showing the splicing pattern of *SMN2* exon 7 in GM03813 fibroblasts treated with HiR or HiB at different time points. Treatments and time are indicated at the top of the gel. Other labeling is the same as in Figure 1B. (**I–N**) qPCR results measuring the expression of the genes examined in (B–G) at different time points. Treatments are marked as indicated in (I). The *x*-axis represents time of treatment and the *y*-axis represents relative expression compared with untreated control. Error bars indicate the SEM; *n*= 3; *P <0.05; **P <0.01.

We validated the results of RNA-Seq by qPCR using 14 representative genes that were aberrantly expressed in the presence of HiR and/or HiB. For validation purposes, we used risdiplam at 1 μM, 250 nM and 50 nM as high (HiR), intermediate (InR) and low (LoR) concentrations, respectively. For branaplam, we used 40 nM, 10 nM and 2 nM as high (HiB), intermediate (InB) and low (LoB) concentrations, respectively (Figure 2A). Considering both compounds were dissolved in DMSO, for all comparisons we used DMSO control as base level expression. We included untreated control to monitor if DMSO itself had an effect on the expression of genes in question. We began with the examination of expression of three genes, *YTHDF2*, *GGNBP2* and *SRSF3*, which, according to RNA-Seq, were upregulated by HiR but not HiB. *YTHDF2* codes for an *N*-6-methyladenosine (m^6^A) ‘reader’ protein that binds m^6^A-modified RNAs and regulates their stability (61). Interestingly, lung adenocarcinoma cells have been found to display elevated expression of *YTHDF2* (62). The *GGNBP2* gene codes for a protein that interacts with gametogenetin during spermatogenesis; the protein is also associated with amyotrophic lateral sclerosis (ALS), ovarian cancer, breast cancer and chromosome 17Q12 deletion syndrome (63–67). *SRSF3* codes for SRp20 that is highly expressed in cancers (68). SRSF3 regulates alternative splicing and RNA stability; it also mediates m^6^A-dependent mRNA splicing and nuclear export along with YTHDC1 (69,70). Our qPCR validation confirmed ∼1.6- and >4-fold increases in *YTHDF2* expression by InR and HiR, respectively, whereas LoR as well as branaplam produced no effect (Figure 2B). *GGNBP2* levels were upregulated by ∼1.9- and ∼4.3-fold by InR and HiR, respectively (Figure 2B). *GGNBP2* levels were not significantly affected by LoR or any of the treatments with branaplam (Figure 2B). We observed up-regulation of *SRSF3* by risdiplam in a concentration-dependent manner, with HiR showing the most prominent change of ∼6.3-fold (Figure 2B). A negligible but statistically significant increase of *SRSF3* levels was also observed with HiB.

We next examined the expression of *CDC37L1*, *CACNB1* and *ZFP82* that, according to our RNA-Seq analysis, were upregulated by both compounds. *CDC37L1* codes for a tumor suppressor that acts by negatively regulating cyclin-dependent kinase 6 (71). *CACNB1* codes for the beta subunit of a voltage-dependent calcium channel and its dysregulation has been linked to pathological conditions, including malignant hyperthermia and headache (72). *ZFP82* codes for a zinc finger transcription factor that displays tumor suppressor properties (73). Our qPCR results validated strong up-regulation (∼3-fold) of *CDC37L1* by HiR, while InR and HiB induced an ∼1.5-fold increase in its expression (Figure 2C). *CACNB1* was upregulated by ∼3- and ∼4-fold by HiR and HiB, respectively (Figure 2C). Lower concentrations of the compounds appeared to also increase levels of *CACNB1*, although it was difficult to make definitive conclusions as DMSO treatment alone affected *CACNB1* expression (Figure 2C). While both HiR and HiB upregulated *ZFP82*, the effect was more pronounced with HiR (Figure 2C). Next we examined the expression of *PIK3R4* and *ZFYVE19* that were moderately upregulated by branaplam only (Figure 2D). *PIK3R4* codes for a protein involved in trafficking and autophagy, and missense mutation of *PIK3R4* is linked to ciliopathy (74). *ZFYVE19* codes for a zinc finger FYVE domain-containing protein. Mutations in the *ZFYVE19* gene are associated with liver dysfunction due to loss of cellular cilia (75). The qPCR results validated a slight increase in *PIK3R4* by HiB. In contrast, risdiplam treatments showed slight down-regulation of *PIK3R4* (Figure 2D). Of note, according to our RNA-Seq analysis, branaplam treatment in general induced far less transcription up-regulation of target genes than risdiplam (Figure 1D). In the case of *ZFYVE19*, both InB and HiB significantly upregulated its expression, while none of the risdiplam concentrations used had an appreciable effect (Figure 2D).

We validated the expression of two genes, namely *LIG1* and *SORT1*, that were downregulated by risdiplam but not branaplam as per our RNA-Seq analysis (Figure 2E). *LIG1* codes for a protein involved in joining of Okazaki fragments during DNA replication, and its mutation is associated with delayed growth, cancer and possibly immunodeficiency (76). *SORT1* codes for Sortilin, a protein that interacts with apolipoprotein B100 (apoB100) in the Golgi and regulates plasma low-density lipoprotein levels (77). Dysregulation of *SORT1* is associated with coronary artery diseases (78). Our qPCR results confirmed down-regulation of *LIG1* by risdiplam in a concentration-dependent manner, whereas the effect of branaplam on *LIG1* expression was minuscule (Figure 2E). *SORT1* was significantly downregulated by HiR and InR, while branaplam treatments triggered only slight reductions in the expression of this gene (Figure 2E). We tested the effect of the compounds on the expression of *PDXDC1* and *EGR1* shown to be downregulated by both HiR and HiB as per our RNA-Seq results (Figure 2F). *PDXDC1* codes for a protein that participates in phospholipid metabolism and is associated with elevated circulating lipid levels based on GWAS (genome-wide association studies) (78). *EGR1* codes for a transcription factor that controls cell growth and survival by targeting multiple tumor suppressors (79). Validation by qPCR confirmed that *PDXDC1* expression levels were strongly reduced by both compounds in a concentration-dependent manner, with ∼5-fold reduction by HiR and ∼10-fold reduction by HiB (Figure 2F). *EGR1* was strongly downregulated by InR and HiR as well as by HiB (Figure 2F). The effect of the low concentration of the compounds appeared to be somewhat skewed as DMSO itself slightly upregulated *EGR1* compared with untreated cells (Figure 2F). Finally, we verified the expression of *DOCK11* and *PDS5B* shown to be downregulated by branaplam by our RNA-Seq analysis (Figure 2G). *DOCK11* codes for a protein that is a member of the DOCK family of guanine nucleotide exchange factors. The protein regulates the actin cytoskeleton and cell motility. *DOCK11* dysregulation has been linked to immunodeficiency disorders (80). *PDS5B* codes for a protein required for pairing of sister chromatids during cell division, and down-regulation of *PDS5B* has been associated with breast cancer (81). Our qPCR results validated that branaplam downregulated *DOCK11* in a concentration-dependent manner, with the strongest effect (∼3-fold decrease) caused by HiB (Figure 2G). *PDS5B* was downregulated by ∼20% with InB and by ∼80% with HiB (Figure 2G).

To gain mechanistic insights, we examined the effect of HiR and HiB on expression of the representative genes at different time points using qPCR. While a quick response to a compound may support a direct and/or high affinity engagement with a target, the delayed response could be due to indirect and/or low affinity engagement with a target. We chose 3 h treatment as the earliest time point as both HiR and HiB induced a substantial increase in inclusion of *SMN2* exon 7 at this time (Figure 2H). Noticeably, only four out of 14 representative genes, namely *CACNB1*, *ZFP82*, *PDXDC1* and *EGR1*, displayed a significant change in expression at the 3 h time point (Figure 2I–N). Another four genes, *YTHDF2*, *GGNBP2*, *DOCK11* and *PDS5B*, showed a significant change in their expression levels after 6 h of treatment with the compound. Changes in expression of the remaining genes were observed only at or after 12 h of treatment. For many genes, the effect of the compounds plateaued after 12 h. However, *SRSF3*, *CDC37L*, *CACNB1*, *LIG1*, *SORT1* and *PDXDC1* showed the maximum change in expression at 24 h (Figure 2I, J, L). For genes affected by both HiR and HiB, we captured a differential effect of the compounds with respect to treatment duration. For example, during HiR treatment, *CACNB1* induction reached a maximum at the 12 h time point followed by a subsequent decline (Figure 2J). At the same time, treatment with HiB resulted in a progressive increase in expression of *CACNB1* from 3 h to 24 h of treatment. Notably, the strongest effect of HiR on *EGR1* expression was observed only at the early time point (3 h) followed by a drastic decline of the effect (Figure 2M). For mechanistic insights, we analyzed the region directly surrounding the transcription start sites (TSSs) of each gene confirmed to be affected by risdiplam and/or branaplam (Supplementary Figure S3). Many of these genes harbor stretches of purine-rich motifs within their TSS regions. However, we could not find a definitive correlation between the effect of the compounds on transcription and a specific TSS motif(s)

### Analysis and validation of exon inclusion events triggered by risdiplam and branaplam

In order to better understand the mechanisms behind the off-target splicing modulation by risdiplam and branaplam, we compared the conservation of sequences at the 5′ss of each exon whose inclusion was triggered by HiR and/or HiB (Supplementary Figure S4). Our analysis indicated sequence conservation from two bases upstream to six bases downstream of each exon/intron junction (Supplementary Figure S4A). The first six intronic bases matched the consensus 5′ss, GUAAGU. After the invariant GU dinucleotide, the next highest conservation was observed for G at the fifth intronic position, followed by As at the third and fourth intronic positions. U at the sixth intronic position had the lowest conservation. We also looked at the last two exonic positions (positions –2 and –1) (Supplementary Figure S4). In human exons, A at the –2 position and G at the –1 position are represented at >55% and ≥75% instances, respectively. Exons, inclusion of which was promoted by HiR, matched this consensus rather well; however, HiB-only sensitive exons had a noticeable enrichment of G at their –2 position and A at their –1 position (Supplementary Figure S4A). This enrichment was even stronger in the exons affected by both compounds.

We analyzed the splicing-relevant context for 30 exons (EIN events) strongly affected by HiR only, HiB only or both (Supplementary Figure S4B). These exons were present in genes of varying sizes, from 18.3 to 702 kb. Of the candidate exons we selected, the HiR targets tended to be located within larger genes, but this difference was not statistically significant. Likewise, exon positions within each gene varied widely, from the second exon to the 29th. Sizes of the affected exons and their flanking introns also showed no clear preference, with affected exons being as short as 11 bp and as long as 1193 bp, and flanking introns as short as 40 bp and as long as 61.9 kb (Supplementary Figure S4B). We also compared the strengths of the 3′ss and 5′ss of the exons in question. The mean scores of the constitutive 3′ss and 5′ss are 7.9 and 8.1, respectively. HiR targets had mean 3′ss and 5′ss scores of 3.9 and 7.0, respectively. Most targets had either a weak 3′ss or a weak 5′ss, but rarely both (Supplementary Figure S4B). In contrast, HiB targets had the mean 3′ss score of 6.6 and the mean 5′ss score of 7.0. Interestingly, for exons whose inclusion was promoted by both drugs, the mean 3′ss score was 7.9, the same as for constitutive exons, while the mean 5′ss score was 6.1 (Supplementary Figure S4B). This suggests that the functional overlap of the compounds may be centered around correction of a weak 5′ss. Notably, 8 of the 10 top risdiplam-only target exons had a G at the last position, whereas in the top branaplam-only targets, 3 had a G, 1 had a U and 6 had an A residue. Strikingly, of the top 10 targets of both compounds, all 10 had an A at the last position of the exon, suggesting that this position is important for the effect of both drugs (Supplementary Figure S4B). There was also a striking conservation of an A residue at the third intronic position, which was shared by most targets of branaplam, but not of risdiplam.

**Figure 3.**
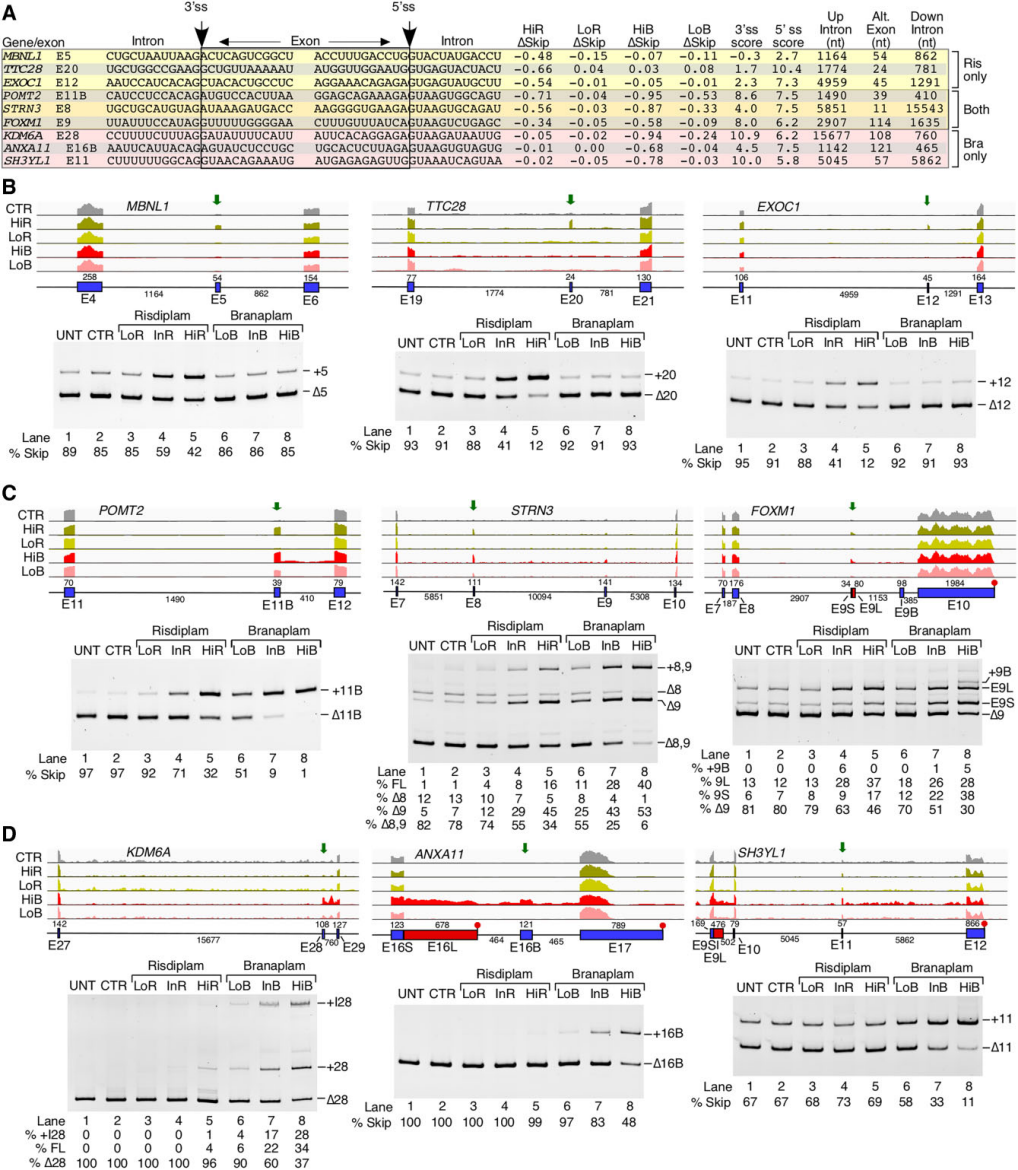
Risdiplam and branaplam promote off-target exon inclusion. (**A**) Table summarizing splicing-relevant information regarding the top candidate exons with increased inclusion after HiR treatment only, both treatments and after HiB treatment only. Shown sequences correspond to 12 nt located upstream and downstream of the 3′ss and 5′ss. Intronic and exonic sequences are indicated; in addition, exonic sequences are boxed. The 3′ss and 5′ss are marked by arrows. The identity of each exon is given at the beginning of each row. Whether each exon inclusion was affected by HiR or HiB treatment only, or by both, is denoted at the right side. ΔSkip indicates the change in the proportion of total transcript that has skipping of the indicated exon. 3′ss and 5′ss scores indicate the predicted strength of the splice sites as compared with the consensus sites. The last three columns indicate the sizes of the upstream intron, alternative exon and downstream intron. (**B**–**D**) Genomic views of RNA-Seq reads (upper panels) and RT–PCR results (lower panels) examining EIN events that are triggered by HiR only (**B**), by both HiR and HiB (**C**) and by HiB only (**D**) in GM03813 fibroblasts. For genomic views, treatment types are indicated at the left, and exon/intron arrangement at the bottom. Exons are depicted as blue boxes and introns as black lines. Red boxes indicate longer forms of exons that can be generated by usage of an alternative 5′ss or 3′ss. Exon names and sizes are given below and above each exon, respectively. Intron sizes are indicated below each intron. Transcription termination sites are indicated with a red octagon. Green arrows mark the location of treatment-affected exons. For representative gel images showing the splicing pattern of specific exons, labeling is the same as in Figure 1B.

Employing semi-quantitative RT–PCR followed by analysis of the amplified products in a gel-based assay, we performed independent validations of nine EIN events captured by RNA-Seq. We first analyzed EIN events for three coding exons: *MBNL1* exon 5, *TTC28* exon 20 and *EXOC1* exon 12 shown to be impacted by HiR but not by HiB (Figure 3B, lower panel). The MBNL1 protein plays an important role in RNA metabolism and, inclusion of *MBNL1* exon 5, which codes for a nuclear location signal, could be critical for general splicing regulation (82). Sequestration of MBNL1 in the nucleus by repeat-containing transcripts is associated with myotonic dystrophy (83). Both the 3′ss and 5′ss of *MBNL1* exon 5 are quite weak, unlike most targets of risdiplam (Figure 3A; Supplementary Figure S4B). Like most targets of risdiplam alone, the last position of *MBNL1* exon 5 is a G residue. The proteins coded by *TTC28* and *EXOC1* regulate the mitotic cell cycle and exocytosis, respectively. Aberrant expression of *TTC28* and *EXOC1* is associated with melanoma and platelet secretion defects, respectively (84,85). Both *TTC28* exon 20 and *EXOC1* exon 12 have a weak 3′ss and strong 5′ss (Figure 3A). The last position of *TTC28* exon 20 is a G, while the last position of *EXOC1* exon 12 is an A. Our PCR results confirmed the stimulatory effect of InR and HiR on inclusion of *MBNL1* exon 5, *TTC28* exon 20 and *EXOC1* exon 12 (Figure 3B, lower panel). The effect of risdiplam was particularly robust on splicing of shorter exons, for example *TTC28* exon 20 and *EXOC1* exon 12. Of note, the fact that risdiplam promoted inclusion of *MBNL1* exon 5 and *TTC28* exon 20 despite the absence of an A nucleotide at the last position of these exons suggests a different mechanism of risdiplam action from the one proposed for modulation of *SMN2* exon 7 splicing. As expected, none of the branaplam concentrations used had any significant effects on splicing of *MBNL1* exon 5 and *TTC28* exon 20 (Figure 3B).

We next validated the splicing of coding exons of *POMT2*, *STRN3* and *FOXM1* shown to be affected by both compounds according to our RNA-Seq analysis (Figure 3C). *POMT2*, *STRN3* and *FOXM1* code for *O*-mannosyltransferase, a signaling protein and a transcription activator, respectively. Mutations and/or aberrant splicing of all three genes are associated with pathogenic conditions, including muscular dystrophy and cancer (86–88). All three exons in question have a nearly identical 5′ss, with an A at the last exonic position and a canonical GUAAGU intronic sequence (Figure 3A). Their 3′ss varies in strength: *POMT2* exon 11B and *FOXM1* exon 9 have a relatively strong 3′ss, while that of *STRN3* exon 8 is weaker. *FOXM1* exon 9 contains a second, internal 5′ss (9S) that is also predicted to respond to risdiplam and branaplam treatment. This splice site shares many sequence characteristics with the others, namely an A at the last exonic position and a GUAAGU intronic sequence. Supporting the findings of RNA-Seq, the results of PCR showed an increase in inclusion of *POMT2* exon 11B in the presence of risdiplam and branaplam in a dose-dependent manner (Figure 3C). Of note, *POMT2* exon 11B is a previously unannotated exon, whose identity we confirmed by sequencing. While analysis of RNA-Seq reveals stimulation of *STRN3* exon 8 inclusion by HiR and HiB, no information could be deduced about co-skipping events involving *STRN3* exons 8 and 9 (Figure 3C, lower panel). PCR validations confirmed dose-dependent stimulation of *STRN3* exon 8 inclusion by risdiplam and branaplam treatments, with the effect of branaplam being stronger (Figure 3C, lower panel). Treatments with the compounds also resulted in an increase of *STRN3* exon 8 and 9 inclusions, which is indicated by the increase in the corresponding band intensity (Figure 3C, lower panel). RNA-Seq analysis supported the increase in usage of both 5′ss upon treatments with the compounds (Figure 3C, lower panel), although limitations of mapping could not accurately delineate the relative proportions of the long and short isoforms of *STRN3* (Figure 3C, upper panel). PCR validation confirmed the stimulatory effect of InR and HiR on inclusion of exon 8 and 9 of *STRN3* (Figure 3C, lower). As mentioned previously, *FOXM1* exon 9 can be included in two isoforms, short (9S) and long (9L), due to the alternative 5′ss usage. Branaplam caused inclusion of both isoforms in a dose-dependent manner, while risdiplam primarily induced usage of the downstream 5′ss to include the 9L isoform. In the case of HiB, we also captured inclusion of an unannotated downstream exon. We confirmed its identity by sequencing and termed it 9B (Figure 3C). Our findings provide new information about co-skipping events compared with earlier studies that analyzed the off-target effects of risdiplam and branaplam on splicing of exons 8 and 9 of *STRN3* and exon 9 of *FOXM1* by RNA-Seq or qPCR (38,48). Of note, a gel-based method remains the only reliable technique to accurately quantify co-skipping of two or more exons in a single lane.

We examined three coding exons that showed enhanced inclusion by HiB but not by HiR. These were *KDM6A* exon 28, *ANXA11* exon 16B and *SH3YL1* exon 11 (Figure 3D, upper panel). Proteins encoded by *KDM6A*, *ANXA11* and *SH3YL1* regulate demethylation, calcium-dependent phospholipid binding and phosphatidylinositol biosynthesis, respectively. Pathogenic conditions associated with aberrant expression of *KDM6A*, *ANXA11* and *SH3YL1* include pediatric cancer, ALS and diabetic nephropathy, respectively (89–91). All three exons had a somewhat weaker than average 5′ss, while the 3′ss of *KDM6A* exon 28 and that of *SH3YL1* exon 11 were quite strong. In contrast, *ANXA11* exon 16B had a weak 3′ss (Figure 3A). Both *KDM6A* exon 28 and *ANXA11* exon 16B ended with A residues, while the last position of *SH3YL1* was a G. Our PCR results confirmed stimulation of *KDM6A* exon 28 inclusion by InB and HiB (Figure 3D, lower panel). HiR also stimulated inclusion, but to a much lesser extent. In addition, InB and HiB triggered a significant retention of *KDM6A* intron 28. According to the RNA-Seq analysis, HiB had a complex effect on processing of the *ANXA11* exons 16L and 16B (Figure 3D, upper panel). The results of PCR confirmed the increase of *ANXA11* exon 16B inclusion by InB and HiB, while risdiplam did not change the splicing of this exon (Figure 3D, lower panel). Using PCR, we also confirmed that branaplam promoted inclusion of *SH3YL1* exon 11; this effect was dose dependent (Figure 3D).

### Analysis and validation of exon skipping events triggered by risdiplam and branaplam

We examined the sequence context of the splice sites for 30 affected exons whose skipping was increased by HiR and/or HiB (Supplementary Figure S5). As compared with the average 147 nt size of an internal exon, the median sizes of exons undergoing increased skipping triggered by HiR only, HiB only or both were relatively short, at 55, 104 and 80 nt, respectively. However, intronic sequences flanking these skipped exons did not show any size preference. In general, skipped exons tended to have a slightly weaker than average 3′ss. The average 3′ss strengths were 6.7, 6.7 and 6.3 for exons affected by HiR only, HiB only and both treatments, respectively. We noted an interesting trend for the 5′ss of exons undergoing a compound-induced increase in skipping. Although the 5′ss of exons affected by HiR and HiB treatment only were relatively strong on average (average scores of 8.0 and 9.1, respectively), exons whose skipping was increased by both HiR and HiB tended to have a very weak 5′ss, with an average score of 4.2 (Supplementary Figure S5). Unlike exons with increased inclusion after treatment with these compounds, there was no preference for an A residue at the last exonic position.

**Figure 4.**
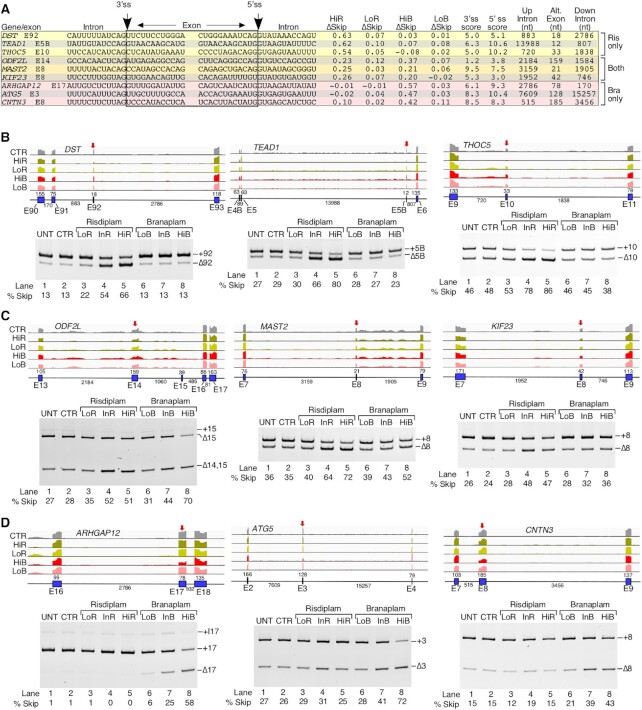
Risdiplam and branaplam promote off-target exon skipping. (**A**) Table summarizing splicing-relevant information regarding top exons, skipping of which was increased by HiR treatment only, by both HiR and HiB treatments and by HiB treatment only. Table contents are similar to those in Figure 3A. (**B**–**D**) Genomic views of RNA-Seq reads (upper panels) and RT–PCR results (lower panels) examining ESK events that are triggered by HiR only (**B**), both HiR and HiB (**C**) and HiB only (**D**) in GM03813 fibroblasts. For genomic views, red arrows indicate the location of the affected exon. Other labeling and coloring are the same as in Figure 3B–D.

We employed PCR to validate representative ESK events for coding exons of three genes: *DST*, *TEAD1* and *THOC5*. Their splicing was impacted by risdiplam but not by branaplam. Proteins coded by *DST*, *TEAD1* and *THOC5* regulate microtubule organization, transcription and RNA metabolism, respectively. Aberrant expression and/or splicing of *DST* (also known as *BPAG1*), *TEAD1* and *THOC5* are associated with neurological disorders and cancer (92–94). *DST* exon 92 has a relatively weak 3′ss and 5′ss, while *TEAD1* exon 5B and *THOC5* exon 10 have a weak 3′ss, but a strong 5′ss. Like most candidates undergoing ESK events, all three have a G at the last exonic position. Supporting the findings of RNA-Seq, PCR results showed enhanced skipping of *DST* exon 92, *TEAD1* exon 5B and *THOC5* exon 10 in the presence of InR and HiR (Figure 4B, lower panel). Although not initially apparent from RNA-Seq analysis, the results of PCR also revealed a small but noticeable stimulatory effect of HiB on splicing of *THOC5* exon 10 (Figure 4B). We confirmed ESK events of three coding exons triggered by both HiR and HiB: *ODF2L* exon 14, *MAST2* exon 8 and *KIF23* exon 8 (Figure 4C, upper panel). Proteins coded by *ODF2L*, *MAST2* and *KIF23* regulate cilium assembly, peptidyl-serine phosphorylation and transport of organelles during cell division, respectively. Pathogenic conditions associated with aberrant expression of *ODF2L*, *MAST2* and *KIF23* include ciliopathy, liver cancer and chronic myeloid leukemia, respectively (95–97). Both *ODF2L* exon 14 and *KIF23* exon 8 have a weak 3′ss and 5′ss, while *MAST2* has a strong 3′ss and a slightly weaker than average 5′ss. The last exonic position was G in the case of *ODF2L* and *MAST2*, while *KIF23* exon 8 had a U residue. The PCR results showed that InR and HiR produced a nearly equal effect on splicing of *ODF2L* exon 14, suggesting a lower risdiplam concentration threshold for maximum inhibitory effect that was limited to ∼50% skipping of *ODF2L* exon 14 (Figure 4C, lower panel). At the same time, an increase in branaplam concentrations resulted in a progressive increase in skipping of *ODF2L* exon 14 (Figure 4C). PCR results also confirmed an increase in skipping of *MAST2* exon 8 with increasing concentrations of the compounds, with the effect of risdiplam being stronger (Figure 4C). Our PCR results also verified that skipping of *KIF23* exon 8 increased at InR, HiR, InB and HiB, with risdiplam eliciting a stronger response (Figure 4C). We validated three ESK events associated with coding exons affected by HiB only. These were *ARHGAP12* exon 17, *ATG5* exon 3 and *CNTN3* exon 8. Proteins coded by *ARHGAP12*, *ATG5* and *CNTN3* regulate GTPase activation, autophagy and cell adhesion, respectively. Aberrant expression of *ARHGAP12*, *ATG5* and *CNTN3* is associated with different types of carcinomas (98–100). Splice sites of all three exons were relatively strong, with only the 3′ss of *ARHGAP12* below average strength (Figure 4A). All three had a G residue at the last exonic position. Supporting the findings of RNA-Seq, the results of PCR confirmed a significant increase in skipping of these exons in the presence of InB and HiB (Figure 4D). As expected, PCR results did not show any significant effect of risdiplam on splicing of these exons.

### Analysis and validation of alternative splice site usage triggered by risdiplam and branaplam

We examined the sequence context of the top 15 A5S events affected by HiR and/or HiB (Supplementary Figure S6A). For A5S events affected by risdiplam only, all of the 5′ss with increased usage had lower scores than the 5′ss with decreased usage, suggesting that risdiplam may be acting by directly strengthening a weak 5′ss. We only observed a single 5′ss with an A residue at the last position of the exon, and its usage was decreased by risdiplam treatment. There was less consistency among sequences when 5′ss usage was influenced by both compounds. In particular, in three cases, the two alternative splice sites were of similar strength, whereas in the other two the splice site with increased usage after treatment was the stronger of the two. Two of the 5′ss with increased usage had an A residue at the last exonic position and three had the canonical G residue. Among the top five A5S events affected by branaplam only, there was no clear trend in regard to the relative strength of the alternative 5′ss, but all five 5′ss whose usage was promoted by HiB had A residues at the last exonic position.

**Figure 5.**
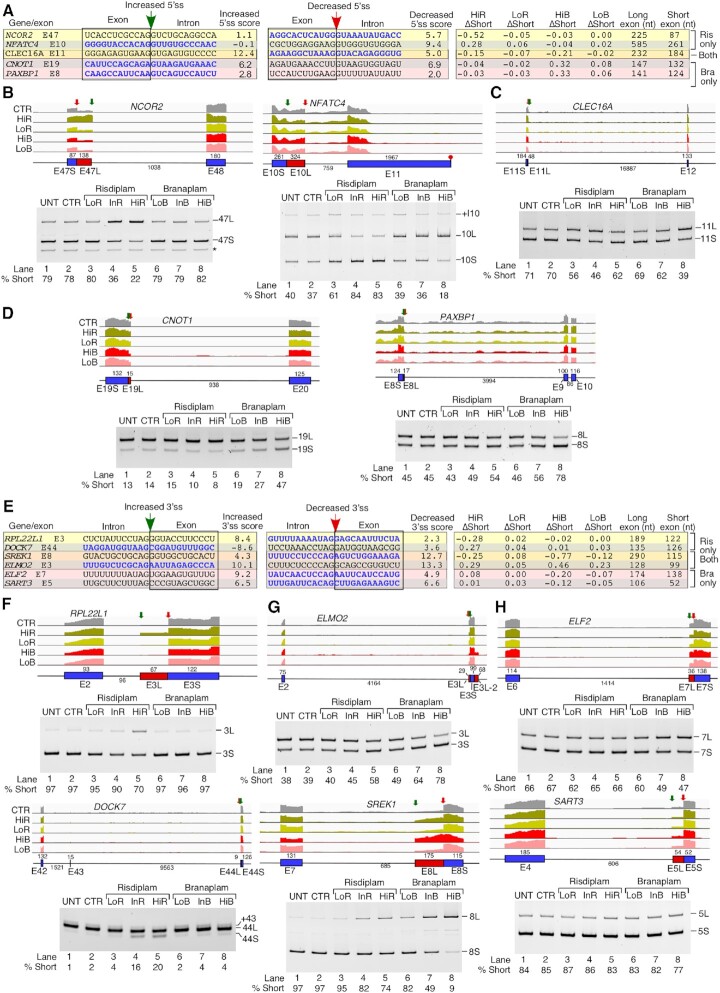
Risdiplam and branaplam affect usage of alternative 5′ss and 3′ss. (**A**) Table summarizing splicing-relevant information regarding the top candidate exons with altered 5′ss usage after HiR treatment only, both treatments or after HiB treatment only. Sequences represent 12 bases upstream and downstream of the two 5′ss with increased (left) and decreased usage (right) after small compound treatment. Intronic and exonic sequences are indicated; exonic sequences are boxed. The 5′ss score of each splice site is indicated to the right of each sequence. ΔShort indicates the change in proportion of total transcript with the shorter form of the exon due to the usage of the upstream 5′ss. The sizes of the long and short forms of the exon in question are indicated. (**B**–**D**) Genomic views of RNA-Seq reads (upper panels) and RT–PCR results (lower panels) examining A5S events that are triggered by HiR alone (**B**), both HiR and HiB (**C**) and HiB alone (**D**) in GM03813 fibroblasts. For genomic views, green arrows indicate the location of the 5′ss with increased usage, red arrows the 5′ss with decreased usage. ‘L’ indicates a longer isoform of an exon due to usage of the downstream 5′ss; ‘S’, a shorter one due to usage of the upstream 5′ss. An asterisk marks a non-specific PCR product. Other labeling and coloring are the same as in Figure 3B–D. (**E**) Table summarizing splicing-relevant information regarding the top candidate exons with altered 3′ss usage after HiR treatment only, both treatments or after HiB treatment only. Table content is similar to (A), except that the 3′ss were analyzed. (**F**–**H**) Genomic views of RNA-Seq reads (upper panels) and RT–PCR results (lower panels) examining A3S events that are triggered by HiR only (**F**), both HiR and HiB (**G**) and HiB only (**H**). For genomic views, in the case of >2 alternative 3′ss, the intermediate sized region is indicated with a pink box. Green arrows indicate the location of the 3′ss with increased usage, red arrows the 3′ss with decreased usage. ‘L’ indicates a longer isoform of an exon due to usage of the upstream 3′ss; ‘S’, a shorter one due to usage of the downstream 3′ss. Other coloring and labeling are the same as in (B–D).

We selected representative coding exons of five genes, namely *NCOR2*, *NFATC4*, *CLEC16A*, *CNOT1* and *PAXBP1*, to validate by PCR the A5S events identified by RNA-Seq (Figure 5A–D). *NCOR2*, *NFATC4* and *PAXBP1* code for transcription-associated factors and their dysregulations are linked to Crohn's disease, carcinogenesis and myopathic hypotonia, respectively (101–103). *CLEC16A* and *CNOT1* code for autophagy- and RNA turnover-related factors, and their dysregulations are associated with Parkinson's disease and impaired neurological development, respectively (104,105). *NCOR2* exon 47 has two competing 5′ss that are 138 nt away from each other. Both splice sites are weak, and neither harbors an A residue upstream of the GU (Figure 5A). Consistent with analysis of RNA-Seq, the results of PCR showed increased usage of the downstream 5′ss upon treatments with InR and HiR (Figure 5B, lower panel). As expected, the results of PCR did not capture any noticeable effect of branaplam on the 5′ss usage of *NCOR2* exon 47. In the case of *NFATC4*, two competing 5′ss of exon 10 are separated from each other by 324 nt. Usage of the downstream 5′ss, which is strong, is reduced in favor of usage of the upstream weak 5′ss (Figure 5A, B). In addition to alternative usage of the 5′ss of exon 10, analysis of RNA-Seq revealed retention of intron 10 (Figure 5B, upper panel). This suggests that risdiplam may function by weakening the downstream 5′ss, rather than strengthening the upstream 5′ss. Supporting the findings of RNA-Seq, PCR results confirmed increased usage of the upstream 5′ss caused by risdiplam in a dose-dependent manner (Figure 5B, lower panel). Although not revealed by RNA-Seq, the results of PCR also showed an increase in usage of the downstream 5′ss of exon 10 of *NFATC4* in the presence of branaplam (Figure 5B, lower panel). Here again, selection of the 5′ss in response to treatments was not determined by the identity of the nucleotide at the last exonic position, as it is a G residue in both 5′ss. PCR did not reveal any appreciable change in the retention of intron 10 of *NFATC4* by any treatment, although these results should be interpreted with caution as longer intron-containing transcripts are likely to be poorly amplified. The competing 5′ss of *CLEC16A* exon 11 are separated from each other by 48 nt. The strong downstream 5′ss is increased after treatment compared with the weaker upstream 5′ss. Both splice sites have G residues at the last exonic position, again confirming that alternative 5′ss usage is not always directly linked to the last exonic position. Confirming the findings of RNA-Seq, the results of PCR showed a risdiplam-induced increase in usage of the downstream 5′ss, although the effect was not strictly concentration-dependent (Figure 5C, lower panel). InB and HiB also increased usage of the downstream 5′ss of *CLEC16A* exon 11, with the largest effect observed at HiB (Figure 5C, lower panel). *CNOT1* exon 19 has two alternative 5′ss located 15 nt apart. Both splice sites are somewhat weaker than average and, unlike targets of risdiplam and both drugs, the increased upstream 5′ss incorporates an A residue at the last exonic position (Figure 5A). RNA-Seq analysis showed a HiB-induced increase in usage of the upstream 5′ss site (Figure 5D, upper panel). PCR results validated this increase for both InB and HiB, with the effect being more pronounced in the case of HiB (Figure 5D, lower panel). *PAXBP1* exon 8 has two 5′ss that are 17 nt apart; their usage in control samples is comparable (Figure 5D, upper panel). Both splice sites are quite weak. Like *CNOT1* exon 19, the upstream 5′ss is strengthened in the presence of branaplam, and contains an A at the last exonic position (Figure 5A). Consistent with the findings of RNA-Seq, the results of PCR showed enhanced usage of the upstream 5′ss caused by InB and HiB, with the strongest effect observed at HiB (Figure 5D, lower panel).

We analyzed the sequence context of the top 15 A3S events that were affected by HiR and/or HiB according to our RNA-Seq analysis (Supplementary Figure S6B). We could not discern any common sequence feature as a determinant of A3S events, suggesting that the mode of compound action may be complex and/or indirect. We selected representative coding exons of six genes, namely *RPL22L1*, *DOCK7*, *ELMO2*, *SREK1*, *ELF2* and *SART3*, to validate A3S events identified by RNA-Seq (Figure 5E). *RPL22L1*, *SREK1* and *SART3* code for RNA-binding proteins, and their aberrant expression is linked to carcinogenesis (106–108). Dysregulation of *ELF2*, a transcription factor-coding gene, is also associated with carcinogenesis (109). Aberrant expression of *DOCK7* that codes for a guanine nucleotide exchange factor is associated with epileptic encephalopathy (110). *ELMO2* codes for a phagocytosis-associated protein, and homozygous mutation in *ELMO2* has been linked to Ramon syndrome (111). *RPL22L1* exon 3 has two alternative 3′ss separated from each other by 67 nt. The upstream 3′ss, usage of which is increased in risdiplam treatment, is stronger than average, while the canonical downstream 3′ss is quite weak (Figure 5E). PCR validations confirmed an increase in usage of the upstream 3′ss of *RPL22L1* exon 3 in the presence of InR and HiR, with the effect being most pronounced in the case of HiR (Figure 5F, upper panel). PCR results also confirmed an increase in usage of the downstream 3′ss of *DOCK7* exon 44 by InR and HiR (Figure 5F, lower panel). The downstream 3′ss is weak, so it is not surprising that even after HiR treatment its usage only reaches 20% (Figure 5E, F). *ELMO2* exon 3 has two competing 5′ss and two competing 3′ss (Figure 5G). Both 3′ss are strong. Consistent with the findings of RNA-Seq, PCR results showed increased inclusion of the shorter exon due to enhanced usage of the downstream 3′ss caused by both compounds, with the strongest effect observed at HiB (Figure 5G, upper panel). *SREK1* exon 8 has two competing 3′ss that are 175 nt apart (Figure 5G). Supporting the findings of RNA-Seq, both compounds increased usage of the upstream weak 3′ss over the strong downstream 3′ss in a dose-dependent manner, with the strongest effect produced by HiB (Figure 5G, lower panel). *ELF2* exon 7 has two alternative 3′ss separated from each other by 36 nt (Figure 5H, upper panel). PCR validation confirmed branaplam-induced usage of the upstream strong 3′ss over the downstream weaker 3′ss in a dose-dependent manner (Figure 5H, upper panel). *SART3* exon 5 has two alternative 3′ss that are 54 nt apart (Figure 5H, lower panel). Both are somewhat weaker than average. Results of PCR validated HiB-induced inclusion of the longer form of exon 5 of *SART3*, confirming that usage of the upstream 3′ss was stimulated by HiB (Figure 5H, lower panel).

### Analysis and validation of differential intron retention triggered by risdiplam and branaplam

**Figure 6.**
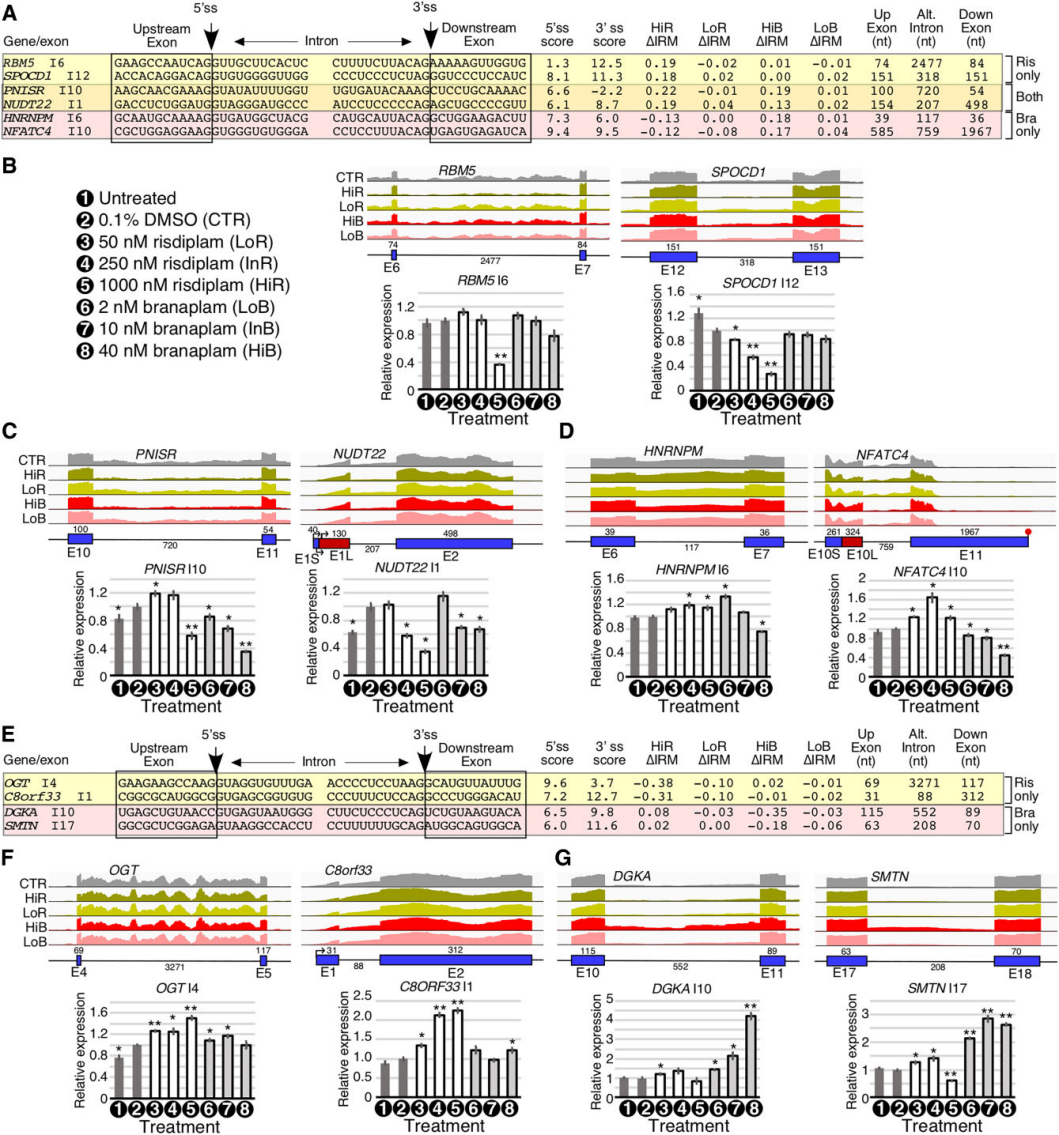
Risdiplam and branaplam trigger changes in intron retention. (**A**) Table summarizing splicing-relevant information for the top candidate introns with improved removal after HiR only, both treatments or after HiB only. Shown sequences correspond to 12 nt upstream and downstream of the 5′ss and 3′ss that flank the affected intron. The 5′ss and 3′ss scores are given. ΔIRM indicates the change in intron removal after treatment (positive numbers indicate improved removal, and negative numbers indicate retention). The sizes of the upstream exon, affected intron and downstream exon are given. (**B**–**D**) Genomic views of RNA-Seq reads (upper panels) and qPCR results (lower panels) examining IRM events affected by HiR only (**B**), both HiR and HiB (**C**) or HiB only (**D**) in GM03813 fibroblasts. For genomic views, coloring and labeling are the same as in Figure 3B–D. For qPCR, the gene symbol and measured intron are indicated above each graph. Treatments are indicated under the *x*-axis. Treatment symbols are the same as in Figure 2 and are described in the leftmost panel of (**B**). The *y*-axis represents the relative expression relative to DMSO-treated (CTR) cells of intron-containing transcripts corrected by total expression of the gene in question. Error bars indicate the SEM; *n*= 3; *P <0.05; **P <0.01. (**E**) Table summarizing splicing-relevant information for the top candidate introns with increased retention after HiR only or after HiB only. Table contents are similar to (A). (**F**, **G**) Genomic views of RNA-Seq reads (upper panels) and qPCR measuring intron retention (lower panels) of IRT events affected by HiR alone (**F**) or HiB alone (**G**). Coloring, labeling and statistics are similar to (B–D).

We examined the characteristics of introns with IRM events triggered by risdiplam and/or branaplam. There were no clear trends among the splice sites of IRM introns regarding their sequences or strengths (Supplementary Figure S7A). Somewhat surprisingly, only one IRM intron identified by RNA-Seq is flanked by a 5′ss with an A at the last exonic position. The majority of affected introns were relatively short (<1 kb), although this is not unexpected since most longer retained introns are likely to be degraded by RNA surveillance mechanisms. Using qPCR, we validated the IRM events of six representative introns identified by RNA-Seq (Figure 6A–D). In order to avoid ambiguities of expression, we normalized each intron retention value using a constitutively spliced region of the same gene. We also performed standard qPCR normalization against a housekeeping gene and compared changes in total transcript levels with changes in intron-retained transcripts, along with untreated control and intermediate concentrations of the compounds (Supplementary Figure S7).

Genes used for validation of IRM events were *RBM5*, *SPOCD1*, *PNISR*, *NUDT22*, *HNRNPM* and *NFATC4*. *RBM5*, *PNISR* (also known as *SFRS18*) and *HNRNPM* code for RNA-binding proteins, and dysregulations of these genes are associated with carcinogenesis, psoriasis and cognitive deficits, respectively (112–114). *SPOCD1* and *NFATC4* code for a transcription factor and a transcription-associated factor, respectively. Aberrant expression of these genes is linked to glioma and oncogenesis (102,115). *NUDT22* codes for a protein with DP-sugar diphosphatase activity and metal ion binding activity; however, the pathological significance of NUDT22 has not yet been established (116). The results of qPCR confirmed a significant reduction in retention of *RBM5* intron 6 by HiR (Figure 6B). Of note, this is coupled with an increase in *RBM5* total transcript levels (Supplementary Figure S7D). *RBM5* exon 6 is longer than most targets of IRM events at ∼2.5 kb, and its 5′ss is quite weak (Figure 6A; Supplementary Figure S7A). Validating the findings of RNA-Seq, the results of qPCR showed concentration-dependent reduction in *SPOCD1* intron 12 retention triggered by risdiplam (Figure 6B). The 5′ss of *SPOCD1* intron 12 is of average strength, while its 3′ss is strong. *PNISR* intron 10 has a somewhat weak 5′ss and a very weak 3′ss. Typical of other IRM targets, *PNISR* intron 10 is >1 kb in length (Figure 6A). qPCR results confirmed a decrease in *PNISR* intron 10 retention by HiR and HiB (Figure 6C). InB also reduced *PNISR* intron 10 retention, but to a lesser degree. Interestingly, LoR and InR produced an increase in *PNISR* intron 10 retention. The opposite effects between high and low concentrations of risdiplam could be due to secondary effects that might come into play at high risdiplam concentration. *NUDT22* intron 1 does not have any exceptional features, except for its small size (Figure 6A). In line with the findings of RNA-Seq, the results of qPCR showed reduced retention of *NUDT22* intron 1 by InR, HiR, InB and HiB (Figure 6C). The results of qPCR also validated HiB-induced reduction in *HNRNPM* intron 6 retention. However, we observed the opposite effect on retention of this intron in the presence of InR, HiR and LoB (Figure 6D). These differences could be attributed to secondary effects caused by a high concentration of branaplam. Supporting the findings of RNA-Seq, qPCR validations confirmed the dose-dependent reduction in the retention of *NFATC4* intron 10 by branaplam (Figure 6D). In contrast, qPCR results showed an increase in *NFATC4* intron 10 retention by risdiplam in a dose-dependent manner (Figure 6D). Of note, *NFATC4* intron 10 uses two alternative 5′ss, usage of which is regulated in contrasting manners by risdiplam and branaplam (Figure 5). Since both intron retention and 5′ss usage are regulated in opposite manners by the two compounds, we hypothesize that the two events are linked.

Similar to IRM, IRT events caused by risdiplam and/or branaplam involved mostly short introns, with only two exceptions of introns being longer than 1 kb (Supplementary Figure S7B). In addition, introns whose retention was increased by branaplam tended to have a somewhat weak 5′ss, but other than that we observed no clear trends regarding sequence composition or splice site strengths for the affected introns. There was little overlap between IRT events triggered by risdiplam and branaplam treatments (Figure 1J); those events that we identified were not strongly affected compared with those that were triggered by risdiplam only or branaplam only (Supplementary Figure S7B). Using qPCR, we validated the IRT events for *OGT*, *C8orf33*, *DGKA* and *SMTN* genes that code for glycosyltransferase, an unknown protein, diacylglycerol kinase and a structural protein, respectively (Figure 6E). Expression of *OGT*, *C8orf33*, *DGKA* and *SMTN* has been correlated with cardiovascular disease, hepatocellular carcinoma, acute myeloid leukemia and hypertension, respectively (117–120). *OGT* intron 4 stands out among other IRT events due to its relatively long size and high level of baseline retention (Figure 6E, F). It has a strong 5′ss, but its 3′ss is rather weak. The results of qPCR confirmed the increase in *OGT* intron 4 retention by risdiplam at all concentrations, with the effect being most pronounced at HiR (Figure 6F, lower panel). LoB and InB also caused a small but significant increase in *OGT* intron 4 retention. However, HiB showed no significant effect on the splicing of *OGT* intron 4. The concentration-dependent contradictory effects could be due to multiple factors including secondary effects that are likely to become more prominent at the high concentration of branaplam. *C8orf33* intron 1 is quite short at 88 nt, with a slightly weaker than average 5′ss and a strong 3′ss (Figure 6E). Consistent with the results of RNA-Seq, the results of qPCR showed an increase in *C8orf33* intron 1 retention by risdiplam at all concentrations, with the effect already plateauing at InR (Figure 6F, right panel). qPCR results also confirmed the HiB-induced retention of *C8orf33* intron 1, although the effect was less than that observed with HiR and InR (Figure 6F). *DGKA* intron 10 has a somewhat weak 5′ss paired with a strong 3′ss, and its 552 nt size is typical of other IRT introns (Figure 5E). In agreement with the findings of RNA-Seq, qPCR results showed an increase in retention of *DGKA* intron 10 by branaplam in a concentration-dependent manner (Figure 6G, left panel). Like *DGKA* intron 10, *SMTN* intron 17 has a weaker than average 5′ss paired with a strong 3′ss and a small size. The results of qPCR confirmed the increase in *SMTN* intron 17 retention by branaplam at all concentrations used, with the effect already plateauing at the intermediate concentration of branaplam (Figure 6G, right panel). LoR and InR also showed a small but significant increase in retention of this intron (Figure 6G). Interestingly, HiR caused an opposite effect as it promoted *SMTN* intron 17 removal (Figure 6G), driven mostly by an increase in *SMTN* total expression without a concomitant change in the intron-retained product (Supplementary Figure S7H).

### Mechanism of risdiplam and branaplam action captured by minigenes

To uncover if the effects of the small molecules were directly linked to motifs located within the affected exons and their flanking intronic sequences, we employed hybrid exon trapping cassettes using an *SMN2* minigene (p*SMN2*ΔI6) as the ‘backbone’. We generated 11 hybrid minigenes by replacing *SMN2* exon 7 and its flanking intronic sequences with an exon of interest and its flanking intronic sequences (Supplementary Figure S8). These minigenes harbored *EXOC1* exon 12, *TTC28* exon 20, *TMEM50B* exon 5B, *SH3YL1* exon 11, *POMT2* exon 11B, *STRN3* exon 8, *FOXM1* exon 9, *SLC25A17* exon 3, *MADD* exon 21, *ARHGAP12* exon 17 and *ATG5* exon 3. Of note, the off-target effects of risdiplam and/or branaplam on splicing of *STRN3* exon 8, *FOXM1* exon 9, *SLC25A17* exon 3, *MADD* exon 21 and *ATG5* exon 3 have been also validated independently (38,48). Splicing of exonic sequences examined in this study provided diverse structural context (Supplementary Figures S9–S12). To determine a potential overlapping mechanism of splicing modulation by risdiplam and branaplam, we created mutant minigenes in which the nucleotide at the last exonic position was substituted with a C residue. For a few minigenes, we also tested the effect of other mutations, exonic and intronic, on the ability of the small molecules to affect splicing. To ensure efficient transfection, we performed all hybrid minigene-based experiments in HeLa cells. Of note, the effects of HiR and HiB on splicing of the endogenous genes in HeLa cells were largely similar to those observed in GM03813 fibroblasts (Supplementary Figure S8). Due to the facts that minigene expression was driven by a strong constitutive promoter, and the minigene context was heterologous, we did observe some differences in the level of exon inclusion/skipping between the minigenes and the endogenous genes. Importantly, the minigenes responded to the treatments with the small molecules, allowing interrogations into the mechanism of action of the compounds.

**Figure 7.**
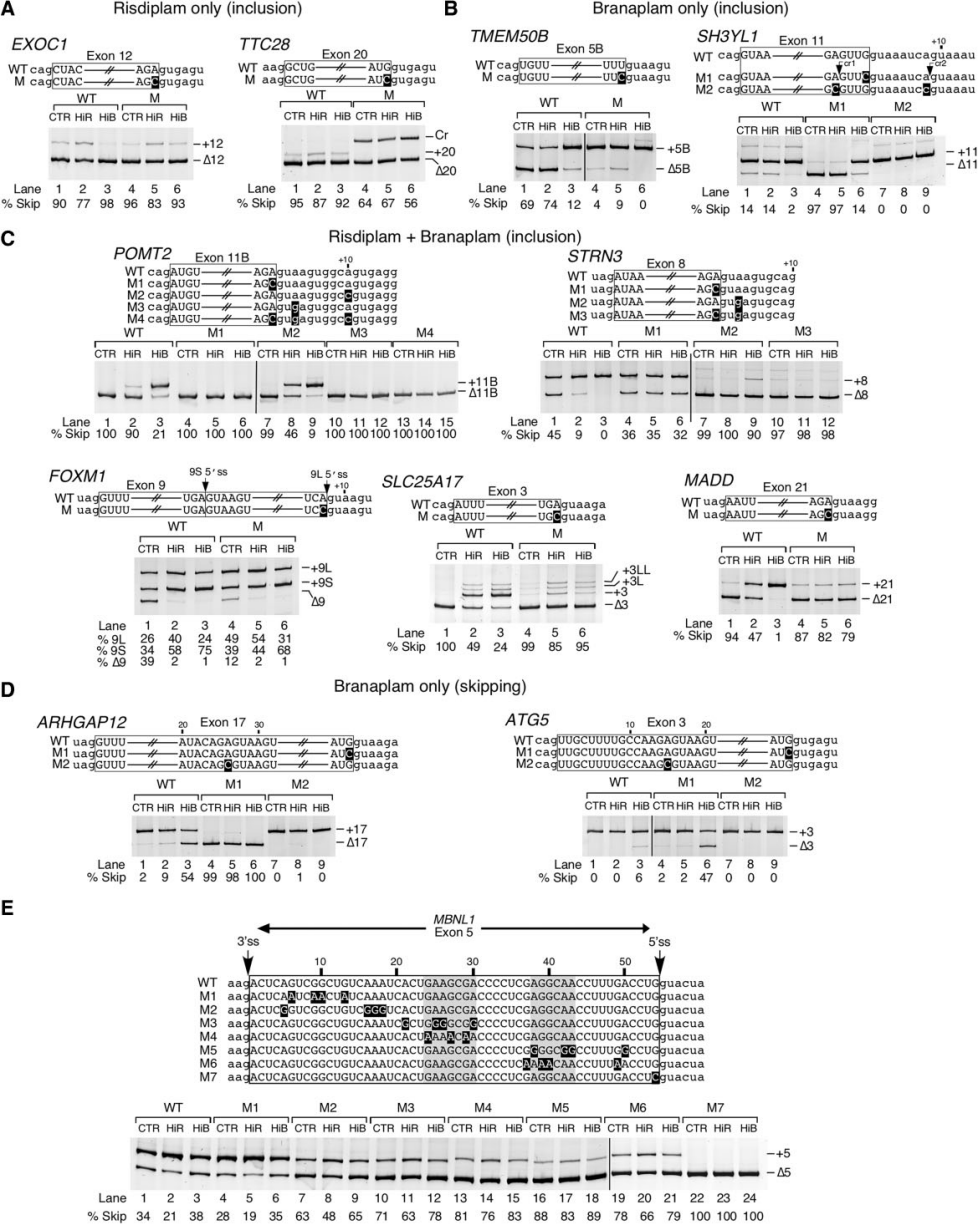
Usage of minigenes provides insights into the mechanism of action of risdiplam and branaplam on splicing of off-target exons. (**A**) Enhanced inclusion of exons by HiR in transcripts generated from hybrid minigenes in HeLa cells. Diagrammatic representation of affected exons is given in the upper panel with the first four and last three exonic nucleotides shown in uppercase letters and immediate upstream and downstream intronic sequences in lowercase letters. Exon numbers and names of their corresponding genes are indicated at the top of the diagrams. Minigene names are given on the left. Mutated nucleotides are highlighted in white on a black background. Representative gel images showing the splicing pattern of hybrid minigenes in HeLa cells are given in the lower panel. Treatments and minigene names are presented at the top of each gel image. ‘Δ’ indicates exon skipping; ‘+’, exon inclusion. Abbreviations: Cr, cryptic 5′ss; WT, wild type; M, mutant. Other abbreviations are the same as in Figure 1B. (**B**) Enhanced inclusion of exons by HiB in transcripts generated from hybrid minigenes. Labeling is the same as in (A). In addition, activated cryptic 5′ss are marked by arrows. (**C**) Enhanced inclusion of exons by HiR and HiB in transcripts generated from hybrid minigenes. Labeling is the same as in (A). (**D**) Enhanced skipping of exons by HiB in transcripts generated from hybrid minigenes. Labeling is the same as in (A). (**E**) Enhanced inclusion of *MBNL1* exon 5 by HiR in transcripts generated from a minigene encompassing the entire genomic sequence of *MBNL1* from exons 4 to 6. The entire exon 5 and its flanking intronic sequences for *MBNL1* minigene mutants are shown in the upper panel. The splice sites are indicated by arrows. Mutated nucleotides are shown in white on a black background. Gray shaded boxes indicate A/G-rich motifs, mutation of which greatly reduces the ability of risdiplam to stimulate exon 5 inclusion. A representative gel image of *MBNL1* minigenes splicing in GM03813 patient fibroblasts is given in the lower panel. Labeling of the gel is the same as in (A).

We observed HiR-induced enhanced inclusion of *EXOC1* exon 12 and *TTC28* exon 20 in transcripts generated from the wild-type minigenes (Figure 7A). Supporting the findings for *SMN2* exon 7, an A-to-C substitution at the last position of *EXOC1* exon 12 reduced the ability of risdiplam to promote inclusion of the exon, although this substitution itself had an inhibitory effect on splicing (Figure 7A). In the case of the *TTC28* minigene, risdiplam promoted wild-type exon 20 inclusion despite the nucleotide at the last position of this exon being G (Figure 7A). Changing G to C abolished exon 20 inclusion, with and without risdiplam. Interestingly, this G-to-C substitution also activated a downstream cryptic 5′ss (Cr), usage of which was somewhat promoted by branaplam (Figure 7A). We observed a HiB-linked increase in inclusion of *TMEM50B* exon 5B and *SH3YL1* exon 11 in transcripts generated from the minigenes (Figure 7B). In the case of *TMEM50B* exon 5B, a U-to-C substitution at the last exonic position resulted in increased exon inclusion. Importantly, the ability of branaplam to promote inclusion of the mutated *TMEM50B* exon 5B was retained (Figure 7B). These results supported that the presence of a specific nucleotide at the last exonic position is not a critical factor for the branaplam effect on *TMEM50B* exon 5B splicing. Similar results were obtained for *SH3YL1* exon 11. Interestingly, a G-to-C substitution at the last position of *SH3YL1* exon 11 caused predominant skipping of exon 11, yet HiB was able to effectively restore inclusion of the mutated exon; however, one of the two cryptic 5′ss was used in this case (Figure 7B). *The SH3YL1* sequence contains two AGU motifs near the 5′ss of exon 11 which could serve as targets for branaplam. Mutation of the A residue within both motifs to C (mutant M2) fully restored *SH3YL1* exon 11 (Figure 7B). While not useful for assessing the mechanism of the branaplam effect on splicing, *SH3YL1* mutant M2 underscored the role of residues outside of the 5′ss in determining the outcome of exon 11 splicing. It is possible that the interaction of branaplam with regions away from the 5′ss may have a similar effect to substitutions used in *SH3YL1* mutant M2.

Using minigenes, we were able to capture the stimulatory effect of both HiR and HiB on splicing of *POMT2* exon 11B, *STRN3* exon 8, *FOXM1* exon 9, *SLC25A17* exon 3 and *MADD* exon 21 (Figure 7C). In most instances, branaplam produced a stronger stimulatory effect than risdiplam, suggesting differences in the mechanisms of their action. Yet, there were many similarities between risdiplam and branaplam with respect to how exonic and intronic mutations impacted the ability of these small molecules to influence splicing. In the case of *POMT2*, an A-to-C substitution at the last position of exon 11B resulted in complete loss of the ability of HiR and HiB to promote exon 11B inclusion (Figure 7C). Interestingly, another single A-to-C substitution introduced at the 10th position of *POMT2* intron 11B (mutant M2) actually increased the ability of both compounds to promote exon 11B inclusion (Figure 7C). These results underscored the critical role of a few intronic nucleotides immediately downstream of the 5′ss in the effect of both compounds on splicing. We noted that exons targeted by both compounds tended to have an A residue at the third intronic position of the 5′ss (Figure 3A; Supplementary Figure S4). Mutation of this third position to G promoted skipping of *POMT2* exon 11B and suppressed the ability of the compounds to promote exon 11B inclusion, as did a mutant harboring all three mutations (Figure 7C).

In the case of *STRN3* exon 8, an A-to-C substitution at the last exonic position suppressed the ability of HiR and HiB to increase inclusion of this exon. An A-to-G mutation at the third position of intron 8 of *STRN3* triggered near total skipping of *STRN3* exon 8 and severely impacted the ability of the compounds to promote exon inclusion (Figure 7C). Interestingly, this intronic mutation had different consequences for the risdiplam and branaplam treatments, as only HiB but not HiR produced a small but noticeable increase in *STRN3* exon 8 inclusion (Figure 7C, mutant M2). We observed somewhat complex results for the impacts of HiR and HiB on splicing of *FOXM1* exon 9 that has an additional 5′ss within exon 9; usage of this splice site generates a shorter exon 9 isoform (exon 9S). While HiR promoted inclusion of both short and long isoforms of exon 9 by suppressing the skipping of exon 9, HiB preferentially promoted inclusion of exon 9S (Figure 7C). An A-to-C substitution at the last position of the long exon 9 reduced skipping of exon 9 in favor of the inclusion of the long isoform of exon 9 (exon 9L). Interestingly, HiR promoted inclusion of both 9S and 9L in the mutated exon 9. In contrast, HiB preferentially promoted inclusion of *FOXM1* exon 9S in the presence of the substitution (Figure 7C). These results provided a clear example in which the role of the last exonic position turned out to be more critical for the action of branaplam than of risdiplam. HiR and HiB promoted inclusion of multiple isoforms of *SLC25A17* exon 3. An A-to-C substitution at the last position of *SLC25A17* exon 3 substantially reduced, but did not completely abrogate, the stimulatory effect of HiR and HiB on splicing of this exon. Similar results were obtained in the case of an A-to-C mutation at the last position of *MADD* exon 21.

We analyzed two skipping events induced by HiB only: splicing of *ARHGAP12* exon 17 and *ATG5* exon 3. A G-to-C substitution at the last position of *ARHGAP12* exon 17 triggered almost total skipping of this exon (Figure 7D). Therefore, we were unable to capture the inhibitory effect of HiB on this mutant. We noted the presence of a 5′ss-like sequence early in exon 17 of *ARHGAP12* that has an A residue upstream of the GU dinucleotide. We hypothesized that this could serve as a target for branaplam to induce exon skipping. Introducing an A-to-C substitution at this position did not have an impact on splicing in the absence of branaplam. Yet, this exonic mutation fully suppressed the inhibitory effect of HiB on splicing of *ARHGAP12* exon 7 (Figure 7D). In the case of *ATG5* exon 3, a G-to-C substitution at the last exonic position of exon had a limited effect on exon 3 splicing. However, this mutation enhanced the inhibitory effect of HiB on its splicing (Figure 7D). An additional A-to-C substitution in exon 3 of *ATG5* at a 5′ss-like sequence similar to the one in *ARHGAP12* exon 17 did not have an impact on splicing in the control condition. Yet, this exonic mutation fully suppressed the inhibitory effect of HiR on splicing of *ATG5* exon 3 (Figure 7D).

Inclusion of *MBNL1* exon 5 is promoted by risdiplam but not branaplam in both GM03813 fibroblasts and HeLa cells (Figure 3; Supplementary Figure S8). Similar to *SMN2* exon 7, *MBNL1* exon 5 is 54 nt long. However, unlike *SMN2* exon 7 that has an A residue at the last exonic position, *MBNL1* exon 5 has a G residue at this position. Hence, *MBNL1* exon 5 could provide a useful model for uncovering the role of critical exonic positions/motifs in risdiplam-induced splicing changes without the dominating effect of the last exonic position in the context of a 54 nt long exon. The initial *MBNL1* hybrid minigene showed an aberrant pattern of splicing. Hence, we generated an *MBNL1* minigene encompassing the entire *MBNL1* genomic sequence from exon 4 through exon 6. In HeLa cells, the splicing pattern of this newly created minigene showed nearly 100% inclusion of exon 5, making the minigene useless for studying the stimulatory effect of HiR. On the other hand, in GM03813 fibroblasts, *MBNL1* exon 5 skipping in transcripts expressed from the minigene constituted 30–40%. Therefore, to perform our *MBNL1* minigene-related studies, we used GM03813 cells. As expected, HiR but not HiB increased inclusion of *MBNL1* exon 5 in transcripts generated from the minigene (Figure 7E, lanes 1–3). A G-to-C substitution at the last position of *MBNL1* exon 5 resulted in complete skipping of this exon and fully suppressed the effect of risdiplam (Figure 7E, mutant M7). A recent work has shown a critical role for GA-rich sequences in risdiplam-mediated inclusion of *SMN2* exon 7 (46). To specifically examine the effect of exonic A and G residues on HiR-induced inclusion of *MBNL1* exon 5, we made six additional minigene mutants. Risdiplam retained some ability to stimulate exon 5 inclusion in transcripts generated from all six of these mutants, but to a varying degree. Mutants M1 and M2 incorporated four G-to-A and A-to-G mutations, respectively, in the 5′ portion of exon 5, and still retained a strong ability to respond to risdiplam treatment (Figure 7E). Risdiplam affected exon 5 splicing the least in mutants M4 and M5. M4 contained three G-to-A substitutions from position 24 to 29, while M5 contained four A-to-G substitutions in the region from position 38 to 43 (Figure 7E). Our results indicate that both A and G residue(s) within the middle and 3′ portion of *MBNL1* exon 5 might be required for risdiplam-induced inclusion of this exon. However, mutant M6 that carried four G-to-A substitutions in this exon still retained a significant ability to respond to risdiplam (Figure 7E). These substitutions led to complete elimination of all G residues in the region between position 30 and position 53 of exon 5.

### Context-specific role of *cis-*elements in preferential splice site selection by small molecules

We chose *FOXM1* exon 9 as a model to evaluate the role of *cis-*elements away from the 5′ss in splicing modulation by small molecules. *FOXM1* exon 9 contains two competing 5′ss separated from each other by 80 nt. Usage of the upstream and downstream 5′ss results in inclusion of the short (9S) and long (9L) isoforms of exon 9, respectively. Both risdiplam and branaplam substantially reduced *FOXM1* exon 9 skipping by promoting the usage of both 5′ss, with risdiplam preferentially promoting usage of the downstream 5′ss and branaplam that of the upstream 5′ss (Figure 3C).

**Figure 8.**
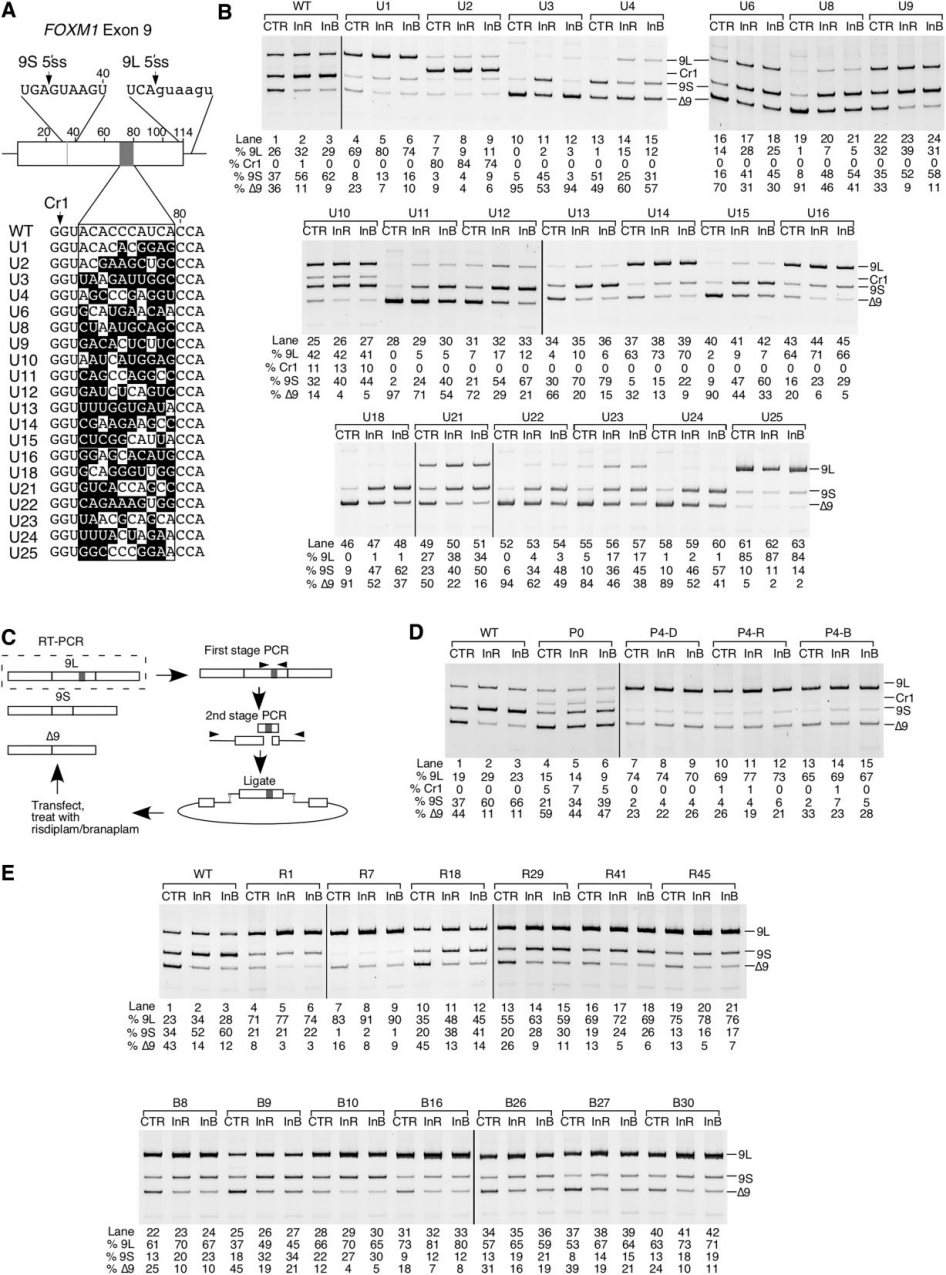
Context-specific role of *cis-*elements in preferential splice site selection by small molecules. (**A**) Location and sequence of a randomized region of the *FOXM1* exon 9 hybrid minigene. The exon is shown as a box, and the flanking intron as lines. The sequences surrounding the 9S and 9L 5′ss are shown above the exon. The randomized region is shown as a gray box, and the sequences of 20 random clones are given below. Numbering is relative to the start of the exon. (**B**) Splicing pattern of the wild-type (WT) *FOXM1* exon 9 hybrid minigene and 20 random clones. Treatments and clone names are indicated above each gel. Splice isoform identities are indicated at the right side. Densitometric quantification of each isoform relative to total RNA expression is indicated at the bottom. (**C**) Overview of the *in vivo* selection process. Binding locations for PCR primers are indicated with arrowheads. Other coloring and labeling is the same as in (A). (**D**) Splicing pattern of the WT *FOXM1* hybrid minigene, pooled unselected clones and the final selected pools. Labeling is the same as in (B). (**E**) Splicing pattern of single clones from the final selected pool. Labeling is the same as in (B).

We generated a pool of *FOXM1* minigenes in which a 10 nt long region from positions 70 to 79 of exon 9L was randomized. This exonic region is located roughly halfway between the 5′ss of exons 9S and 9L. Therefore, this region would be positioned within the intronic sequence if the upstream 5′ss were to be used for inclusion of exon 9S. This allows us to simultaneously probe the role of both exonic and intronic *cis-*elements in the effects of the small molecules on the usage of a specific 5′ss. We randomly selected 20 clones from the pool of minigenes. Our selected clones had a minimum and maximum of four and nine substitutions within the 10 nt stretch, respectively (Figure 8A). The wild-type sequence of the 10 nt region we randomized contains five cytosine, four adenosine and one uridine residue. Hence, our approach also evaluated the effect of the loss of the potential small molecule-responsive AC-rich motif(s) in the 5′ss selection. In order to capture subtle differences between the mutant minigenes, we utilized intermediate concentrations of both drugs (InR, 250 nM; InB, 10 nM) and a shorter treatment time (6 h). We examined the effect of risdiplam and branaplam on three splicing events, namely skipping of exon 9 and inclusion of exons 9S and 9L (Figure 8B). Given the large number of substitutions generated by randomization, we expected some mutations to create cryptic splice sites. Hence, our experimental design also offered an opportunity to ask how small molecules might impact the usage of two competing 5′ss in the presence of a cryptic splice site in between.

We first measured the effect of InR and InB on transcripts generated from the wild-type *FOXM1* minigene. Both InR and InB preferentially promoted inclusion of exon 9S, although a small stimulatory effect on inclusion of exon 9L was also captured (Figure 8B). Supporting the compound-specific differences, the stimulatory effect on inclusion of exon 9L was somewhat stronger in the case of InR than InB. These results are consistent with those observed with 24 h treatment with HiB and HiR (Figure 7C). We next analyzed the splicing pattern of 20 randomly selected mutant minigenes. Supporting the critical role of motifs away from the 5′ss, most mutants showed splicing pattern distinct from those observed with the wild-type minigene (Figure 8B). We identified eight mutants (U3, U8, U11, U15, U18, U22, U23 and U24) that displayed predominant (>80%) skipping of exon 9 and barely detectable levels of exon 9L inclusion. Some of these mutants also showed low but detectable levels of inclusion of exon 9S. While most of these mutants had lost CC- and/or AC-rich motifs, the nature of substitutions varied widely (Figure 8A). Three additional mutants (U6, U12 and U13) showed >60% skipping of exon 9 and different degrees of inclusion of exons 9S and 9L. Here again, the substitutions varied significantly. Mutant U4 that harbored six substitutions showed comparable levels of exon 9 skipping and exon 9S inclusion, accompanied by near total loss of the usage of the downstream 5′ss. Mutant U9 carried eight substitutions and showed a splicing pattern similar to that of the wild-type minigene. Interestingly, exon 9 substitutions in U9 created an 8 nt long pyrimidine-rich motif that incorporated two wild-type residues immediately downstream of the randomized region. Mutant U21 had six substitutions and showed ∼50% exon 9 skipping and ∼27% and ∼23% inclusion of exons 9L and 9S, respectively. This mutant retained the CCA motif from the wild-type sequence as well as acquiring a CCCC motif. In four minigene mutants (U1, U14, U16 and U25), exon 9L was preferentially included, accompanied by decreased skipping of exon 9 and usage of the upstream 5′ss. Of note, these *FOXM1* minigene mutants had all acquired one or more GA-rich motifs that were absent in the wild-type sequence (Figure 8A). We also identified two mutants (U2 and U10) in which a cryptic 5′ss (Cr1) immediately upstream of the area of randomization was activated. In both instances, the usage of Cr1 substantially reduced skipping of exon 9. In the case of mutant U2, activation of Cr1 also decreased the usage of both the upstream and downstream 5′ss. In the case of the U10 mutant, activation of Cr1 enhanced usage of the downstream 5′ss, while usage of the upstream 5′ss was slightly reduced. We did not observe a single minigene mutant in which inclusion of exon 9S was the major splicing event.

We compared the effect of InR and InB on splicing of the above mutants. With the exception of U3 and U4, mutants with enhanced skipping of exon 9 showed increased inclusion of exon 9S upon treatment with InR and InB (Figure 8B). These results suggested that our chosen region of investigation was devoid of *cis-*elements associated with the small molecule-induced inclusion of exon 9S. In the case of the U3 mutant minigene, only risdiplam but not branaplam promoted inclusion of exon 9S, underscoring the difference in composition and/or positioning of motifs that respond differently to risdiplam and branaplam (Figure 8B). Such motifs may fall outside of the 10 nt long sequence that we randomized but may still be responsive to *trans*-factors that are recruited by *cis-*elements or modified by structures generated as a result of randomization. The results of U4 minigene splicing were rather surprising as both InR and InB reduced the usage of the upstream 5′ss but promoted the usage of the downstream 5′ss and enhanced the skipping of exon 9 (Figure 8B). Once again, these results underscored the subtle differences in the mechanism of action of branaplam and risdiplam in selection of a specific 5′ss. The presence of a strong cryptic 5′ss (Cr1) in mutants U2 and U10 completely suppressed the ability of risdiplam and branaplam to promote usage of the downstream 5′ss, but not the upstream one. This is probably due to sequestration of small molecule-responsive *cis-*elements by U1 snRNP recruited at the cryptic 5′ss. It is also possible that the small molecule-responsive *cis-*elements recruit low affinity binding factors that are easily displaceable by U1 snRNP recruited at Cr1.

One of the major findings of our *FOXM1* mutant minigene screening was the disproportionate usage of the two 5′ss in the presence of small molecules. For example, a small molecule-dependent increase in the usage of the downstream 5′ss was always accompanied by an increase in the usage of the upstream 5′ss. However, an increase in the usage of the upstream 5′ss did not always result in an increase in the usage of the downstream 5′ss. This could be due to the loss of one of the small molecule-responsive motifs that favor the usage of the downstream 5′ss. It is also possible that factors recruited by *cis-*elements created by substitutions directly or indirectly inhibit the usage of the downstream 5′ss. To identify small molecule-responsive motifs that exclusively favor the usage of the downstream 5′ss, we performed an *in vivo* selection experiment. This experiment also allowed us to test whether small molecule-responsive intronic motifs that favor usage of the upstream 5′ss could be fully eliminated. We transfected HeLa cells with the pool of *FOXM1* exon 9 minigenes containing the 10 randomized bases (see above). We then treated cells with InR or InB for 6 h and collected total RNA. Following RT–PCR of minigene-derived transcripts, we isolated the PCR fragment corresponding to the exon 9L-included product. We then used two-stage PCR to regenerate the full-length *FOXM1* minigene pool, which was used for the next round of selection (Figure 8C). We performed four rounds of selection for exon 9L inclusion in total (Figure 8D; Supplementary Figure S13). Over the first three rounds, we observed a steady increase in the proportion of the exon 9L band in all three treatments (CTR, InR and InB) (Supplementary Figure S13). By round 4, exon 9L inclusion had stopped increasing, indicating that we had reached the limits of selection. We then analyzed 48 randomly chosen *FOXM1* minigenes from pool 4 selected in the presence of InR and InB by sequencing and determining their splicing pattern (Supplementary Tables S2 and S3). Confirming that the selection was nearly complete, we observed several duplicate sequences in the selected minigene clones. The majority of clones had extremely high inclusion of exon 9L even without the treatment with either small molecule. This suggested that the selection primarily favored small molecule-independent motifs that strengthened the downstream 5′ss. Consistently, motifs selected in the presence of risdiplam and branaplam were similar in compositions, mostly centered around [CU]GGACC, with some slight differences depending on the parameters chosen for motif identification (Supplementary Table S4). Yet we were able to isolate many minigenes that retained significant skipping of exon 9. We used these minigenes to evaluate the effect of the small molecules on the usage of both the downstream and upstream 5′ss. Interestingly, most of these minigenes showed an increase in the usage of the upstream 5′ss in the presence of InR and InB (Figure 8D). These results once again supported that small molecule-responsive motif(s) involved in the usage of the upstream 5′ss fall outside of the randomized region. We had hoped to select at least one clone with the capability to suppress skipping of exon 9 in addition to specifically promoting usage of the downstream 5′ss. However, our selection process appeared to be primarily driven by sequence motifs that promoted inclusion of exon 9L even in the absence of risdiplam and branaplam. Our results suggest that the selection for a particular FOXM1 exon 9 splicing event was driven by *cis-*elements in the small molecule-independent mode. Future experiments will determine if small molecules are capable of suppressing the usage of a competing 5′ss while also preventing the skipping of an exon.

### Exonic motifs associated with the *SMN2* exon 7 splicing modulation by risdiplam and branaplam

**Figure 9.**
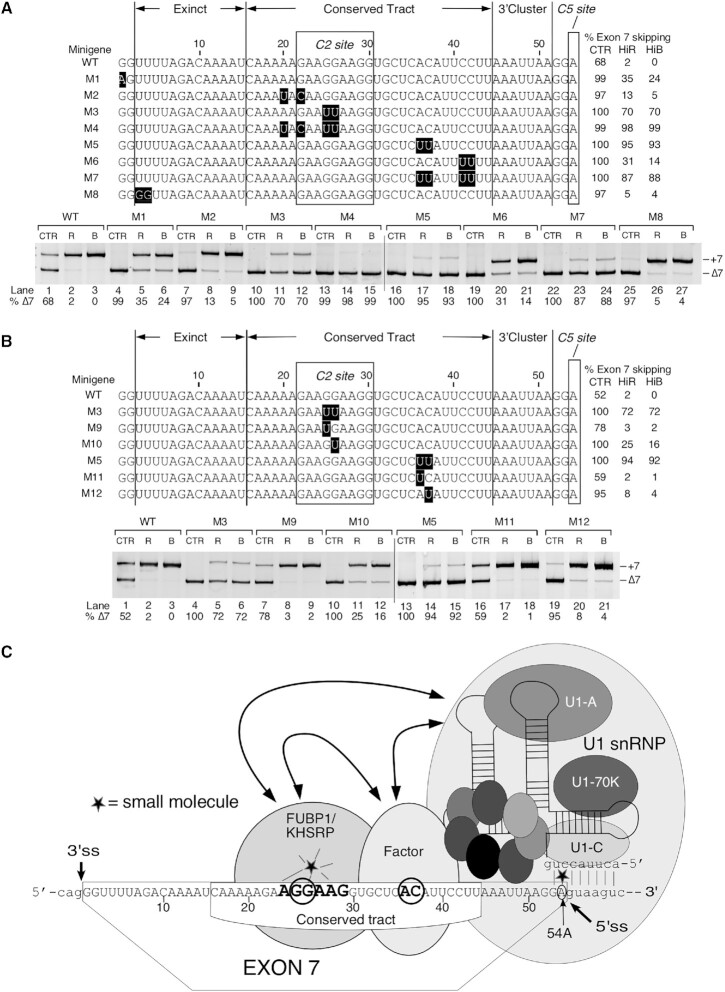
Exonic mutations affect the ability of risdiplam and branaplam to promote exon 7 inclusion in transcripts generated from *SMN2* minigenes. (**A**) Splicing of transcripts produced from *SMN2ΔI6* minigenes carrying exonic mutations in HeLa cells. The sequence of the entire exon 7 for each minigene construct is shown in the upper panel. Minigene names are given on the left. Type of treatment and percentage of minigene exon 7 skipping are indicated on the right. Mutated nucleotides are highlighted in white on a black background. Three regions, Exinct, the Conserved tract and 3′ Cluster, shown to affect splicing of *SMN* exon 7 are indicated (13). C2 and C5 sites show where risdiplam C2 and C5 analogs interact with *SMN* exon 7 (45,46). A representative gel image showing the splicing pattern of *SMN2* minigenes is given in the lower panel. Treatments and minigene names are indicated at the top of the gel image. Labeling is the same as in Figure 1B. (**B**) Splicing of *SMN2* minigenes carrying single versus double mutations in HeLa cells. Labeling is the same as in (A). (**C**) Diagrammatic representation of the mechanism of small molecule-induced recruitment of U1 snRNP through GG and AC motifs. The sequence of *SMN2* exon 7 and surrounding intronic sequences are given. Numbering is relative to the beginning of exon 7. Splicing factors are shown as shaded circles. Critical nucleotides for activity of risdiplam and branaplam are circled. FUBP1/KHSRP interacts with both the GG-rich motif and the small molecule. FUBP1/KHSRP may directly contact the downstream AC residues or help to recruit another factor that interacts with the downstream AC residues. Upstream RNA–protein and protein–protein interactions induced by the small molecule facilitate recruitment of U1 snRNP at the 5′ss that independently interacts with the small molecule and U1 snRNA.

The reported mechanisms of risdiplam stimulation of *SMN2* exon 7 inclusion are based on studies with risdiplam analogs C5 and C2. While C5 contacts 54A, an A residue at the last exonic position (47), C2 interacts with an GA-rich motif (GAAGGAAGG) located in the middle of the exon (45,46) (Figure 9). It has been proposed that 54A and the GA-rich motif serve as the primary and secondary binding sites of risdiplam (46). Currently, it is not known if additional exonic motifs may contribute to risdiplam-induced inclusion of *SMN2* exon 7. The proposed mechanism of branaplam action on *SMN2* exon 7 inclusion is based on studies with one of the branaplam analogs, NVS-SM2, which is shown to stabilize the interaction of U1 snRNP with the 5′ss of *SMN2* exon 7 (48). Currently, it is not known whether the above-mentioned GA-rich motif in the middle of *SMN2* exon 7 may also be involved in the branaplam-linked increase in exon 7 inclusion. We hypothesized that additional exonic nucleotides/motifs may play a role in the risdiplam- and branaplam-elicited effect on exon 7 splicing. In search of such motifs, we utilized a library of *SMN* minigene mutants with partially randomized exon 7 sequences. We used only those mutants that showed predominant skipping of exon 7 (Supplementary Figure S14). In many cases, both compounds failed to stimulate exon 7 inclusion despite the presence of 54A, as well as absence of the inhibitory C6U mutation associated with skipping of exon 7 (Supplementary Figure S14). For example, neither risdiplam nor branaplam was able to promote exon 7 inclusion in the 00–49NS minigene, in which exon 7 contained 54A and the intact GA-rich motif, and did not carry the inhibitory C6U mutation (Supplementary Figure S14). These results implicate the potential involvement of additional exonic sequences and/or factors associated with them in stimulation of *SMN* exon 7 inclusion by the compounds.

Using *in vivo* selection of the entire exon, we previously reported three regions that contribute to *SMN* exon 7 splicing regulation. These are extended inhibitory tract (Exinct), the conserved tract and 3′-cluster (13). Exinct and 3′-cluster are negative regulators, while the conserved tract is a positive regulator of exon 7 splicing (13). The above-mentioned GA-rich motif falls within the conserved tract (Figure 9). We tested the effect of HiR and HiB on splicing of *SMN2* exon 7 that carried single, double and quadruple substitutions known to promote exon 7 skipping. As previously reported, a single G-to-A substitution at the first exonic position (mutant M1) caused nearly total skipping of *SMN2* exon 7 (Figure 8A, lane 4) (13). Yet, both compounds were able to rescue inclusion of the exon with this mutation (Figure 8A, lanes 5–6). Similarly, the inhibitory effect of U-to-G substitutions at the third and fourth positions of exon 7 on its splicing was overcome by HiR and HiB treatment (Figure 9). As expected, substitutions within the conserved tract increased exon 7 skipping. Most of these mutations also negatively impacted the ability of the compounds to promote exon 7 inclusion (Figure 9). Mutant M5 is of particular interest, since it carried a double nucleotide substitution located outside of two proposed sites of risdiplam/branaplam action (AC-to-UU substitutions at positions 36 and 37), and yet these substitutions severely impacted the ability of both compounds to promote exon 7 inclusion (Figure 9A, B). Based on these results and a previous report, we propose that GG residues at the 25th and 26th positions and AC residues at the 36th and 37th positions are critical for small molecule-induced inclusion of *SMN* exon 7. Previous reports support the role of risdiplam-induced recruitment of FUBP1 and/or KHSRP at the GA-rich motif within exon 7 (45). It is likely that FUBP1 and/or KHSRP also contact AC residues at the 36th and 37th positions of exon 7. Alternatively, it is possible that an additional factor that contacts the AC motif is recruited by FUBP1 and/or KHSRP. Such a mechanism may be mutually inclusive to the small molecule-mediated recruitment of U1 snRNP at the 5′ss of exon 7 (Figure 9C).

### Effect of combined treatment of risdiplam and branaplam

**Figure 10.**
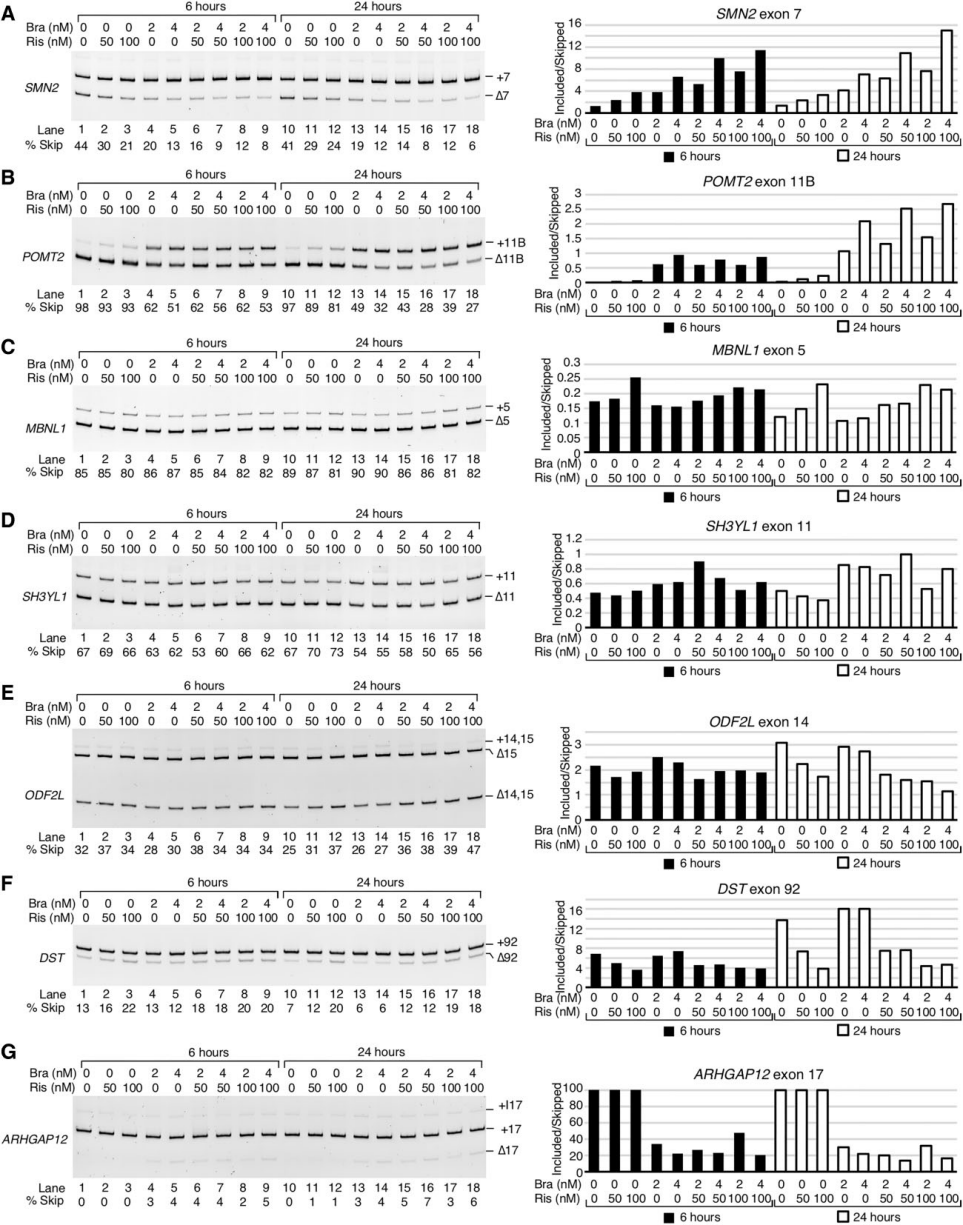
Combined treatment of risdiplam and branaplam. (**A**) Splicing of *SMN2* exon 7 in the presence of low concentrations of risdiplam and/or branaplam. Left panel: representative gel image of RT–PCR after 6 or 24 h of treatment of GM03813 fibroblasts is shown. Treatment times and concentrations are indicated at the top of the gel. Other labeling is the same as Figure 1B. Right panel: bar diagram depicting the inclusion:skipping ratio as determined by densitometric quantitation of the gel in the left panel. Black bars represent 6 h treatment and white bars represent 24 h treatment. (**B**–**G**) Splicing of *POMT2* exon 11B (**B**), *MBNL1* exon 5 (**C**), *SH3YL1* exon 11 (**D**), *ODF2L* exon 14 (**E**), *DST* exon 92 (**F**) and *ARHGAP12* exon 17 (**G**) in the presence of low concentrations of risdiplam and branaplam. Labeling is the same as in (A).

We examined the effect of the combined treatment of risdiplam and branaplam at two concentrations and at two time points using GM03813 fibroblasts. At 50 nM risdiplam and 2 nM branaplam, we observed a reduction in *SMN2* exon 7 skipping from 44% to 30% and 20% at 6 h of treatment, respectively (Figure 10A). There was an ∼1.6-fold increase in *SMN2* exon 7 inclusion when concentrations of compounds were doubled. Similar results were obtained when treatment times were increased from 6 h to 24 h. When 50 or 100 nM risdiplam was combined with 2 or 4 nM branaplam, we observed a dose-dependent increase in the stimulatory effect on inclusion of *SMN2* exon 7 at both 6 and 24 h of treatment. However, unlike the outcome of the individual treatments, some of the combined treatments elicited a noticeably stronger response at 24 h in comparison with 6 h (Figure 10A, right panel). Based on the calculations of the combined effects of the treatments (121), all 24 h treatments showed additive and/or synergistic effects. The strongest synergistic effect was observed at 24 h with 100 nM risdiplam and 4 nM branaplam. Interestingly, for a given concentration of risdiplam, increasing the concentration of branaplam was more beneficial, showing a greater effect on *SMN2* exon 7 inclusion than vice versa. For example, at 50 nM risdiplam, we observed a remarkable ∼1.90-fold increase in exon 7 inclusion at 6 and 24 h when the branaplam concentration was doubled. In contrast, at 2 nM branaplam, we observed only a 1.20- and 1.37-fold increase in exon 7 inclusion at 6 and 24 h, respectively, when the risdiplam concentration was doubled. We saw a similar trend when the effect of doubling branaplam concentrations in the presence of 100 nM risdiplam was compared with that of doubling risdiplam concentration in the presence of 4 nM branaplam. A better outcome of increasing concentrations of branaplam rather than risdiplam in combined treatments could be due to multiple factors including diminishing potential negative off-target effects of risdiplam by keeping it low combined with indirect positive effects of branaplam on *SMN2* exon 7 splicing.

To gain insights into the effects of combined treatments on off-target splicing events, we analyzed the splicing pattern of representative exons shown to be impacted by risdiplam and/or branaplam, including *POMT2* exon 11, *MBNL1* exon 5, *SH3YL1* exon 11, *ODF2L* exons 14 and 15, *DST* exon 92 and *ARHGAP12* exon 17 (Figure 10B–G). Our PCR results confirmed the stimulatory effect of all concentrations of the compounds used except for LoR in the case of *POMT2* exon 11B (Figure 3C). Consistently, at 6 h of treatment, 50 and 100 nM risdiplam showed a negligible effect on splicing of *POMT2* exon 11, whereas 2 and 4 nM branaplam noticeably stimulated inclusion of this exon (Figure 10B). At 6 h, we did not observe any additive effect of the combined treatment of compounds. However, effects became additive or slightly synergistic when the treatment time was increased from 6 h to 24 h (Figure 10B). Inclusion of *MBNL1* exon 5 and SH3YL1 exon 11 was stimulated by risdiplam alone and branaplam alone, respectively (Figure 3). Splicing of neither exon was strongly affected at low concentrations of the compounds. Consistently, we observed only mild effects of the combined treatments on splicing of *MBNL1* exon 5 and *SH3YL1* exon 11 (Figure 10C, D). Co-skipping of *ODF2L* exons 14 and 15 was promoted by both compounds, although the effect was less significant at LoR and LoB (Figure 4C). Hence, only the combined treatment using high concentrations of the compounds produced an appreciable effect on co-skipping of *ODF2L* exons 14 and 15 at 24 h (Figure 10E). Considering that skipping of *DST* exon 92 is induced by risdiplam but not by branaplam (Figure 4B), we noted a small effect of risdiplam but no effect of branaplam or an added effect of both compounds on *DST* exon 92 at 6 or 24 h (Figure 10F). Skipping of *ARHGAP12* exon 17 is promoted by higher concentrations of branaplam (Figure 4D). However, at the concentrations of branaplam and risdiplam used for the combined treatments, we did not observe any significant changes in splicing of this exon (Figure 10G). We also examined the effect of the combined treatments on transcription of five genes impacted by risdiplam and/or branaplam, namely *YTHDF2*, *ZFP82*, *LIG1*, *EGR1* and *DOCK11* (Supplementary Figure S15). Except for *YTHDF2* that showed an ∼1.8-fold increase in expression levels in response to 100 nM risdiplam, we did not observe any changes at 6 h treatments with individual compounds. We did not capture any noticeable change in expression levels of these genes using the combined treatments at the 6 h time point. At the 24 h time point, treatments with 100 nM risdiplam caused ∼1.4- and ∼1.5-fold increases in expression of *YTHDF2* and *DOCK11*, respectively. At the same time, expression of *DOCK11* was significantly downregulated by both concentrations of branaplam. In addition, we captured a very small but significant decrease in expression of LIG1 at 24 h of treatment with 50 nM risdiplam. However, this decrease was not observed in the case of the combination treatments. Overall, our results supported the benefits of the combined treatments using lower concentrations of the compounds on *SMN2* exon 7 splicing with seemingly minimized off-target effects.

## DISCUSSION

Here we examine the transcriptome-wide off-target effects of risdiplam and branaplam, two splicing-modulating small molecules designed for the treatment of SMA. We analyzed the transcriptome of SMA patient fibroblasts treated with 50 nM (LoR) and 1000 nM (HiR) risdiplam. For branaplam, we used concentrations of 2 nM (LoB) and 40 nM (HiB). The highest concentrations employed in this study fall within the concentration ranges of those reported in *in vitro* and *in vivo* studies (37–40,44–48). Expression levels of 10921 and 2187 genes were significantly altered by HiR and HiB, respectively. LoR and LoB affected expression of 566 and 15 genes, respectively. Both compounds impacted genes located on all chromosomes, while genes producing between 2 and 15 alternatively spliced transcripts were affected the most. The unexpected high perturbations of the transcriptome caused by HiR could be attributed to its adverse effects on processes upstream of protein synthesis such as DNA replication, DNA repair, spliceosome function, mRNA transport, mRNA surveillance, ribosome biogenesis and ribosome function. In contrast, top pathways impacted by HiB were primarily signaling cascades. The highly expressed genes were more upregulated than downregulated by HiR, whereas a nearly equal number of the highly expressed genes were upregulated and downregulated by HiB. A recent report showed a tight interaction of a risdiplam analog with GA-rich motifs in single-stranded regions of DNA (46). It is possible that risdiplam interacts with GA-rich motifs in promoter regions in a similar fashion, increasing DNA accessibility for positive or negative transcription factors recruited in a context-specific manner. Incidentally, the promoter region of risdiplam-upregulated genes harbors GA-rich motifs that bear a resemblance to that of the target genes of several zinc finger proteins (Supplementary Figure S3). In particular, analysis of RNA-Seq data supported the association of ZNF148 in regulation of genes impacted by risdiplam (Supplementary Figure S2C). Both compounds caused abnormal expression of lncRNAs. Given potential interactions of small molecules with unique structures formed by lncRNAs and the role of lncRNAs in transcription (122), it is possible that some of the transcriptional changes are brought about by the perturbed status of lncRNAs. rRNAs are known targets of small molecules due to their complex secondary and high-order structures (123). A potential rRNA structure stabilized by interactions with a small molecule may lead to the deregulation of ribosome assembly. Hence, it is conceivable that risdiplam-induced up-regulation of genes associated with ribosome biogenesis is triggered by a feedback mechanism due to improper ribosomal assembly.

For validation purposes, we used intermediate concentrations in addition to the lowest and the highest concentrations of the compounds. Our validations confirmed the aberrant expression of 14 genes randomly selected from RNA-Seq data. In most instances, the intermediate concentrations of the compounds also produced the expected changes in gene expression that were captured at the highest concentrations of the compounds. Each affected gene we validated is associated with important function(s) and/or linked to pathological condition(s). Our time course experiment captured noticeable changes in the expression of many genes at or before 6 h of the treatment with HiR and HiB. These early affected genes, including *CACNB1*, *ZFP82*, *PDXDC1*, *EGR1*, *YTHDF2*, *GGNBP2*, *DOCK11* and *PDS5B*, could be considered as the primary targets of HiR and/HiB (Figure 2I–N). Aberrantly expressed primary targets at the early time points may unleash cascading secondary effects resulting in the perturbed levels of transcripts we captured at 24 h of treatments. Given the role of YTHDF2 in RNA decay (61), it is likely that many of the downregulated transcripts observed at 24 h could be due to their degradation by YTHDF2 in the case of the treatment with HiR. Transcripts downregulated by YTHDF2 may include transcription and/or splicing factors with potential to produce even larger downstream effects. Although not examined in this study, important protein factors could also be the primary targets of the drugs. Perturbed protein structures, aberrant post-translational modifications and mislocalizations of proteins have potential to alter the relative abundance of transcripts that we analyzed at 24 h treatment with HiR and HiB.

Both compounds impacted seven types of mechanistically distinct splicing events: EIN, ESK, IRT, IRM, A5S, A3S and MXE. We captured a total of 2385 and 835 aberrant splicing events caused by HiR and HiB, respectively. However, these numbers should be interpreted with caution as some of the alternatively spliced transcripts harboring premature stop codons could be degraded by nonsense-mediated decay and/or other surveillance mechanisms (124). ESK and EIN were the most frequent events triggered by HiR and HiB, respectively. There were only 54 EIN and 44 ESK events shared between HiR and HiB, underscoring the differences in their mechanism of action on splicing regulation. LoR and LoB triggered aberrant splicing affecting 58 and 76 events, respectively. We observed limited overlaps between splicing events affected by HiR and LoR, suggesting that additional targets were engaged at high risdiplam concentrations. In contrast, a larger overlap in splicing events affected by HiB and LoB suggested that the HiB concentration was not high enough to engage widespread targets and cause massive perturbations in splicing. Both transcription and splicing affect each other through complex and poorly understood mechanisms in which the structure of chromatin, promoter elements and *cis-*elements within nascent RNA attached to RNA polymerase II determine the rate of transcription and the outcome of splicing (125). Our analysis revealed a potential connectivity between transcript levels and the compound-specific splicing events, including EIN, ESK, IRM and IRT. The impacted splicing events were observed in both up- and downregulated genes, suggesting diverse mechanisms were at play in transcription-coupled splicing regulation. Notable EIN events linked to increased gene expression were the risdiplam-specific inclusion of *MBNL1* exon 5, branaplam-specific inclusion of *KDM6A* exon 28 and *POMT2* exon 11B triggered by both compounds (Figure 3C; Supplementary Tables S5 and S6). Of note, although HiB promoted inclusion of *POMT2* exon 11B more efficiently than HiR (Figure 3C), the greater change in expression of this gene was observed at HiR, suggesting that the correlation between splicing and transcription may not be entirely straightforward (Supplementary Tables S5 and S6). *ANXA11* exon 16B inclusion promoted by HiB only was accompanied by decreased gene expression (Figure 3D; Supplementary Table S6). Enhanced skipping of *TEAD1* exon 5B and *THOC5* exon 10 observed under HiR treatment was associated with increased gene expression (Figure 4B; Supplementary Table S7). Both of the HiR-triggered IRM events that involved *RBM5* intron 6 and *SPOCD1* intron 12 coincided with an increase in total transcript levels (Figure 6B; Supplementary Figure S7D; Supplementary Table S8). Although IRT was generally associated with lower gene expression in HiB treatment, *SMTN* intron 17 retention was associated with an increase in transcript levels (Supplementary Figure S7H; Supplementary Table S9).

We validated the aberrant splicing events caused by risdiplam and branaplam using gel-based assays that accurately captured the exon-skipped and exon-included transcripts in the same lane. For quantification of the intron-retained transcripts, we used a qPCR-based assay. We performed our validations at three concentrations. In most instances, we were able to capture effects at the intermediate concentrations of the compounds. These validations confirmed the effect of risdiplam and/or branaplam on six categories of splicing events, namely EIN, ESK, A5S, A3S, IRM and IRT. For EIN events, we evaluated the conservation of the 8 nt long motif encompassing the last two nucleotides of the exon and the first six nucleotides of the downstream intron. We did not find a major deviation from the consensus 5′ss (GUAAGU), except for the sixth intronic position where the U residue was the least conserved. In the case of HiR-induced EIN, we observed the usual representation of >55% A and >75% G residues at the –2 and –1 exonic positions, respectively. These results support that an A residue at the last exonic position is not a general determinant for an EIN event affected by risdiplam (44). Consistent with previous studies, G and A residues at positions –2 and –1, respectively, were preferred in the case of EIN induced by HiB (48). Unlike EIN events that affected exons of all sizes, ESK events triggered by HiR and/or HiB preferentially influenced small exons. According to our analysis, ESK events generally occurred in exons with a weak 3′ss, although a weak 5′ss was also a factor in cases where ESK was triggered by both compounds. There were no preferred consensus motifs associated with A5S, A3S, IRM and IRT events caused by the two compounds.

The impact of risdiplam and branaplam on splicing could be due to direct engagement with the target and/or through indirect effects via alteration of concentrations/localizations of splicing factors. An effect of a compound on splicing at the early time points of treatment would support a direct engagement with the target. Indeed, we captured several splicing events as early as at 3 h of treatment with HiR and HiB. These are inclusion of *SMN2* exon 7, *POMT2* exon 11 and *SH3YL1* exon 11 as well as skipping of *ODF2L* exon 14, *DST* exon 92 and *ARHGAP12* exon 17 (Supplementary Figure S16). To further examine a direct interaction of the compounds with their targets, we employed minigenes that largely recapitulated the effects of the compounds on splicing of their corresponding endogenous genes (Figure 7; Supplementary Figure S8). Employing minigenes, we confirmed splicing of several exons, including *TTC28* exon 20, *TMEM50B* exon 5B, *SH3YL1* exon 11, *ARHGAP12* exon 17, *ATG5* exon 3 and *MBNL1* exon 5, that did not encompass an A residue at the last exonic position, yet responded to the treatment with risdiplam/branaplam. In the *TMEM50B* exon 5B mutant that carried a U-to-C substitution at the last exonic position, the ability of branaplam to promote inclusion of this exon was preserved (Figure 7B). An A-to-C mutation at the last exonic position of *FOXM1* exon 9L did not abrogate the stimulatory effect of risdiplam on exon 9L inclusion (Figure 7C). However, abrogation of exonic AC-rich motifs inhibited the ability of the small molecules to stimulate *FOXM1* exon 9L inclusion (Figure 8B). These results supported the role of novel small molecule-responsive *cis-*elements upstream of the 5′ss in alternative 5′ss selection. In the case of *MBNL1* exon 5, a G-to-C mutation at the last exonic position abolished the ability of risdiplam to stimulate exon 5 inclusion (Figure 7E). On the other hand, a G-to-C mutation at the last exonic position of *ATG5* exon 3 enhanced the ability of branaplam to stimulate exon 3 skipping (Figure 7D). These results support that both compounds modulate splicing in a context-specific manner where the last exonic position together with other *cis-*elements may play an important role. We should also keep in mind that RNA structure plays a pivotal role in splicing regulation (126,127). Secondary and high order RNA structures are proposed to serve as potential targets of small molecule therapeutics (122).

High concentrations of risdiplam and branaplam have been recently shown to interact with small motifs in the bulged region of the secondary structures of RNAs (128). The predicted secondary structures of exons/introns affected by the compounds show similar motifs within the single-stranded regions (Supplementary Figures S9–S12). Hence, it is likely that these motifs provide interaction pockets for risdiplam and branaplam. Splicing is regulated by a combinatorial control of *cis-*elements and*trans*-acting factors that define the context of an exon (129–131). Our screening of randomized sequences of *SMN* exon 7 showed the inability of the compounds to promote exon inclusion when it is severely inhibited by mutations within the exons (Supplementary Figure S14). However, not all inhibitory mutations suppressed the stimulatory effects of the compounds. In particular, mutations that weakened the 3′ss of *SMN* exon 7 had the least effect on the ability of the compounds to stimulate exon 7 inclusion. We hypothesize that exonic mutations that interfere with recognition of the 5′ss of exon 7 would suppress the ability of the compounds to promote inclusion of this exon. Such exonic mutations may not be restricted to the GA-rich motifs proposed to interact with risdiplam. Supporting this argument, we uncovered the role of a novel AC-containing exonic motif in small molecule-induced promotion of *SMN* exon 7 inclusion (Figure 9). Similarly, we captured the role of AC-rich exonic motif(s) in the small molecule-induced enhanced usage of the downstream 5′ss in the case of *FOXM1* exon 9 (Figure 8). It is likely that the small molecules directly or indirectly help recruit factors that recognize AC-containing exonic motifs. Such recruitment may be enabled by a small molecule-assisted interaction of FUBP1 and/or KHSRP with the upstream GA-rich motif as proposed in the case of risdiplam (45). Interestingly, a GA-rich motif is also present upstream of the AC-rich motif within *FOXM1* exon 9 (Supplementary Figure S11). It is possible that the small molecule-mediated network of RNA–protein and protein–protein interactions on the exonic sequences may facilitate recruitment of U1 snRNP to the 5′ss, which itself is shown to independently interact with the small molecule and U1 snRNA (Figure 9C). Based on the observation that the same mutation(s) influenced the effects of risdiplam and branaplam on splicing of *SMN* exon 7 and *FOXM1* exon 9 (Figures 8 and 9), it is likely that these compounds share common mechanisms of splicing regulation in these instances.

SMA is a model disease in which multiple approaches could be employed to modulate *SMN2* exon 7 splicing as a potential therapy (8). However, therapeutic benefits of a splicing-modulating compound are not guaranteed without proper evaluation of its off-target effects. The long-term consequence of off-target effects could be detrimental if repeated high doses are administered during the course of the protracted treatment. A recent study in a mouse model of severe SMA showed better efficacy of risdiplam when combined with an ISS-N1-targeting ASO (132). However, high efficacy of splicing-modulating drugs observed in *in vivo* studies in mouse models cannot be extrapolated to humans given the divergences of intronic sequences that harbor the vast repertoire of human-specific regulatory elements. With the universal availability of high-throughput sequencing at an affordable cost, it is now possible to examine the genome-wide off-target effects at the very outset of a drug development process. A systematic evaluation of off-target effects is critically important not only for understanding the mechanism of drug action but also for developing the next generation of highly specific drugs. Additionally, a thorough evaluation of the concentration-dependent effects of a drug in question on the transcriptome would help determine the dosing and frequency of drug administration. While the recent approval of the splicing modulator risdiplam for the treatment of SMA is welcome news, many concerns remain due to uncomfortable side effects including rash, fever, diarrhea, joint pain (arthralgia), ulcers of the mouth area and urinary tract infections (39). It is likely that some of these side effects could be attributed at least in part to the off-target effects we report here. The relatively fewer off-target effects associated with branaplam could be due to the high efficacy of this compound at low nanomolar concentrations. Yet, we captured considerable perturbations in gene expression and pre-mRNA splicing, especially aberrant exon inclusion, even at 40 nM branaplam. Interestingly, branaplam is also being considered for the treatment of Huntington's disease (133). Hence, findings reported here have relevance for diseases other than SMA. The contrasting account of the off-target effects of two splicing-modulating small molecules provides a general insight into how critically important the concentration of a compound used for therapeutic intervention could be. On an optimistic note, both compounds showed limited off-target effects at low concentrations. Branaplam in particular remained highly efficacious at a concentration as low as 2 nM while changing the expression of only 15 genes. The results of our combination treatments showed strong stimulatory effect on *SMN2* exon 7 splicing at low doses of the compounds, with potentially minimized off-target disturbances. These findings augur well for developing the next generation of small molecule therapeutics with even better efficacies and fewer off-target effects.

## DATA AVAILABILITY

All RNA-Seq data are available publicly at the NCBI Sequence Read Archive, accession number SRP334251.

## Supplementary Material

gkad259_Supplemental_Files
