# Supplementary material for: Diverse targets of SMN2-directed splicing-modulating small molecule therapeutics for spinal muscular atrophy
Source: Nucleic Acids Res. 2023 Apr 7;51(12):5948–80. doi: 10.1093/nar/gkad259 (PMC10325915; doi:10.1093/nar/gkad259)

SUPPLEMENTARY DATA

Supplementary Figure legends

Supplementary Figure S1. (A) Optimization of risdiplam and branaplam dosage for RNA-Seq study. Representative gel image showing splicing pattern of *SMN2* exon 7 in GM03813 fibroblasts treated with the indicated concentrations of risdiplam and branaplam. Splice isoform identity is marked at the right side of the gel. Quantified relative skipping of *SMN2* exon 7 is indicated below the gel. CTR: 0.1% DMSO control treatment. (B) Genomic views of RNA-Seq reads examining effect of risdiplam and branaplam on splicing of *SMN2* exons in GM03813 fibroblasts. (C) MESDA showing the relative abundance of *SMN2* splice variants produced in GM03813 fibroblasts treated with risdiplam and branaplam.

Supplementary Figure S2. (A) Chromosomal distribution of genes with decreased (blue) or increased (orange) expression in the presence of HiR, compared to all genes expressed in GM03813 fibroblasts (gray). Y axis represents the relative proportion of genes located on each chromosome relative to the total genes in each category, X axis represents chromosomal position of genes. (B) Number of unique transcripts of genes with decreased (blue) or increased (orange) expression in the presence of HiR, compared to all genes expressed in GM03813 fibroblasts (gray). Y axis represents proportion of genes, X axis represents number of annotated transcripts belonging to each gene in question. (C) The 10 most significant transcription factors whose targets are enriched in genes downregulated (left panel) and upregulated (right panel) by HiR. Each transcription factor identity indicated at the left side, number of significantly affected target genes is indicated at the right side. X axis indicates statistical significance. (D) Chromosomal distribution of genes affected by HiB treatment. Coloring and labeling are the same as in (A). (E) Number of unique transcripts of genes affected by HiB. Coloring and labeling are the same as in (B). (F) The 10 most significant transcription factors whose targets are enriched in genes downregulated (left panel) and upregulated (right panel) by HiB. Labeling is the same as in (C).

Supplementary Figure S3. Motifs enriched in promoters of genes whose expression is affected by risdiplam and branaplam treatment. (A-D) The most highly enriched motifs (upper panel) and their reverse-complement (lower panel) in the promoters of genes that were upregulated in HiR (A), upregulated by HiB (B), downregulated by HiR (C), or downregulated by HiB (D). 200 nt-long sequences located immediately upstream to the start of the 200 most highly affected genes were used to calculate motif enrichment. Letter height of sequence logo represents relative enrichment at each position. The number of occurrences of each motif and the *E* value that represents statistical significance are indicated below. (E) The top 4 known motifs that are most similar to the enriched motif shown in (A). (F) Sequences surrounding the transcription start sites (TSS) of upregulated (top panel) and downregulated (bottom panel) genes after treatments with risdiplam and branaplam. Gene names are indicated at the left. Nucleotides are numbered relative to TSS, and numbers are given at the bottom of each panel. TSS is indicated with an arrow and marked in blue. GA-rich motifs are boxed in red. The response of each gene to risdiplam and/or branaplam is indicated to the right of the sequences.

Supplementary Figure S4. Characteristics of exons undergoing EIN caused by risdiplam and/or branaplam treatment. (A) Sequence logos reflecting conservation of nucleotides surrounding the

5′ss of exons whose inclusion was increased by HiR alone (left), HiB alone (middle), or by both treatments (right). Height of each letter indicates relative conservation. (B) Table summarizing splicing-relevant information regarding the top candidate exons with increased inclusion after HiR treatment only, both treatments, and by HiB treatment only. Shown sequences correspond to 12 nucleotides located upstream and downstream of the 3′ and 5′ splice sites (ss). Intronic and exonic sequences are indicated; in addition, exonic sequences are boxed. The 3′ and 5′ ss are shown by arrows. The identity of each exon is given at the beginning of each row. Whether each exon inclusion was affected by HiR or HiB treatment only, or by both is denoted at the right side. ΔSkip indicates the change in the proportion of total transcript that has skipping of the indicated exon. 3′ss and 5′ss scores indicate the predicted strength of the splice sites as compared to the consensus sites. The last three columns indicate the sizes of the upstream intron, alternative exon, and downstream intron.

Supplementary Figure S5. Characteristics of exons undergoing ESK in risdiplam and/or branaplam treatment. Table summarizing splicing-relevant information regarding the top candidate exons with increased skipping after HiR treatment only, both treatments, and after HiB treatment only. Table arrangement is similar to Supplementary Figure S3A.

Supplementary Figure S6. Characteristics of exons undergoing A5S or A3S in risdiplam and/or branaplam treatment. (A) Table summarizing splicing-relevant information regarding the top candidate exons with altered 5′ss usage after HiR treatment only, both treatments, or after HiB treatment only. Sequences represent 12 bases upstream and downstream of the two 5′ss with increased (left) and decreased usage (right) after small compound treatment. Intronic and exonic sequences are indicated, exonic sequences are boxed. The 5′ss score of each splice site is indicated to the right of each sequence. ΔShort indicates the change in proportion of total transcript using the upstream 5′ss (resulting in a shorter exon). The sizes of the long and short forms of the alternative exon are indicated. (B) Table describing the top candidate exons with altered 3′ss usage after HiR treatment only, both treatments, or after HiB treatment only. Table contents are similar to (A), except that 3′ss were analyzed rather than 5′ss.

Supplementary Figure S7. (A) Table summarizing splicing-relevant information for the top candidate introns with improved removal after HiR only, both treatments, or after HiB only. Shown sequences correspond to 12 nt upstream and downstream of the 5′ss and 3′ss that flank the affected intron. The 5′ss and 3′ss scores are given. ΔIRM indicates the change in intron removal after treatment (positive numbers indicate improved removal, negative numbers indicate retention). The sizes of the upstream exon, affected intron, and downstream exon are given. (B) Table summarizing splicing-relevant information for the top candidate introns with increased retention after HiR only, both treatments, or after HiB only. Table arrangement is similar to (A). (C) Sample treatment key for panels D-H. (D-H) qPCR results measuring intron retained products (dark gray) or total gene expression (light gray) for candidate IRM introns affected by HiR only (D), both HiR and HiB (E), or HiB only (F), and candidate IRT introns affected by HiR only (G) or HiB only (H). Gene names and intron identities are indicated at the top of each chart. Y axis represents relative expression of each transcript normalized to *OAZ1* transcript and corrected relative to DMSO-treated control fibroblasts. Treatments are specified below. a: $p < 0.05$ for intron retained product, aa: $p < 0.01$ for intron retained product, b: $p < 0.05$ for total gene expression, bb: $p < 0.01$ for total gene expression.

Supplementary Figure S8. (A) Overview of hybrid minigenes used in this study. Lines indicate vector "back-bone" and introns, narrow boxes indicate vector promoter and restriction sites, and wide boxes indicate exonic sequences. Exon sizes are given above each exon; intron sizes, below each intron. Black octagon indicates transcription termination sequence. Abbreviations: prom, promoter. (B) The effect of HiR and HiB treatments on splicing of p*SMN2*ΔI6 minigene (left panel) and endogenous *SMN* exon 7 (right panel) in HeLa cells. Absence (-) or presence (+) of the minigene and small compound treatments are indicated at the top of the left panel. Splice isoform identities are indicated at the right side of the gel. Quantified percentage of included (FL) or skipped (Δ7) isoforms is given at the bottom of the gel. Abbreviations: CTR, control. (C) Splicing of the corresponding endogenous EIN exons in HeLa cells for the minigenes that were investigated in Figure 7. The compound expected to affect splicing based on results in GM03813 fibroblasts is indicated at the top. Other labeling and abbreviations are the same as in (B). (D) Splicing of the corresponding endogenous ESK exons in HeLa cells for the minigenes that were investigated in Figure 7.

Supplementary Figures S9-S12. Sequences of inserts used to generate minigenes. Cloning sites are highlighted in red. Exonic sequences are boxed. Predicted secondary structures of exons and their flanking intronic sequences are presented below their corresponding sequences. Secondary structures were predicted using RNA-fold WebServer: http://rna.tbi.univie.ac.at/cgi-bin/RNAWebSuite/RNAfold.cgi. Neutral numbering starts from the first position of exon. Positive numbering starts from the first position of the downstream intron. Negative numbering starts from the last position of the upstream intron. The 3′ and 5′ splice sites are marked as 3′ss and 5′ss, respectively.

Supplementary Figure S13. Splicing of pooled *FOXM1* exon 9 hybrid minigenes throughout the process of selection. Representative gel images showing splicing patter of *SMN2* minigene constructs are given in the lower panels. Treatments and minigene names are indicated at the top of the gel images. Splice isoform identities are marked at the right side of the gels. Densitometric quantification of each isoform's prevalence compared to total RNA expression is indicated at the bottom. Abbreviations: CTR, 0.1% DMSO control; InR, intermediate concentration of risdiplam (250 nM); InB, intermediate concentration of branaplam (10 nM); P0, pool 0 (pool of sequences before selection); P1, pool 1 (pool of sequences after first round of selection); P2, pool 2 (pool of sequences after second round of selection); P3, pool 3 (pool of sequences after third round of selection); P4, pool 4 (pool of sequences after fourth round of selection); D, selection in presence DMSO only; R, selection in presence InR dissolved in DMSO; B, selection in presence InB dissolved in DMSO; 9L, long form of *FOXM1* exon 9; 9M, intermediate form of *FOXM1* exon 9 due to activation of a cryptic splice site (Cr1); SL, short form of *FOXM1* exon 9; Δ9, exon 9-skipped product of *FOXM1*.

Supplementary Figure S14. Splicing of *SMN* minigene constructs with randomized exon 7 sequences in the presence of risdiplam and branaplam. Sequence of the entire exon 7 for each minigene construct are shown in the upper panels. Construct names are given on the left. Number of nucleotide substitutions within exon 7, type of treatment and percentage of minigene exon 7 skipping are indicated on the right. Mutated nucleotides are highlighted in white color on a black background. Three regions, Exinct, the Conserved tract and 3′ Cluster shown to affect

splicing of *SMN* exon 7 are indicated (13). C2 and C5 sites show where risdiplam C2 and C5 analogues interact with *SMN* exon 7 (45, 46). Representative gel images showing splicing patter of *SMN2* minigene constructs are given in the lower panels. Treatments and minigene names are indicated at the top of the gel images. Splice isoform identities are marked at the right side of the gels. "Δ7" indicates exon 7 skipping; "+7", exon inclusion. Densitometric quantification of exon skipping relative to total RNA expression is indicated at the bottom. Abbreviations: UNT, untreated; CTR, 0.1% DMSO control; HiR, high concentration of risdiplam; HiB, high concentration of branaplam.

Supplementary Figure S15. Differential expression of candidate genes in combined treatment of risdiplam and branaplam. (A) Key describing labeling of treatments throughout the rest of the figure. (B) Gene expression of select candidate genes as measured by qPCR after 6 hours of treatment with low concentrations of risdiplam and/or branaplam. Treatments are indicated at the bottom. Y axis represents relative expression compared to DMSO treated cells. Error bars represent the standard error of the mean. n = 3. *: $p < 0.05$. **: $p < 0.01$. (C) Gene expression of select candidate genes as measured by qPCR after 24 hours of treatment with low concentrations of risdiplam and/or branaplam. Labeling and statistics are the same as in (B).

Supplementary Figure S16. Time course of splicing of select candidate exons after HiR and HiB treatment. For each panel, representative gel images of RT-PCR of candidate exons are shown. Time and treatment are indicated at the top of the gel. Gene identity is given at the left side of each gel, splice isoforms are labeled at the right side. Lane number and percentage of skipping is indicated at the bottom.

Supplementary Figure S1

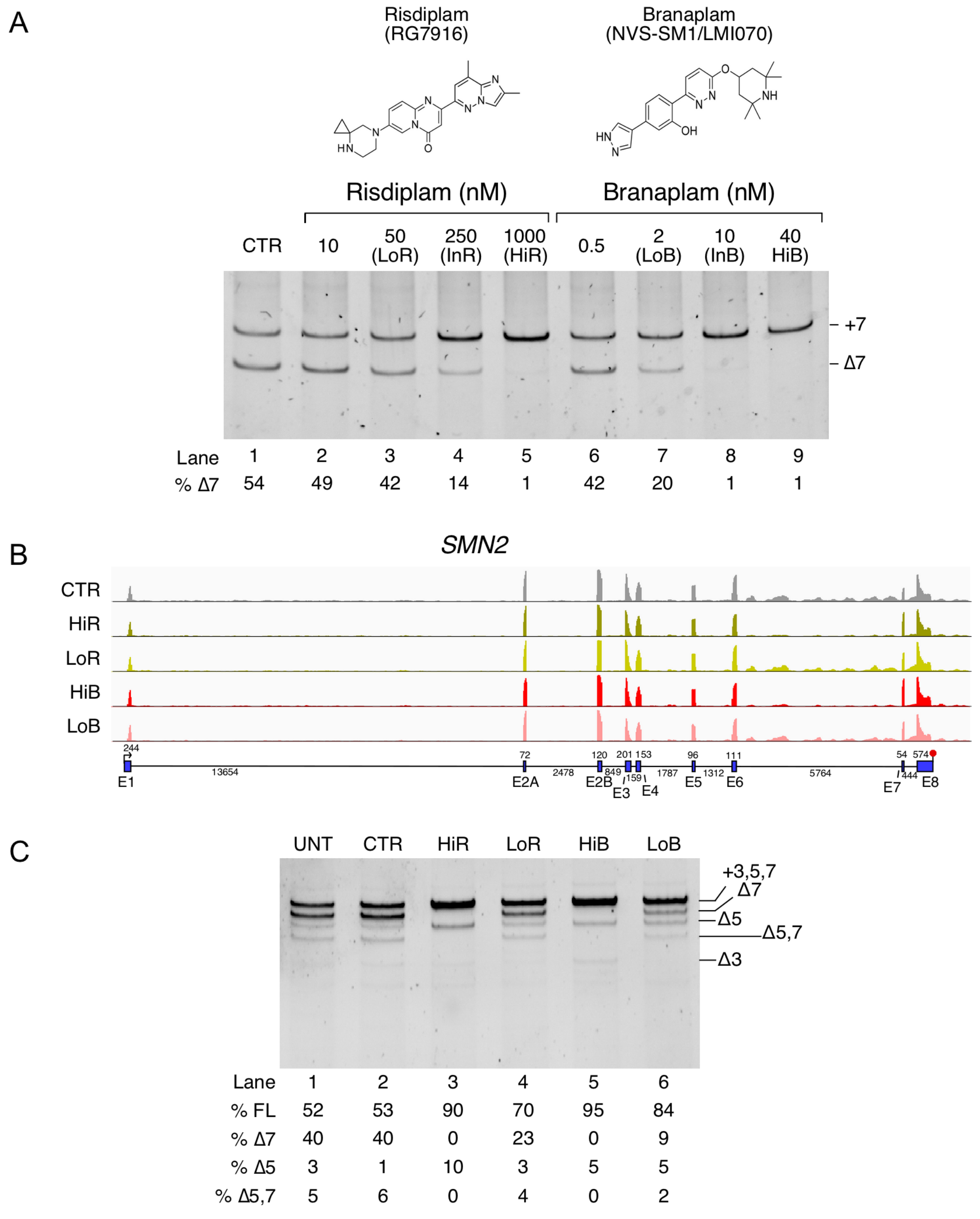

Supplementary Figure S2

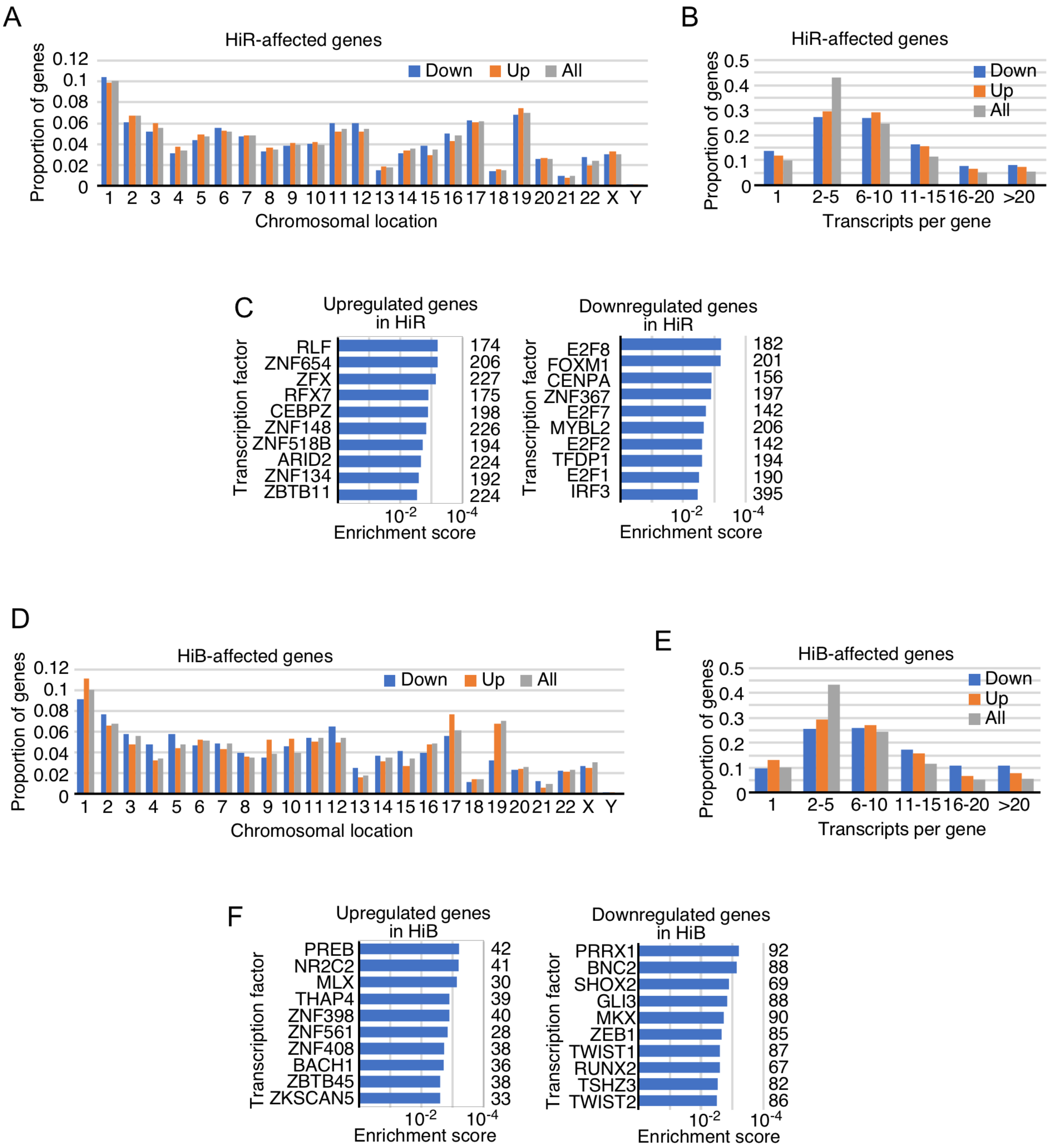

Supplementary Figure S3

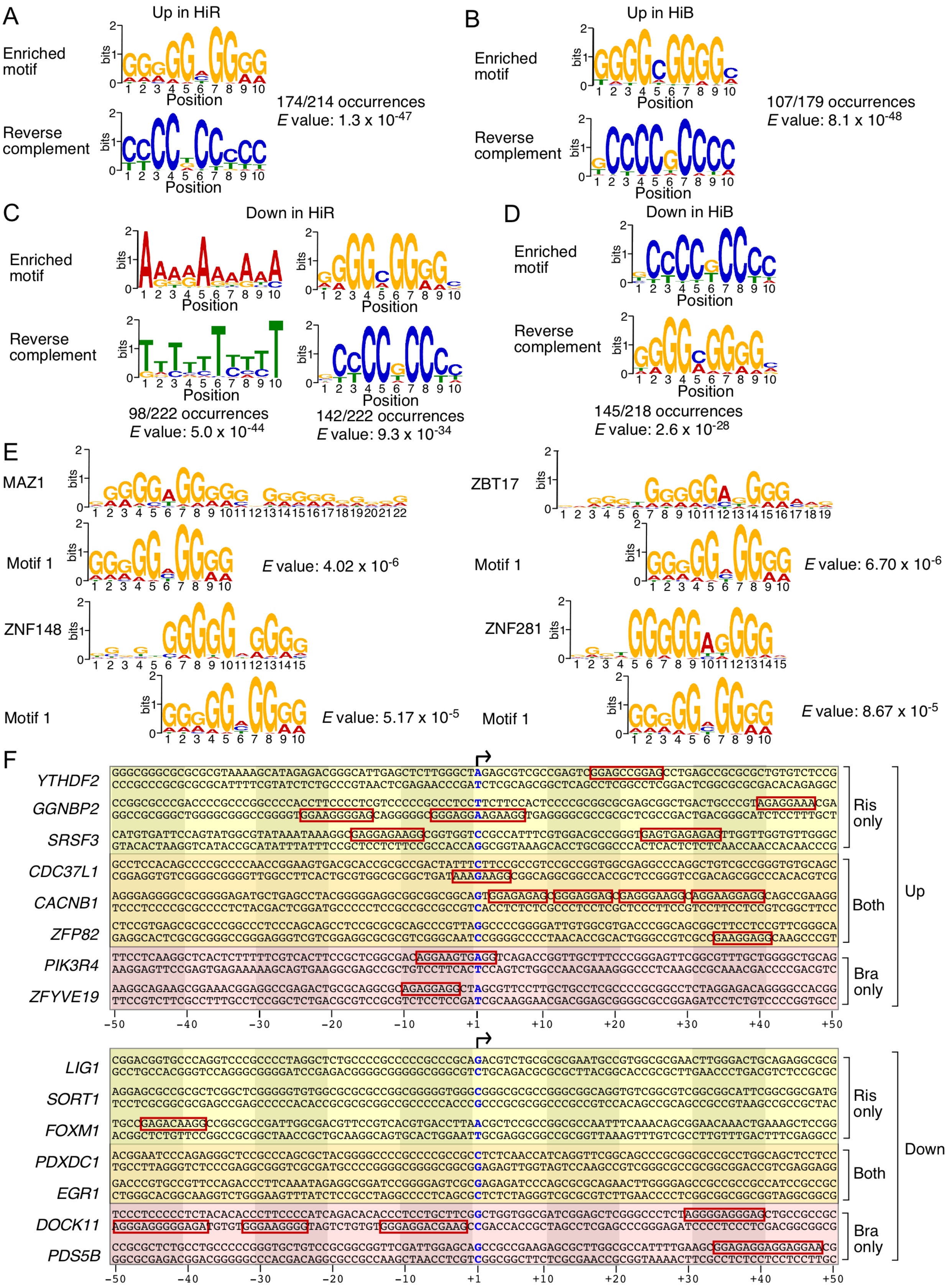

Supplementary Figure S4

A

Risdiplam targets: EIN 5'ss

Branaplam targets: EIN 5'ss

Targets of both treatments: EIN 5'ss

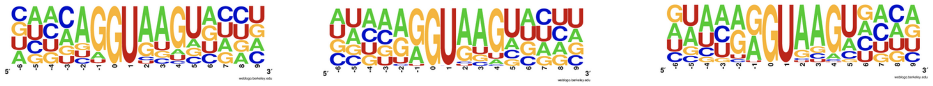

B

Gene/exon	Intron	Exon (3'ss side)	Exon (5'ss side)	Intron	HiR ΔSkip	LoR ΔSkip	HiB ΔSkip	LoB ΔSkip	3'ss score	5' ss score	Up Intron (nt)	Alt. Exon (nt)	Down Intron (nt)	
TTC28 E20	UGCUGGCCGAAG	GCUGUUAAAAAU	AUGGUUGGAAUG	GUGAGUACUACU	−0.66	0.04	0.03	0.08	1.7	10.4	1774	24	781	Ris only
EXOC1 E11	AAUCCAUCACAG	CUACACUGCCUC	AGGAAACAGAGA	GUGAGUAUGCUU	−0.54	−0.01	−0.05	−0.01	2.3	7.3	4959	45	1291	Ris only
MBNL1 E7	CUGCUAAUUAAG	ACUCAGUCGGCU	ACCUUUGACCUG	GUACUAUGACCU	−0.48	−0.15	−0.07	−0.11	−0.3	2.7	1164	54	862	Ris only
KSR1 E12	UUCUGUUUAAAG	CUGCCUACUUCA	UUAUCUUUCCAG	GUGAGUCCUUUG	−0.43	0.05	−0.06	−0.11	4.5	12.2	3924	42	2432	Ris only
RAPGEF2 E7	UUUGGCUCAAAG	GAAUGCACUAAA	ACUAAAUCUCAG	GUAUUGUAAUUU	−0.43	−0.05	−0.09	0.02	4.8	4.7	2406	18	60550	Ris only
NUMB E12	CUCCCGUUGUAG	CUAAUGGCACUG	CCACAUGUUCGG	GUAAGGUGGCCA	−0.42	−0.01	−0.04	0.00	0.6	8.8	2934	144	1987	Ris only
CLTCL1 E29	CUGUCUUCCUAG	GGCUUAAGGGCA	CAUCUCUACAAG	GUUGGUGGCCUG	−0.41	−0.21	−0.09	−0.14	9.1	5.2	252	171	3946	Ris only
HAPLN3 E5L	CUCUGUCUCUAG	GAUGGGGCUGUG	AACGGACAGGAG	GUCAGAGGUUUG	−0.40	0.00	−0.01	−0.01	9.5	3.6	746	1193	148	Ris only
CAMK2G E19	CUGGCUGGCUAG	CUGCCCCGCUCC	GCUCCUCAGCCA	GUAAGUGUGAGG	−0.40	−0.07	−0.03	−0.04	−0.2	7.3	2036	114	1977	Ris only
MGRN1 E16B	CUCUCCGCGCAG	CCCUGGGGCCCG	ACGAGUAAGCCG	GUACGUGACCUC	−0.40	−0.02	−0.02	−0.01	7.4	7.6	2326	45	2492	Ris only
POMT2 E11B	CAUCCUCCACAG	AUGUCCACUUAA	GGAGCAGAAAGA	GUAAGUGGCAGU	−0.71	−0.04	−0.95	−0.53	8.6	7.5	1490	39	410	Both
STRN3 E8	UGCUGCAUGUAG	AUAAAGAUGACC	AAGGGGUGAAGA	GUAAGUGCAGAU	−0.56	−0.03	−0.87	−0.33	4.0	7.5	5851	11	15543	Both
STXBP6 E4	CUUCCCCACUAG	AAUGUAAGCUCC	GAUAUUGAAUGA	GUAAGUGUGUAU	−0.55	−0.06	−0.67	−0.11	8.2	6.1	61905	87	13035	Both
SLC7A6 E4B	CUAAUUUCUUAG	GUAACCUGCCAU	GGGUGGGAAAGA	GUAGGUGAAAUC	−0.30	−0.04	−0.84	−0.24	4.3	4.5	12073	113	310	Both
LARP7 E2	CUUCUUUUGCAG	GACUCUGCCAUC	AGAUCUCAAAGA	GUAAGAUGCCAU	−0.40	−0.04	−0.92	−0.35	11.9	6.2	40	144	6637	Both
SLC25A17 E3	UUUUUUCCUCAG	AUUUGUUCAAGU	AUGAAGAGAUGA	GUAAGAUUAGCU	−0.39	−0.02	−0.59	−0.11	12.0	4.8	1686	53	2703	Both
AXIN1 E9	GUUGUUCCCUAG	GUAUGUGCAGGA	CUCCGAGACAGA	GUACGUGCGUCC	−0.28	0.00	−0.72	−0.13	7.7	4.8	2191	108	1582	Both
FOXM1 E9	UUAUUUCCAUAG	GUUUUUGGGGAA	CUUUGUUUAUCA	GUAAGUCUGAGC	−0.34	−0.05	−0.58	−0.09	8.0	6.2	2907	114	1635	Both
SENP6 E2B	UUUUUUAAAAAG	GCAUUCUGUUCA	CAUUUAUCAUGA	GUAAGUGAACCC	−0.31	−0.13	−0.56	−0.22	3.8	6.1	301	45	779	Both
ZNF618 E13	UCUCUUUUGCAG	AAACUGCAAGUC	CUCUCCUACAGA	GUAAGUGAUGGU	−0.23	0.03	−0.54	−0.09	10.9	7.5	2462	45	1064	Both
KDM6A E28	CCUUUUCUUUAG	GAUAUUUUCAUU	AUUCACAGGAGA	GUAAGAUAAUUG	−0.05	−0.02	−0.94	−0.24	10.9	6.2	15677	108	760	Bra only
PDE7A E2B	CAUUUGUUGCAG	CAUGAAGGAAUG	CAGAGAAACAGA	GUAAGCAGGAGU	−0.06	0.01	−0.86	−0.19	5.5	5.4	1835	104	1040	Bra only
MADD E21	CAUCUGGGGUAG	AAUUGUGGAACA	UGGAAAAACAGA	GUAAGGAACAAA	−0.11	0.01	−0.82	−0.13	−1.6	6.0	1726	54	1286	Bra only
TARBP1 E27	UCUAUUUCUCAG	GCAGUGUCUGGC	ACAUUUGUGCAG	GUGAGAUUUCAU	−0.09	−0.03	−0.81	0.02	9.3	10.9	232	96	529	Bra only
LYRM1 E4	CCUUUGAAUUAG	GUGAAGUAGUAU	AUCAGGGUCAGA	GUAAGUGCUCAU	−0.07	−0.07	−0.80	−0.12	3.0	7.5	8465	82	4291	Bra only
SH3YL1 E11	CUUUUUUGGCAG	GUAACAGAAAUG	AUGAGAGAGUUG	GUAAAUCAGUAA	−0.02	−0.05	−0.78	−0.03	10.0	5.8	5045	57	5862	Bra only
ABCB8 E2S	CCUGCCUUUCAG	AAACACAUGGCG	GGCAGAAGGAGA	GUAAGCGUCUCU	−0.03	0.01	−0.78	−0.22	9.7	5.4	529	51	2313	Bra only
TMEM50B E5B	UUUGCAUUUCAG	UGUUGGCCAUAG	AAAGAAAACUUU	GUAAGUUCAUUG	−0.02	0.00	−0.73	−0.04	6.3	5.8	4315	94	221	Bra only
ANXA11 E16B	AAUUCAUUACAG	AGUAUCUCCUGC	UGCACUCUUAGA	GUAAGUGUAGUG	−0.01	0.00	−0.68	−0.04	4.5	7.5	1142	121	465	Bra only
FAAP20 E4	CUGGUCUUCCAG	AAGCAAAAACAG	AGGCUGAGGCAG	GUGGGUUGCUGG	−0.06	−0.01	−0.66	−0.12	8.5	9.2	663	131	3063	Bra only

Supplementary Figure S5

Gene/exon	Intron (3'ss)	Exon		Intron (5'ss)	HiR ΔSkip	LoR ΔSkip	HiB ΔSkip	LoB ΔSkip	3'ss score	5' ss score	Up Intron (nt)	Alt. Exon (nt)	Down Intron (nt)	
DST E92	CAUUUUUAUCAG	UUCUUCCUGGGA	CUGGGAAAUCAG	GUAUAAACCAGU	0.63	0.07	0.03	0.01	5.0	5.1	883	18	2786	Ris only
TEAD1 E5B	UAUUGUAUCCAG	GUAACAAGCAUG	GUAACAAGCAUG	GUAAGUAUUUUC	0.62	0.10	0.07	0.08	6.3	10.6	13988	12	807	
BPTF E6	CUAUUUUUCUAG	AAGAACCUAACA	AGGAAGAGAUAG	GUAAGAAUAUAC	0.60	0.14	0.09	0.06	7.3	9.9	10383	189	5527	
THOC5 E10	UUCCAUCAUCAG	CUCAUAUGAAAU	CCCCUAGACAGG	GUGAGUAAUUUU	0.54	0.05	−0.08	0.02	5.0	10.2	720	33	1838	
GOLGA2 E3	CGCCUCCUGUAG	AUUCAGGACAUU	GACAAGUGGAAG	GUGAGGCAGUGC	0.47	−0.01	0.08	0.01	7.6	10.9	984	81	4260	
PLOD2 E14	UCUUCAUUUUAG	ACUUUACAAAGG	AGCCCCCCAAAG	GUGUUAUUUUUA	0.46	0.02	0.01	−0.01	7.8	4.9	1191	63	966	
MAP2K7 E2	UUCCCCAUCCAG	UUAUUGUGAUCA	CCCAACGAGCAG	GUACCAGCCUUU	0.38	−0.10	0.02	−0.10	7.6	4.6	1739	48	3899	
MFF E8	UUGUGUAAACAG	UGUGACACCAUC	UGAAAAUCGCAG	GUGAUUGGCCAU	0.37	0.05	−0.04	−0.04	1.9	8.6	4406	159	5129	
NFAT5 E4	UUUUUAUUCCAG	GAUUUGCCUCUG	UCUGCUUCAUAG	GUUAGAAACUUA	0.37	0.09	−0.03	−0.02	11.3	5.5	19988	62	450	
SPAG9 E28	UUCUCACUUUAG	CCGUAAUCCUCC	UGGGGCUGAGGG	GUAAGUGCAGAU	0.35	−0.01	0.08	0.00	6.9	9.4	1206	39	915	
WASF3 E8	UAUUUCCUUCAG	AGAGAGAAACAC	AGUGCCCAGCGG	GUUUGUUGUUUU	0.44	0.02	0.20	−0.03	9.8	3.3	3310	167	852	Both
BPTF E5	AUUUUGCUGUAG	AGCCAACAGAAG	AGGAUGUGCCAG	GUACAGAGGGCA	0.63	0.08	0.13	0.07	5.4	4.8	535	189	10383	
ODF2L E14	GCCACAACUCAG	AUGAGAGGCCAG	CUUCAGGGCCAG	GUGUCCAGCCGU	0.23	0.12	0.37	0.07	1.2	3.8	2184	159	1584	
FLNB E31	GCCACUGACCAG	GCCACAGAUGGG	UUCAGGCCCUGG	GUACAAUUUUGG	0.49	0.04	0.12	0.05	2.3	1.6	3328	72	720	
MAST2 E8	UUUUUACUACAG	CCAUAGCCACAG	CAGAGCUGACAG	GUAAUAGCGGGU	0.25	0.03	0.21	0.06	9.5	7.5	3159	21	1905	
KIF23 E8	UUCCUUUGGUAG	GUGGAACAGUUG	CACAGAUUUUGU	GUAUGUGAUGGU	0.26	0.07	0.20	−0.02	5.3	3.0	1952	42	746	
NFYA E3	UCCUGUUCUCAG	CAGCAGGGUGGU	ACCCUCCAGGUA	GUGGUACCCUCU	0.43	0.04	0.14	0.05	7.8	−1.6	1646	87	3148	
SNAPC5 E2	CUUCUUUUCUAG	GUUGAAGAAUUA	CAGUCACAUGAU	GUAAGCUUAAUC	0.28	0.15	0.17	0.10	10.7	6.1	2222	90	777	
AKAP13 E13	UGUCAUCCCCAG	UAUGAGCUGGUG	UAAUUACAGAAG	GUAUGAUAUUGU	0.25	0.17	0.19	−0.07	6.1	8.7	3797	66	2109	
AKAP13 E12	AUUCAUUUUCAG	UUCAAUGCGAGU	UCAUAGGAGAAG	GUACAGAGUUCA	0.23	0.19	0.20	0.08	4.4	5.0	2749	54	3797	
ARHGAP12 E17	AUUGUCUCUUAG	GUUUGGAUAUUG	CAGUCAAUCAUG	GUAAGAUUAUAU	−0.01	−0.01	0.57	0.03	6.1	9.3	2786	78	170	Bra only
KIDINS220 E2	CUAAUUUAACAG	GUAUUCAAAUAA	GAGAGAAAUGAG	GUAAGACCAAGU	−0.01	0.00	0.48	−0.02	4.2	10.3	10350	144	8192	
ATG5 E3	UUUUCAUUUCAG	UUGCUUUUGCCA	ACCACUGAAAUG	GUGAGUGAAUUU	−0.02	0.04	0.47	0.03	8.3	10.4	7609	128	15257	
PRKDC E62	AAUUUUUUACAG	AGGGACCCAAUA	UCUUGUAUUCAG	GUAAAGUCUGCA	0.01	0.00	0.42	0.03	6.6	7.5	2257	180	1306	
CNTN3 E8	UUUUCUUCUUAG	UCCCAUACCUCA	UCACUUACUAUG	GUGAGCAUCUGC	0.10	0.02	0.42	0.11	8.5	8.3	515	185	3456	
LRPPRC E19	UUUCAAUUUUAG	GAUGCUCACUUG	GACUUUCAAAAA	GUAAGUAGGAUU	0.00	0.00	0.18	0.00	5.7	9.7	346	5	360	
SNRPA1 E6	GUGUCGCUGUAG	GAGCGUCAGGAA	GAGAAGCAAAAC	GUAAGACACGGA	0.05	0.01	0.10	0.00	4.8	7.5	614	80	412	
ZNF207 E9	UUUAUGCUACAG	AUGGGGACACCU	AGCACAGCACAA	GUACGCAGGAAG	0.03	0.03	0.16	0.03	7.4	4.7	1177	80	1014	
VCAN E7	UUAUUUUUCUAG	CUAAAGAGGCUA	AAAAUAAGACAG	GUAAGUCUUUGC	0.03	0.01	0.15	0.01	6.4	12.4	6952	2961	14697	
MACF1 E47	CCUUCUGCUCAG	GUUCCAUACUGC	ACACCUGCAAAG	GUGAGAAUGCCA	−0.04	0.03	0.18	0.02	8.9	11.1	5864	80	679	

Supplementary Figure S6

A

Gene/exon	Increased 5'ss: Exon	Increased 5'ss: Intron	Increased 5'ss score	Decreased 5'ss: Exon	Decreased 5'ss: Intron	Decreased 5'ss score	HiR ΔShort	LoR ΔShort	HiB ΔShort	LoB ΔShort	Long exon (nt)	Short exon (nt)	
NCOR2 E47	UCACCUCGCCAG	GUCUGCAGGCCA	1.1	**AGGCACUCAUGG**	**GUAAAUAUGACC**	5.7	−0.52	−0.05	−0.03	0.00	225	87	Ris only
RALGAPA1 E18	**AACGAGCCAAAG**	**GUGCAGUUCCUG**	4.8	GGAAAGCUGCAG	GUGAGCACUAAC	10.1	0.50	0.07	0.06	0.02	1545	168	Ris only
NFATC4 E10	**GGGGUACCACAG**	**GUUGUGCCCAAC**	−0.1	CGCUGGAGGAAG	GUGGGUGUGGGA	9.4	0.28	0.06	−0.04	0.02	585	261	Ris only
UFD1 E2	**GCCUAAUGACAG**	**GUCAGAUGUGGA**	4.4	AAGGAGGGAAGA	GUAUGUGCUUGU	4.9	0.19	0.00	−0.02	0.01	133	107	Ris only
COL16A1 E66	UCCUGGCCUCAG	GUAAACAGAGGU	7.0	**CCUGGUCCCAAG**	**GUGAGUGGGCAC**	12.4	−0.11	−0.01	0.02	0.00	347	45	Ris only
CLEC16A E11	GGGAGAGUGAAG	GUGAGUGUCCCC	12.4	**AGAAGGCUAAAG**	**GUACAGAGGGUG**	5.0	−0.15	−0.07	−0.21	−0.02	232	184	Both
NOP56 E8	**GGCCCUGUUCAG**	**GUACCAGUGAGG**	4.6	CCAAGUUUCCAG	GUCAGCGACAUU	3.7	0.21	−0.01	0.14	0.04	281	101	Both
DDX51 E8	GCAGUCUCUACA	GUGCGUAGCAUG	4.6	**GACAGCCGCCAG**	**GUAUCCAGCAGC**	4.0	−0.15	−0.10	−0.17	−0.08	318	146	Both
GRHPR E1	CCGGGCGGCAGA	GUAAGAGCCUCG	6.2	**UACCCGCCGAGG**	**GUAGGGUCGCGC**	6.0	−0.16	−0.04	−0.15	−0.06	142	114	Both
SMARCC2 E28	GCCCACUGCCAG	GUGAGAAGGGGC	10.9	**CAGGCGUGGCGG**	**GUAAUGCUCCUU**	5.2	−0.09	0.07	−0.23	−0.02	429	84	Both
CNOT1 E19	**CAUUCCAGCAGA**	**GUAAGAUGAAAC**	6.2	AGAUGAAACCUU	GUAAGUGGUAGU	6.9	−0.04	−0.02	0.32	0.08	147	132	Bra only
TMEM208 E4	**GGGCAUGGCAGA**	**GUGAGUGUCCCC**	7.3	CCGCCAGCCCAG	GUGAGCGGCCCC	10.1	0.03	0.05	0.11	0.05	163	137	Bra only
SH3YL1 E9	CUAGGCAACAGA	GUAAGACCUUAU	6.2	**AGGAAGUCUUCU**	**GUACCGUUUGGU**	-2.3	0.06	0.02	−0.28	−0.01	645	169	Bra only
HDAC10 E1	GCUCUGGGACGA	GUAAGUGGGACC	7.3	**UCCCGGCCGAAG**	**GUGGGGACAGGC**	7.9	−0.14	−0.05	−0.37	−0.09	292	164	Bra only
PAXBP1 E8	**CAAGCCAUUCAA**	**GUCAGUCCAUCU**	2.8	UCCAUCUUGAAG	GUUUUUAUUAUU	2.0	−0.03	−0.03	0.33	0.06	141	124	Bra only

B

Gene/exon	Increased 3'ss: Intron	Increased 3'ss: Exon	Increased 3'ss score	Decreased 3'ss: Intron	Decreased 3'ss: Exon	Decreased 3'ss score	HiR ΔShort	LoR ΔShort	HiB ΔShort	LoB ΔShort	Long exon (nt)	Short exon (nt)	
RPL22L1 E3	CUCUAUUCCUAG	GGUACCUUCCCU	8.4	**GUUUUAAAAUAG**	**GAGCAAUUUCUA**	2.3	−0.28	0.02	−0.02	0.00	189	122	Ris only
DOCK7 E44	**UAGGAUGGUAAG**	**CGGAUGUUUGGC**	−8.6	UCCUAAACCUAG	GAUGGUAAGCGG	3.6	0.27	0.04	0.01	0.03	135	126	Ris only
MKNK2 E14	**UUUUUUCUGAAG**	**GUGGGACAGUCA**	8.9	CCCGUUUCCCAG	GAACAGCUGUGC	9.9	0.16	0.04	0.03	0.01	2386	359	Ris only
QKI E7	**CAAUUUAACUAG**	**GUGCGGUGGCUA**	0.3	UUUUAUCUUUAG	GUAUGGCUUUCC	9.1	0.11	−0.03	−0.02	0.01	850	75	Ris only
SNHG29 E3	**AUCUGACCGCAG**	**UCGCUGAAACCU**	3.2	UACUUGUAAUAG	GAAGUGCCGUCA	3.1	0.16	−0.07	0.03	0.01	176	123	Ris only
SREK1 E8	GUACUGCUGCAG	GGUGGCUGCACU	4.3	**UUUUCCUCCCAG**	**AGUCUGGAAAGA**	12.7	−0.25	0.08	−0.77	−0.12	290	115	Both
ELMO2 E3	**UUUGUCUCGCAG**	**AAUUAGAGCCCA**	10.1	CUUUCUCCCCAG	GCAGCCGUGUCU	13.3	0.29	0.05	0.46	0.23	128	99	Both
WDR91 E6	UAUUUUUAGUAG	AGAUGGGAUUUC	5.2	**UUUGCUGACCAG**	**UGCCAUGGUGUG**	3.8	−0.34	−0.02	−0.47	−0.04	1322	166	Both
SNAP23 E4	UCCCUUUAACAG	UAUUGGAAUAUG	6.6	**CCCUUGUCUUAG**	**UCUCAGGAUGCA**	4.8	−0.11	−0.10	−0.53	−0.03	274	49	Both
GMEB1 E3	UGAUUUAUGUAG	UUUGUUUAUCGA	1.1	**CUUUUACAACAG**	**GAUUUAUGAAGC**	8.7	−0.11	0.01	−0.11	0.00	113	83	Both
ANKRD13D E5	UCUUGACCCCAG	CCCAGGGUCACG	7.2	**GGUUUCUCUUAG**	**GCCCCCGAUUUC**	7.0	−0.02	0.00	−0.41	−0.08	930	46	Bra only
SLC44A1 E16	**AUUGAUUUGCAG**	**AUUAAGUAAUGC**	4.9	UUUGUUUUCUAG	GCUUCGGGAGCA	9.7	0.00	0.01	0.30	0.02	8314	7443	Bra only
ELF2 E7	UUUUUUUUAUAG	UGGAAGUGUUUG	9.2	**UAUCAACUCCAG**	**AAUUCAUCCAUG**	4.9	0.08	0.00	−0.20	−0.07	174	138	Bra only
EP400 E49	UCUACUGUUUAG	ACCCGGGUUCCC	4.2	**CCCACUUCUCAG**	**CUGCAGGCGCAA**	6.7	−0.10	0.00	−0.18	−0.04	196	175	Bra only
SART3 E5	UUGCUUCUUUAG	CCCGUAGCUGGC	6.5	**UUUGAUUCACAG**	**CUUGAGAAAGUC**	6.6	0.01	0.03	−0.12	−0.05	106	52	Bra only

Supplementary Figure S7

A

Gene/exon	Upstream Exon	Intron (5'ss)	Intron (3'ss)	Downstream Exon	5'ss score	3' ss score	HiR ΔIRM	LoR ΔIRM	HiB ΔIRM	LoB ΔIRM	Up Exon (nt)	Alt. Intron (nt)	Down Exon (nt)	
RBM5 I6	GAAGCCAAUCAG	GUUGCUUCACUC	CUUUUCUUACAG	AAAAAGUUGGUG	1.3	12.5	0.19	−0.02	0.01	−0.01	74	2477	84	Ris only
SPOCD1 I12	ACCACAGGACAG	GUGUGGGGUUGG	CCCUCCCUCUAG	GGUCCCUCCAUC	8.1	11.3	0.18	0.02	0.00	0.02	151	318	151	
MOK I8	CAAGUUCAAACA	GUAAGUAUUGUG	UUUUUAAUACAG	GUCGAGAGCUAU	7.6	8.5	0.16	0.04	−0.03	−0.08	102	979	174	
SPHK1 I2	UUAUGGAUCCAG	GUUUGUGGGGUU	UUGUUUUCUCAG	CGGGCGGCCCCC	5.4	8.4	0.15	−0.04	−0.04	0.01	204	330	153	
SMARCD3 I9	UAUUUCCAGCAG	GUAACCUCUCUC	GUCCUGCUUCAG	AUUUUUGAUUGU	6.6	6.3	0.14	0.04	0.01	0.02	162	969	98	
CLK4 I3	CAGUCACGUUCG	GUAUGAUUGGUU	UUGCCGUUCCAG	AAGAGCCACCGA	5.2	7.1	0.29	0.07	0.29	0.10	223	1121	91	Both
PNISR I10	AAGCAACGAAAG	GUAUAUUUUGGU	UGUGAUACAAAG	CUCCUGCAAAAC	6.6	−2.2	0.22	−0.01	0.19	0.01	100	720	54	
CLK4 I4	UAAGAGCAAGAU	GUAUAGAAUAUU	UCCUCACUCUAG	AUGAAAUCGUGG	0.7	8.3	0.22	−0.13	0.18	0.02	91	395	67	
NUDT22 I1	GACCUCUGGAUG	GUAGGGAUGCCC	AUCCUCCCCCAG	AGCUGCCCCGUU	6.1	8.7	0.19	0.04	0.13	0.02	154	207	498	
SLC17A9 I6	UGAGUGAAAAAG	GUAACGCAGGCC	UUGCUCCCUCAG	AUCUCAUCCUGG	7.4	9.2	0.15	−0.08	0.12	−0.03	131	519	97	
HNRNPM I6	GCAAUGCAAAAG	GUGAUGGCUACG	CAUGCAUUACAG	GCUGGAAGACUU	7.3	6.0	−0.13	0.00	0.18	0.01	39	117	36	Bra only
NFATC4 I10	CGCUGGAGGAAG	GUGGGUGUGGGA	CCUCCUUUACAG	UGAGUGAGAUCA	9.4	9.5	−0.12	−0.08	0.17	0.04	585	759	1967	
AHSA2P I5+6	ACUGUAAAGGAG	GUAAGUAACUCC	CUCUCUUUUCAG	UUAACAAAUAAG	11.6	9.7	0.11	−0.04	0.18	−0.04	117	827	52	
METTL17 I9	GGUCAUUUCCUG	GUGAGUUAAAAU	UUUUGUGAACAG	GUACUGGUGGAG	10.2	5.9	0.07	0.01	0.11	0.00	108	263	69	
ACADVL I11	AUUGCUAAGGCG	GUGAGUACCCUG	ACCCUCUUCCAG	GUAGAUCAUGCC	9.3	9.1	0.03	0.01	0.08	−0.01	199	267	105	

B

Gene/exon	Upstream Exon	Intron (5'ss)	Intron (3'ss)	Downstream Exon	5'ss score	3' ss score	HiR ΔIRM	LoR ΔIRM	HiB ΔIRM	LoB ΔIRM	Up Exon (nt)	Alt. Intron (nt)	Down Exon (nt)	
OGT I4	GAAGAAGCCAAG	GUAGGUGUUUGA	ACCCCUCCUAAG	GCAUGUUAUUUG	9.6	3.7	−0.38	−0.10	0.02	−0.01	69	3271	117	Ris only
C8orf33 I1	CGGCGCAUGGCG	GUGAGCGGUGUG	CCCUUUCUCCAG	GCCCUGGGACAU	7.2	12.7	−0.31	−0.10	−0.01	−0.02	31	88	312	
HMG20B I1	GUUUCUUUGGAG	GUGAAAACAGCC	UCCGUGUUCCAG	GUCCGGCCCGGA	6.6	7.9	−0.26	−0.08	−0.07	−0.02	79	297	56	
COG4 I8	UACCACCAGCAG	GUGAGCAGGGGA	GGUAUGUUGCAG	UUCCGGCAUGUU	10.1	1.3	−0.18	−0.01	0.06	0.02	158	766	59	
PTPRM I26	GUGCAAACAGAG	GUACUCCCGCUC	CUCCUUCUCCAG	GAGCAGUAUGUG	3.2	12.2	−0.16	−0.02	−0.04	−0.02	136	1667	150	
GGA3 I10	AUAACAGUUUGG	GUGAGUGAAAGA	UUCCUGCUCCAG	GGACAUCCUGC	8.8	4.8	−0.12	−0.04	−0.09	−0.02	82	98	117	Both
MAP7D1 I7	GCCGGGACCAGG	GUAGGGGCUGCG	AUCCCUCUGCAG	CCGUGCCGGUGU	6.0	6.7	−0.11	−0.04	−0.10	−0.02	123	96	164	
SLC25A3 I1	CCAUCUUAGGGA	GUGAGUGUGGCC	CUCCUCCCCUAG	AAAGAUGUUCUC	6.2	9.7	−0.13	−0.03	−0.07	0.00	55	234	161	
DENND5A I21	CCUGAGAAAGAG	GUGAGCCUUCCU	UUCCUUACGUAG	CGAGGCAGUCUG	9.3	5.2	−0.05	−0.01	−0.16	−0.01	83	557	124	
PKD1 I20	GUGCUGAACGAG	GUGAGUGCAGCC	UGCCUGCCCCAG	UACGAGCGGGCC	11.4	5.3	−0.07	0.08	−0.09	−0.03	160	390	153	
FBXO9 I9	GGAAUAUUACAG	GUACAACUGUAG	CUUAUUACAUAG	GUACAUAAGAUU	5.0	5.7	0.04	−0.02	−0.36	−0.04	91	641	96	Bra only
DGKA I10	UGAGCUGUAACC	GUGAGUAAUGGG	CUUCUCCCUCAG	UCUGUAAGUACA	6.5	9.8	0.08	−0.03	−0.35	−0.03	115	552	89	
SMTN I17	GGCGCUCGGAGA	GUAAGGCCACCU	CCUUUUUUGCAG	AUGGCAGUGGCA	6.0	11.6	0.02	0.00	−0.18	−0.06	63	208	70	
ARHGAP32 I20	AGACUCAGACAG	GUACCGUUUGUU	AUUUCCCACUAG	GAGCAGUUACCC	4.5	7.1	−0.01	0.01	−0.18	−0.01	879	691	980	
EXOC3 I10	CCGUAUAAGAAG	GUAAGAAGGUGG	GUGUCUCCCCAG	AGGAUGACGGCC	11.3	8.5	0.04	−0.01	−0.15	−0.01	123	698	162	

C

Controls	Risdiplam	Branaplam
❶ Untreated	❸ 50 nM (LoR)	❻ 2 nM (LoB)
❷ DMSO	❹ 250 nM (InR)	❼ 10 nM (InB)
	❺ 1000 nM (HiR)	❽ 40 nM (HiB)

D

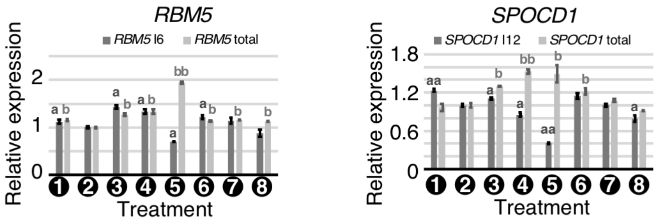

E

F

G

H

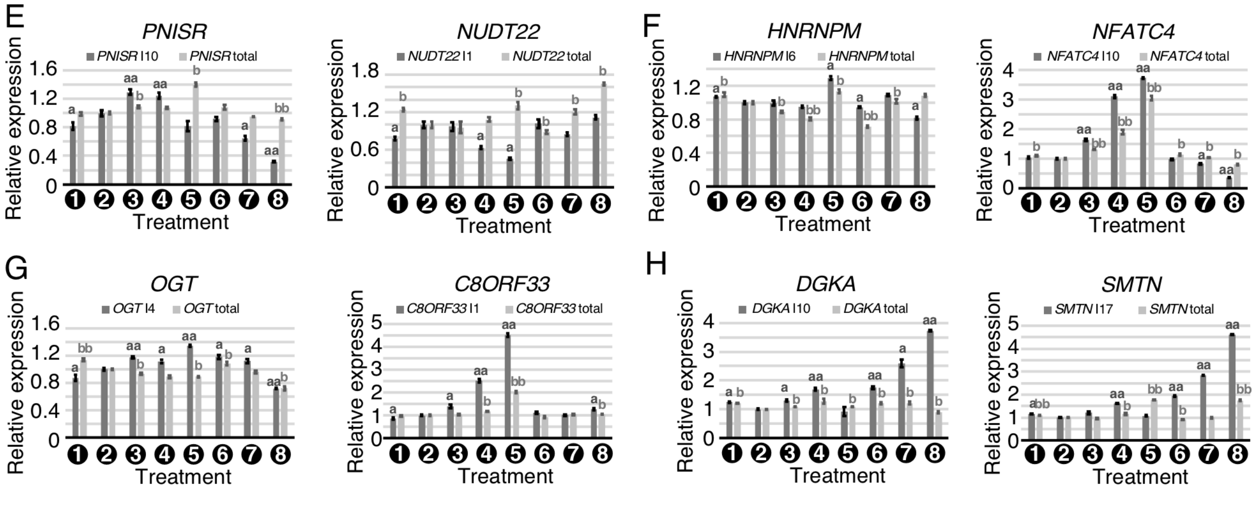

Supplementary Figure S8

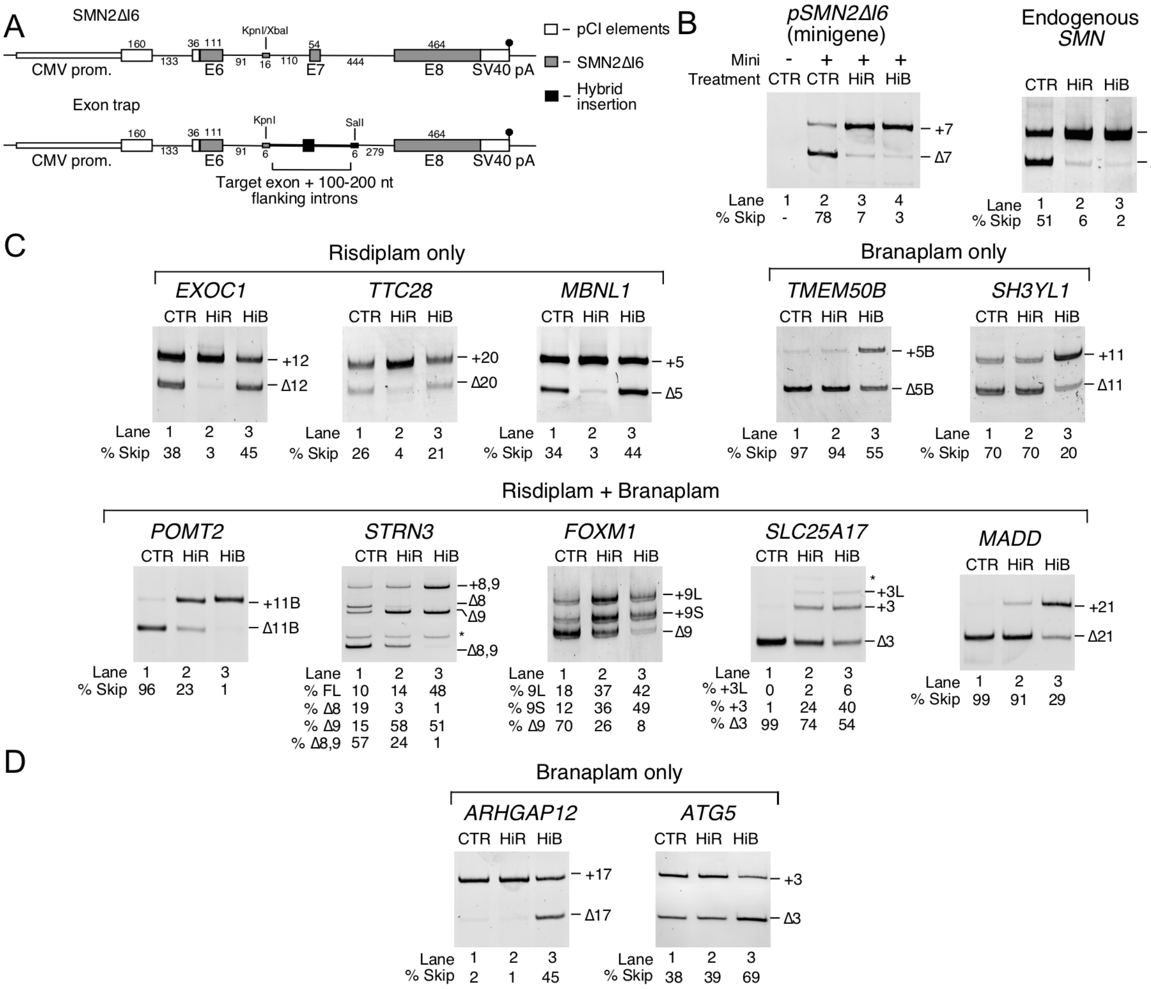

Supplementary Figure S9

EXOC1 E12

-136 GGUACCCUUGAUGCUCAUGGUUCUUUGCAUGUGUCUUUUAAACUAGAGCUUUAACUUCCUCUUGUGCAUGGUUUGUGCAAAUUGCAGUUU
-46 AUUAACUUGUCUUUGUCUUUGAUGCUUGCAACCUAAUCCAUCACAGCUACACUGCCUCGAAAAGAAAGUGCUGUCAAACAGGAAACAGAG
45 AGUGAGUAUGCUUAGUGUAUUAGUAGGUAUUCUCAAUGCAUGUUUGUUUAAUGUCUAGAAAUUAGUUGUCUUGAAAAAAGGUAACACCAA
+90 AAAUUUUGAUUGCUCUGAAUUGCCAGGUAUUGGUGAAAGAAAGCCAACAUGAUGGUAGUGAUAUGGCUGUAUCCAAGUCGAC

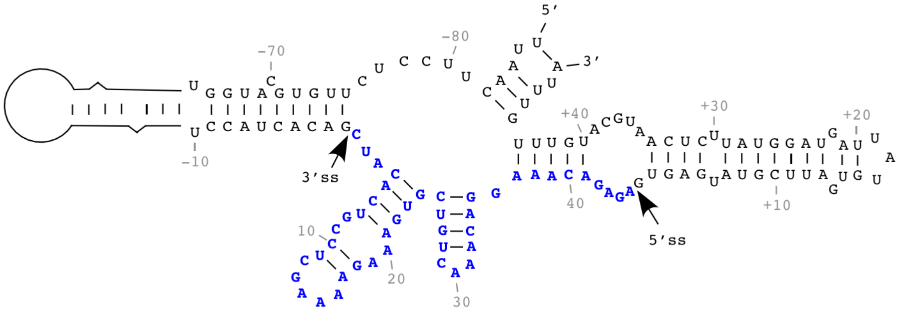

TTC28 E20

-163 GGUACCGCUGACUCUUGCUCCUCUCCCCUCCCAGUCAGAGCCGCGGGCCUGGUGUGGCGCUUCCUAAACAGUGAUCAGCUCAUGUUGCAU
-73 AACAGCCUGUGCUUGAGUCUAAACUCUCUUCUUUCUCUCUUUCUUUCUUUUUCUUAUAAUCUGCUGGCCGAAGGCUGUUAAAAAUAUGGU
18 UGGAAUGGUGAGUACUACUUUAAGUAGCAACUGUUGUCAGGUGAGCCCCUCAACCCACCCACCCCCGAGUUCCCCUGGGCAGGCUCUGAG
+84 CUCGGGGUGCACGUGCUGUCAAACGGAGCCACACGGGCUGCGGCGCCCCCGCGGGUGCGCACUGCUGCUGGACUGGCGGCCCGUCCCCUU
+174 CUCUGCUCUGCCUGUCGAC

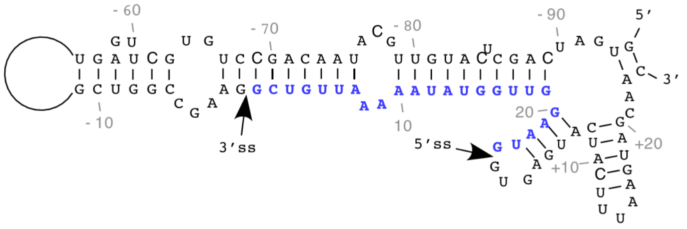

MBNL1 E5

-200 AGUAGUGCCUUUAUUGUGCAUGCUUAGUCUUGUUAUUCGUUGUAUAUGGCAUUCCGAUGAUUUGUUUUUUUAUUUGUUUUUUCUCACCUA
-110 CCCAAAAAUGCACUGCUGCCCCCAUGAUGCACCUCUGCUUGCUGUUUAUGUUAAUGCGCUUGAACCCCACUGGCCCAUUGCCAUCAUGUG
-20 CUCGCUGCCUGCUAAUUAAGACUCAGUCGGCUGUCAAAUCACUGAAGCGACCCCUCGAGGCAACCUUUGACCUGGUACUAUGACCUUUCA
+17 CCUUUUAGCUUGGCAUGUAGCUUUAUUGUAGAUACAAGUUUUUUUUUUUAAAUCAACUUUAAAAUAUAUAUCCUUUUUUCUGUUAUAGAGU
+107 UGUAAAGUACAAUGAAAAAACUGAGUGUGGUUUCCUGACAAAAUUAGUAGAAAGACUAUAAUCUAAGUACAUAGAUGGAUAUCAUACAAU
+197 AAAA

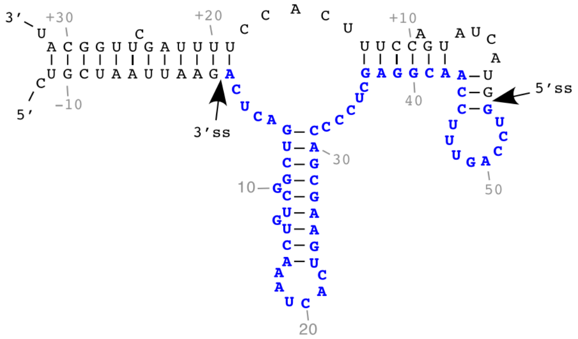

Supplementary Figure S10

TMEM50B E5B

-146 GGUACCCAGUCUGGGCAAUAUAGUGAGGCCCCAUUUCUUUAAAAACAAACACAAAAAACCUCUUGAAUUCAUGAAAGAAAAUAAAUUAAC
-56 AGACUGUUUAAAAGUCAAGAAUUUAUUUUGUAUGUUUUUACGAUUUUGCAUUUCAGUGUUGGCCAUAGGAGGCAAAGGAGCAAGUUACUG
35 CUUCCUCAGCAGUUUUGAUGUAGCUAGAACCAGCAAUUACUUUGACUAAAAGAAAACUUUGUAAGUUCAUUGACUUUUAUCCAGAGUAAG
+31 AGCAGUCCUUUGCUUUGGGGGAAUUAAAUUGGCAUUUUCUUCUUUCAGUUUCAUUAUAGAUGUCUUACAGUUAAUGAAUAAUAGCAGUAU
+121 GUUGUAAUAUACAAAAUGUAUCAUUAUGAAUGGCACUGAUACUUUAUUACUGCCGUUUGCCGUCGAC

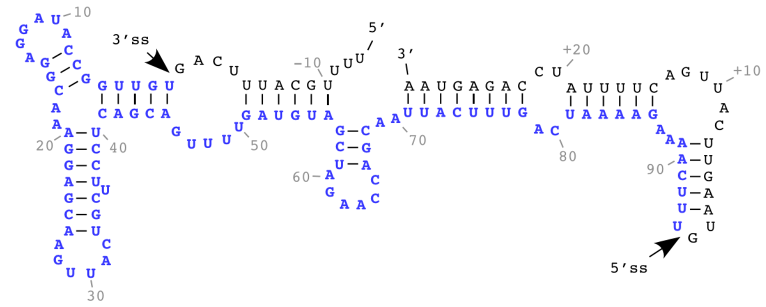

SH3YL1 E11

-193 GGUACCCUUCAUGUAUCUUCUGGCUUUAUUCAGGUAUACCUUGUGAUUUAAAGCGCAAUCAAAUGAUGAUGAUCCUAUCAACUUAGUGAU
-103 UUAAAAUAUUUGCUCAGCAAGGGCAGGAUCUGGCAAUUUUGUGUAUUUUGUAUGAUAUAUACUAAUCAGUUUUCAAAAUCACCACUCUCC
-13 UCUUUUUUGGCAGGUAACAGAAAUGAAUAUAAGCUCUAUCCUGGACUUUCCAGCUAUCAUGAGAGAGUUGGUAAAUCAGUAAAUCUAUAU
+21 GUUAUAAAUGAUUCAUAUUCAUUUUGUGAUCACAAACUUCCUAAUUAAUCUGACAGGUUAUGAAAAUCUCUGGGAAGUGUUCAGCCCUUU
+111 AAGUCGAC

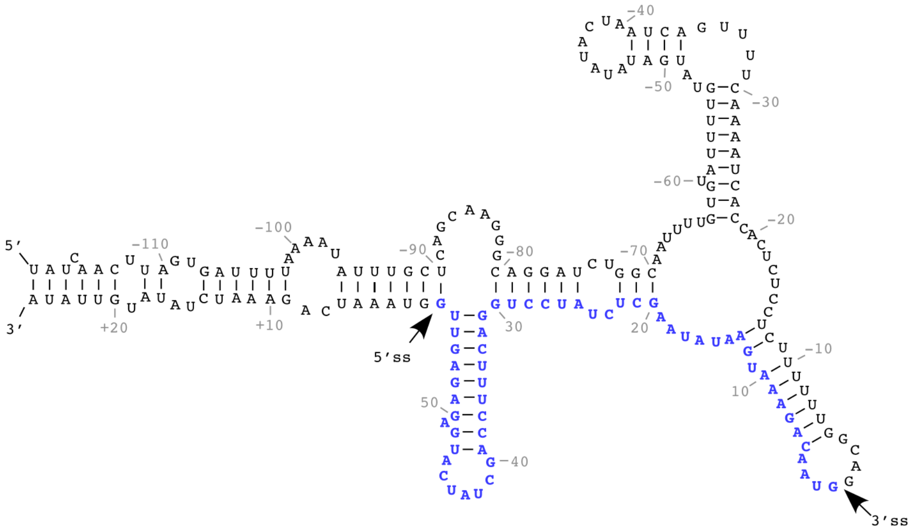

MADD E21

-193 GGUACCGUUCAUGAGUUUGGGUGGAGGGGUUAGAUCUCUUGUUUUCUGCCCCUUCUCCCCAUGAAGAUAUCUUUGUAUCUCUUGGUUGGU
-103 AAUCCAGUGCCUGAGACUGAAUCGACUUGGGUGGGUGGGUUUCCAUUUCGUCUGUCUGACCAGCCUCUGACUUUCUGUCUUUCUCUCCUG
-13 CUUGCAUUGCAUCUGGGGUAGAAUUGUGGAACAAGCACCAGGAAGUGAAAAAGCAAAAAGCUUUGGAAAAACAGAGUAAGGAACAAAUGC
+21 CCUUUCCUGUUCCCAAGUCCUCCAUACCUGCCAACUCCCCAGGCCCAGUUGGGGCAGGCUCCCUUUGUCAGCCCCACCCCAAAUUUGCUC
+111 UUAGAGCGUGUUUGGAAUGGCAGCAGGUAGGUAGCAUGUGACUCCUGUCGAC

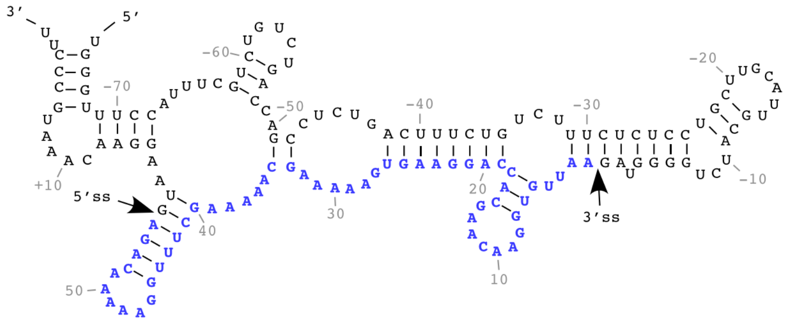

Supplementary Figure S11

POMT2 E11B

-213 GGUACCGAGUCAGCUGGAUUUUGAAUACUUAAAAUUUUUAUUUUAUUAAAUAAAUUUCAUUUGUCAUUAAUAAAAAUUUAUUGUAAUUAA

-123 AUUUUACUUUUGCAAAUUUUCAGAAAUUUUAUAAUGAACAUUUAUUAUUUGUUCCAGAUAAAAGUGAGAGUUGUUAUAUAAAACUAAAUA

-33 AAAGCUAAAUUAAACCCCUCUCAUCCUCCACAGAUGUCCACUUAAAAAAAUCUGGCGAUGGGAGCAGAAAGAGUAAGUGGCAGUGAGGAG

+19 GUUACCUAGCUCAGGCCAUGCAGAUUACAAGUUUUAAUUUUUCCUCUCCUCAAACCUGCCUCCUAAGUCAGGCAAGCUAAGUGCUUCAAA

+109 GCUACUAAAUGUGUGAAAGUCGCCCCCCACCUCCCUGCCAGCACCUAAAGUUAGCCGUCGAC

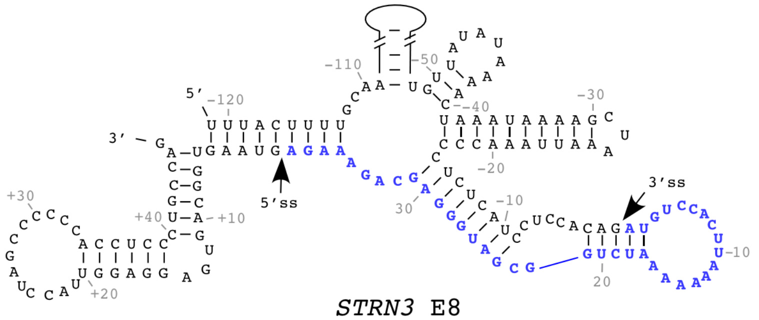

STRN3 E8

-159 GGUACCGAAUACCAGAACUGAGACUGCACCUUUAAAGUAUUUUGAGUAUAUGCUAUUAGUACAUUUUAUUAUGAUUAUGUAAAGCUAUAA

-69 CAGUUUACUGCCAACAGCUUGCAUUGCCAACUGCUGAUUUUUUUUCUUUUAAUAAUAUGCUGCAUGUAGAUAAAGAUGACCUCUCCCCAA

22 CUGCUGAGGUUUGGGAUGUAGACCAGGGACUAAUAAGUAAACUGAAGGAACAGUACAAGAAGGAACGAAAGGGGAAGAAAGGGGUGAAGA

+1 GUAAGUGCAGAUUCUGACUAUUAUAAUUUUUGUAUACCAAUAAUUGAGAAAAAAGUUCUUCAAUUCUGUUUCUUUGUGUAACUUAAUUUU

+91 UUGUUUCAUAAAGGGGGCAACUAACUUUAAACUUUGUCUGUUGUAUAAUUGAAAUUUGUUUGUAUAUAAUUUAGGAUGUGUUAUUGAUCA

+181 AGUAGGUCGAC

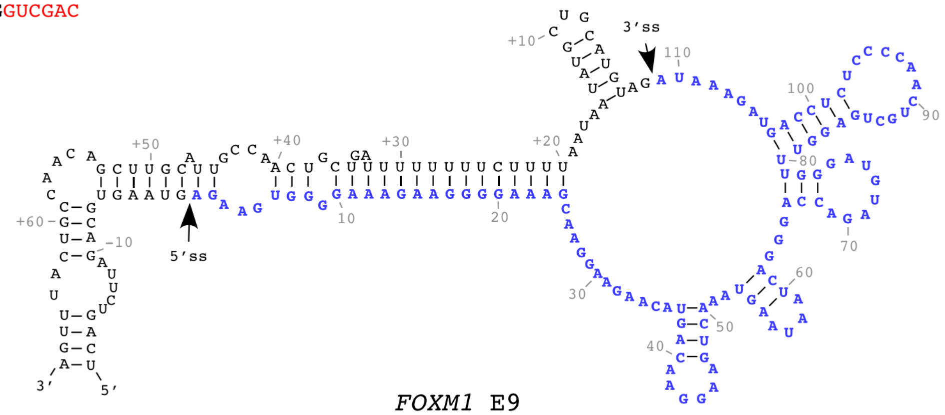

FOXM1 E9

-196 GGUACCAUAUUGCUAGAUUGCUUUCCAAAAAAGCUAUGAUAAGUCAUGUUUAAACCAGCAAUAUAUGAUAAGAUCUAUAUCUUUGCCAG

-113 CAAUUGGUAUUAUUAGUCUGUUUAAUAUCUGCCAAUCUAAUAUGUAUAGGUUAUAUCUUCUUAAUUUUUUAAAAUGUUUUAUUUAUUUA

-24 UUUUUAAAUUUUUUAUUUCCAUAGGUUUUUGGGGAACAGGUGGUGUUUGGUUACAUGAGUAAGUUCUUUAGUGGCGAUCUGCGAGAUUU

66 UGGUACACCCAUCACCAGCUUGUUUAAUUUUAUCUUUCUUUGUUUAUCAGUAAGUCUGAGCACCUUGUCAUUAUGUUUGGUGACUUGGA

+37 AUUGUCCUCUAUGAAUUGGUUAUUUAUGAACAUUGCCCAUUUUUAUAUAGGUUUUGUUAUCAAUUUGUGAGAGCUCGUCGAC

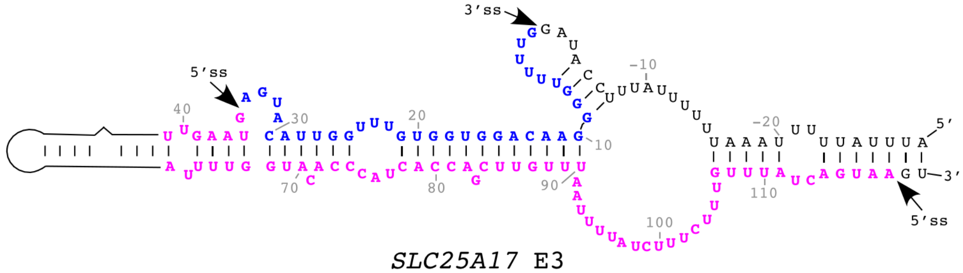

SLC25A17 E3

-134 GGUACCAAGAGGCAGAAUAGGAACAUACUGGAGCCACAUACUCAAUGCCAUUGCUAAUGUAGGCAUGUAGUCCUAAUCUGCCUUUGCAGC

-50 UUUAUGGGACUCUACAACUCACUGUGAUUCUUGCUUGUUUUUUCCUCAGAUUUGUUCAAGUUGAAAUUGUAAACCUAUGCCAGAACUUGC

41 AUGAAGAGAUGAGUAAGAUUAGCUUUCAAUAGUAAUUCAAAGGUUAAACCCUUUAUGCCUACUAUUUUUGGGUAAUUUUAUAGUUUUUGG

+49 AUAAAUAAUUUUACAUGAUUCAACAGGCAAAAUAUAUAAAAAGGUAUACAGUGCAGUGAAAAUGCUCACUCUUAGUCUGCCCUGUCUCCC

+139 UUUCCUCUUCACUGCAGGGUCGAC

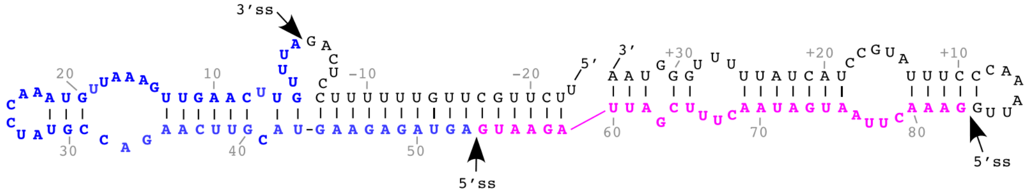

Supplementary Figure S12

ODF2L E14

-135 GGUACCGUUGUGUUCUUAUUUAAAGCCAUUAUACCUAAAAUACUUACGUUAUAAGCAUAUCUAGUAUUUAAGAUAAUAAAAAUAUUAAUG
-45 CUGAAUGUGUGAUCUUGCACUUAUUUCCCUACUGCCACAACUCAGAUGAGAGGCCAGAUGGAGUCUCAUCUGAAGGAGUUAGAGCGUGUC
46 UGCGAUUCCUUGACGGCGGCGGAACGGAGGCUUCACGAGUGUCAGGAGAGUCUGCAGUGCUGCAAGGGGAAGUGUGCAGACCAGGAACAC
136 ACCAUUAGGGAGCUUCAGGGCCAGGUGUCCAGCCGUCCUUCUGAGUUUUGCUAGAUGCGUUAAAAGAAUGCUUUGGAAUGAUUUCACCAG
+67 UUGAGGGCACUCAUGUCAGGGCUUGAUGUCGAC

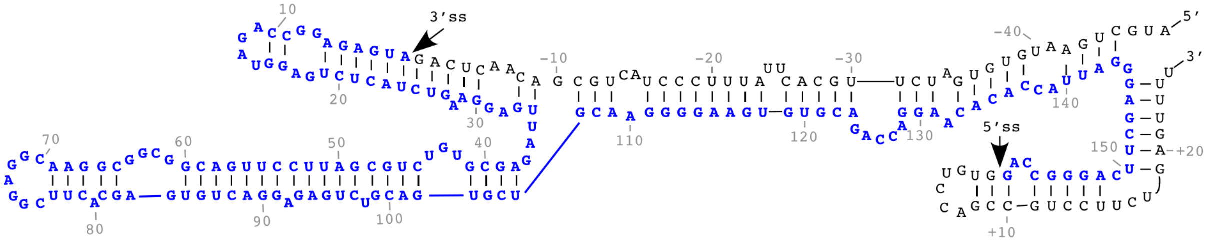

ARHGAP12 E17

-213 GGUACCCUACCAGAAGCCAUUUAGAGAAAAAGAAUAUUUGCCCAUUUGAUUUUAGGCUAAUGCAAAUGAGUGGGAAUUCUGACACAUGCA
-123 AACGUGUUCAGAAGAGGCAAAGAAGUACUUUAAACACACACACACACAAAUUAUUAUUGUCUCUUAGGUUUGGAUAUUGAUGGGAUAUAC
-33 AGAGUAAGUGGCAACCUCGCAGUGAUCCAGAAACUAAGGUUUGCAGUCAAUCAUGGUAAGAUUAUAUUUACUGUUGUUAUUCAGAUGCAU
+19 CACUCAUAACGUCGAC

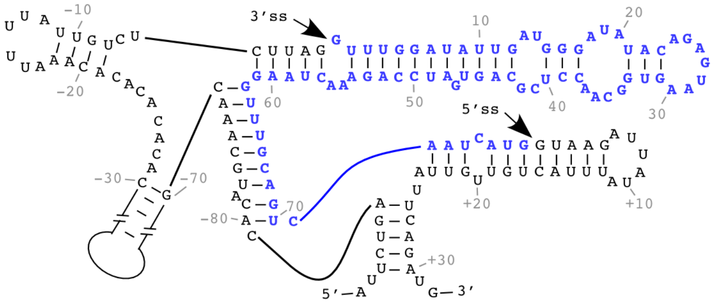

ATG5 E3

-144 GGUACCAAGCAGUAGACUUUGUGUGGGAUAUUCAAAAUAAUGCAAAGAACACGGCUGUUUUUCCUUAAAAUUCAAAAACCUACUUAGUAU
-54 UUUUUAAAAAUAAUAACUAGUAAAUAAAUACGGUUUUACAAAUUUUCAUUUCAGUUGCUUUUGCCAAGAGUAAGUUAUUUGACGUUGGUA
37 ACUGACAAAGUGAAAAAGCACUUUCAGAAGGUUAUGAGACAAGAAGACAUUAGUGAGAUAUGGUUUGAAUAUGAAGGCACACCACUGAAA
127 UGGUGAGUGAAUUUUUCUGCAUUAUUAAGCAUUAAGUAUAUACUAGCUUUAAAAAGGUAUAGUGCCCUGAAACAAUUGUAAAAAAAGUCC
+89 UGCGAGGAUAGAAGUCAUUAGUCUAGCUCAUACAAUAUGUGAAGUUUAGACUAUUACUAGAAUACGUUAUUGAAAAUAUUUCUGCCUUCU
+179 GUUUGGAUGCAUGGAUGACUGGGUCGAC

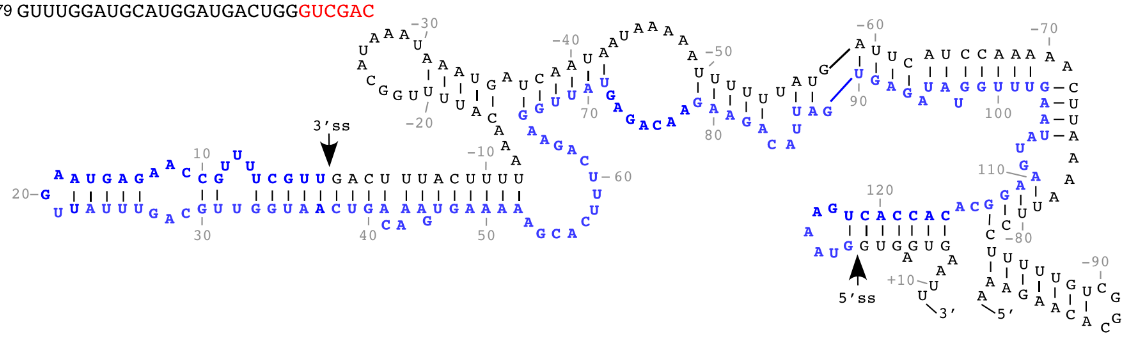

Supplementary Figure S13

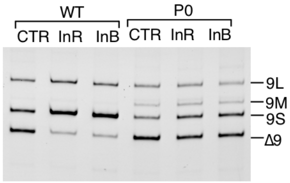

	1	2	3	4	5	6
% 9L	19	29	23	15	14	9
% 9M	0	0	0	5	7	5
% 9S	37	60	66	21	34	39
% Δ9	44	11	11	59	44	47

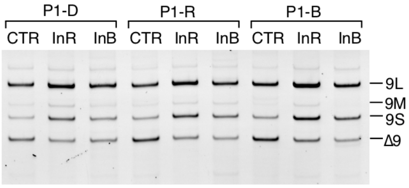

	7	8	9	10	11	12	13	14	15
	52	61	57	43	52	54	49	58	55
	1	3	1	2	2	2	0	1	0
	12	22	19	14	29	27	13	27	31
	36	15	23	42	17	17	39	14	1

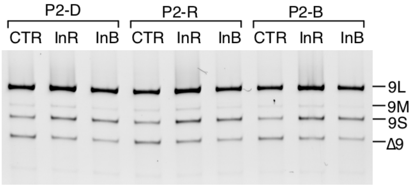

	16	17	18	19	20	21	22	23	24
	71	72	73	65	67	68	71	70	70
	1	2	1	2	2	1	1	2	1
	12	18	18	14	20	22	10	20	20
	16	9	8	19	11	10	18	9	8

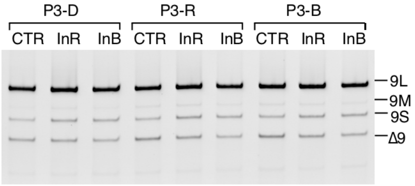

	25	26	27	28	29	30	31	32	33
% 9L	80	80	80	76	75	78	75	77	79
% 9M	1	1	1	1	1	1	1	1	1
% 9S	6	9	10	7	12	11	8	10	10
% Δ9	13	10	9	16	12	10	16	12	10

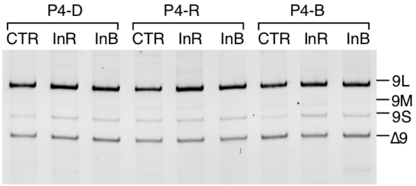

	34	35	36	37	38	39	40	41	42
	74	74	70	69	77	73	65	69	67
	0	0	0	1	1	0	0	1	0
	2	4	4	4	4	6	2	7	5
	23	22	26	26	19	21	33	23	28

Supplementary Figure S14

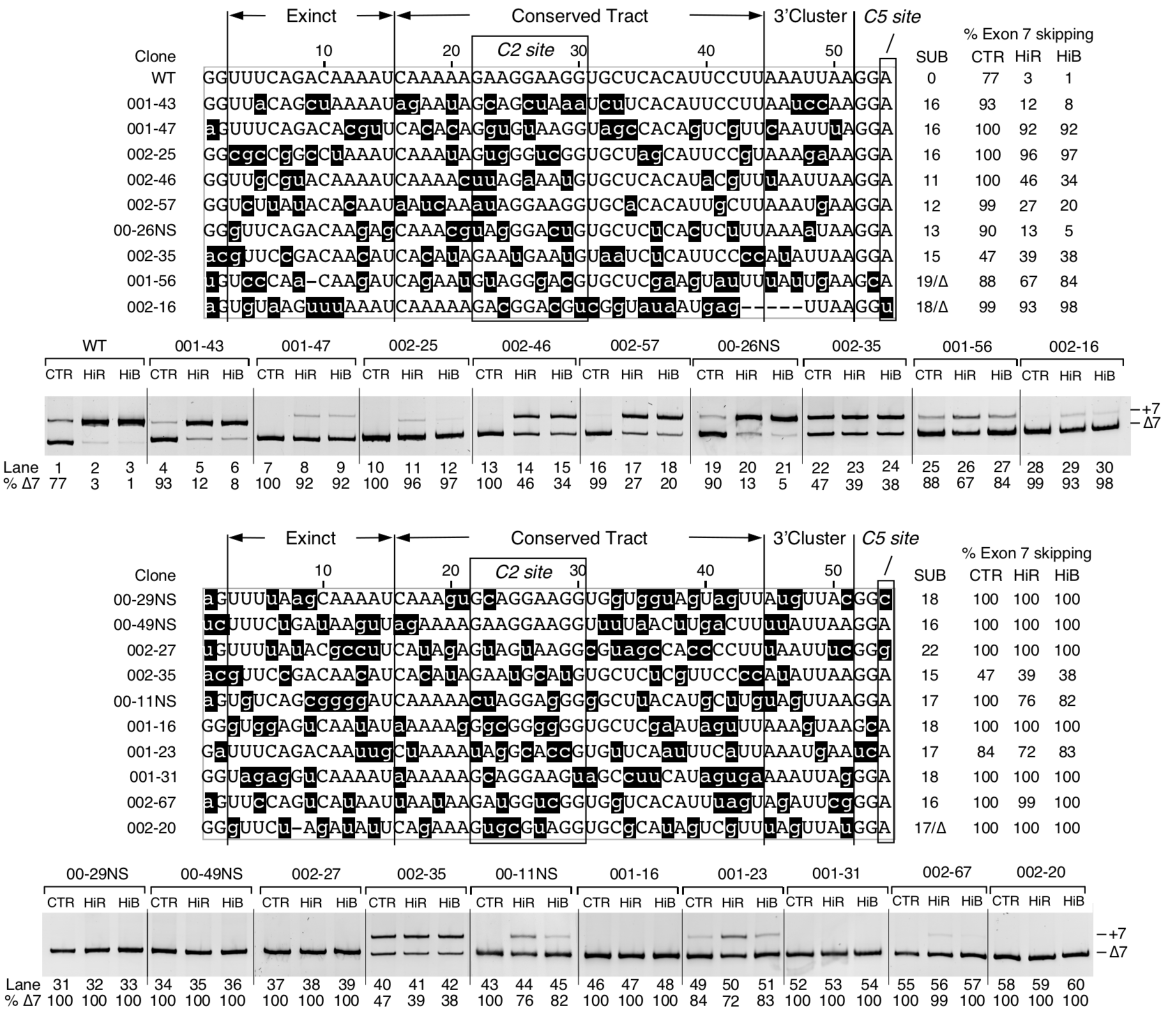

Supplementary Figure S15

A

❶ 0.1% DMSO
❷ 50 nM risdiplam
❸ 100 nM risdiplam
❹ 2 nM branaplam
❺ 4 nM branaplam
❻ 50 nM risdiplam+2 nM branaplam
❼ 50 nM risdiplam+4 nM branaplam
❽ 100 nM risdiplam+2 nM branaplam
❾ 100 nM risdiplam+4 nM branaplam

B 6 hours treatment

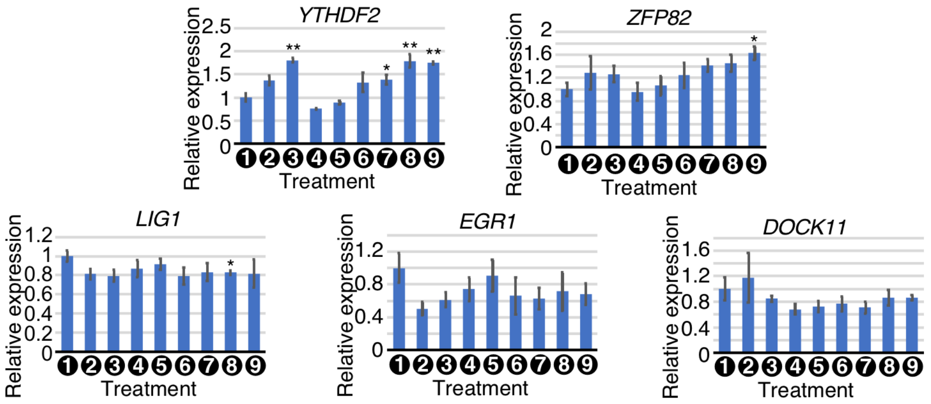

C 24 hours treatment

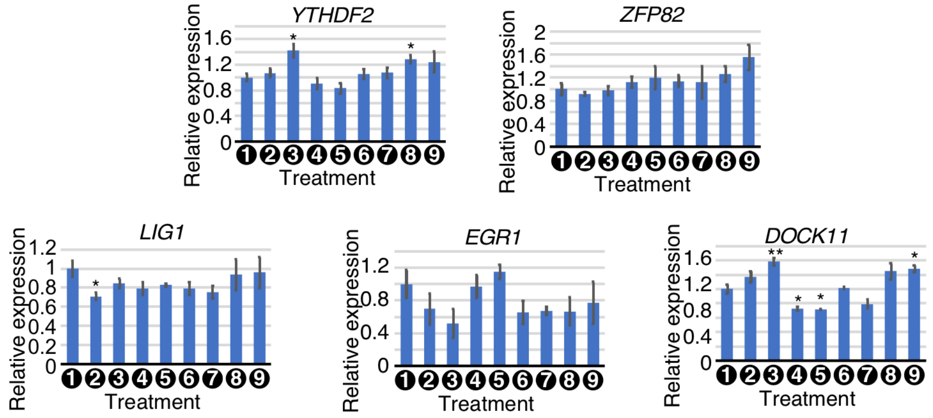

Supplementary Figure S16

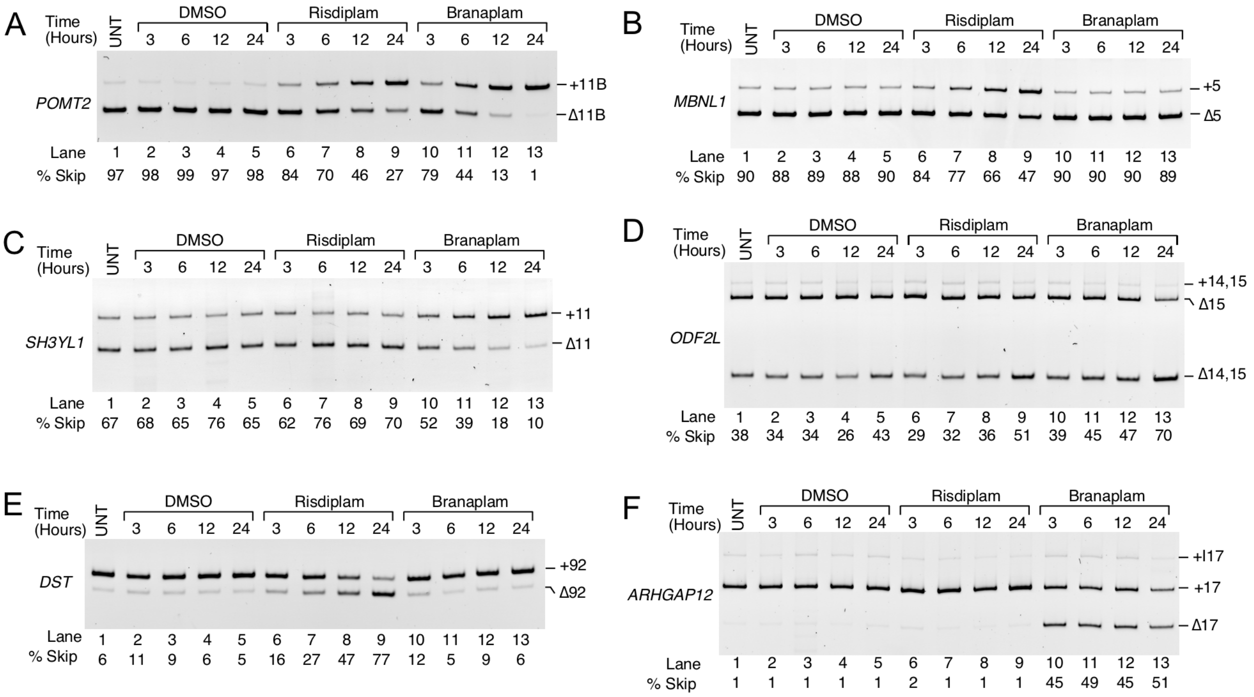

Supplement: gkad259_Supplemental_Files [file gkad259_supplemental_files.zip › Supplementary data_3-13-2023.pdf]
